# The liposoluble proteome of *Mycoplasma agalactiae*: an insight into the minimal protein complement of a bacterial membrane

**DOI:** 10.1186/1471-2180-10-225

**Published:** 2010-08-25

**Authors:** Carla Cacciotto, Maria Filippa Addis, Daniela Pagnozzi, Bernardo Chessa, Elisabetta Coradduzza, Laura Carcangiu, Sergio Uzzau, Alberto Alberti, Marco Pittau

**Affiliations:** 1 Dipartimento di Patologia e Clinica Veterinaria, Università degli Studi di Sassari, Sassari, Italy; 2 Porto Conte Ricerche Srl, Tramariglio, Alghero (SS), Italy; 3 Dipartimento di Scienze Biomediche, Università degli Studi di Sassari, Sassari, Italy

## Abstract

**Background:**

Mycoplasmas are the simplest bacteria capable of autonomous replication. Their evolution proceeded from gram-positive bacteria, with the loss of many biosynthetic pathways and of the cell wall. In this work, the liposoluble protein complement of *Mycoplasma agalactiae*, a minimal bacterial pathogen causing mastitis, polyarthritis, keratoconjunctivitis, and abortion in small ruminants, was subjected to systematic characterization in order to gain insights into its membrane proteome composition.

**Results:**

The selective enrichment for *M. agalactiae *PG2^T ^liposoluble proteins was accomplished by means of Triton X-114 fractionation. Liposoluble proteins were subjected to 2-D PAGE-MS, leading to the identification of 40 unique proteins and to the generation of a reference 2D map of the *M. agalactiae *liposoluble proteome. Liposoluble proteins from the type strain PG2 and two field isolates were then compared by means of 2D DIGE, revealing reproducible differences in protein expression among isolates. An in-depth analysis was then performed by GeLC-MS/MS in order to achieve a higher coverage of the liposoluble proteome. Using this approach, a total of 194 unique proteins were identified, corresponding to 26% of all *M. agalactiae *PG2^T ^genes. A gene ontology analysis and classification for localization and function was also carried out on all protein identifications. Interestingly, the 11.5% of expressed membrane proteins derived from putative horizontal gene transfer events.

**Conclusions:**

This study led to the in-depth systematic characterization of the *M. agalactiae *liposoluble protein component, providing useful insights into its membrane organization.

## Background

Mycoplasmas are the smallest and simplest prokaryotes capable of self-replication, being provided only with the minimal machinery required for survival. During evolution, they have regressively evolved from gram-positive bacteria by reduction of their genome to an essential minimum, economizing their structural elements, metabolic pathways, and genetic resources [1].

Among other consequences, this cost-cutting strategy led to loss of the cell-wall component, and therefore to lack of a peptidoglycan "shell". Instead, sterols are incorporated into the lipid bilayer, providing resistance to rupture, but still allowing a certain flexibility of cell shape. Integral and associated membrane proteins are therefore directly exposed and act as the immediate bacterial interface, playing a major role in survival and pathogenesis [2,3]. Gathering information on membrane proteins of such a pathogen might provide novel and interesting insights on its biology, and generate useful information for improving diagnosis, vaccination, and therapy. Recently, a large-scale study was carried out on the proteome of the human pathogen *Mycoplasma penetrans*, based on the TAP-MS approach [4]. However, membrane proteins were not included in this study, since they require dedicated protocols for purification and analysis and present numerous challenges.

Many members of the genus *Mycoplasma *are pathogenic for humans, animals, plants, and insects. *M. agalactiae *is the etiological agent of Contagious Agalactia (CA), a serious disease of sheep and goats characterized by mastitis, polyarthritis, keratoconjunctivitis, and abortion [1,5,6]. CA has a worldwide distribution and is endemic in Mediterranean Countries [7], causing severe economic losses in areas where economy is largely based on small ruminant milk production [5].

In Europe, the disease has been tentatively controlled either by vaccination or with serological tools based on recombinant surface proteins [8-13]. At present, the two above mentioned strategies are not actually compatible until proper DIVA (Differentiating Infected from Vaccinated Animals) vaccines will allow discrimination of vaccinated animals from naturally infected ones. The highly immunogenic, surface-associated membrane proteins represent key antigens for diagnosis and vaccine development. However, the finding of constantly expressed surface proteins in mycoplasmas is complicated by the existence of mechanisms aimed to evade the host immune response [1,14-17]. Surface-associated proteins are also pivotal for pathogenesis by acting as cytoadhesins [18]. To date, a limited number of constantly expressed surface proteins have been described in *M. agalactiae*. Among them, P30, P48, and P80 were described as antigens [19-21]; other proteins belong to the variable surface membrane proteins family (Vpma) [14,17], and P40 was suggested to play an important role in attachment to the host cell [18].

Genetic approaches traditionally used for large scale investigation of protein sets have been poorly applied to mycoplasmas. The expression of immunogenic Mycoplasma proteins in *Escherichia coli *expression libraries is hampered by the very high A+T content (almost 80%) and by the Mycoplasma-specific codon usage, resulting in abnormal internal transcription/translation and in premature termination, respectively [22,23]. In 2007, the full genome sequence of the *M. agalactiae *type strain PG2 (PG2^T^) was published [24] and paved the way for systematic proteomic studies in mycoplasmas.

The combination of 2-D PAGE and mass spectrometry (MS) is a well-established method for the systematic and comparative study of proteomes, since it allows the simultaneous visualization and identification of the protein complement of a cell. However, it is commonly reported that standard 2-D PAGE lacks in resolution of very hydrophobic and basic proteins, which are particularly abundant in the *Mycoplasma *membrane [25-27]. Indeed, membrane proteins are poorly detected in 2-D PAGE maps of *Mycoplasma *total protein extracts [22,28]. Triton X-114 fractionation may assist in solving this problem, since it was demonstrated to enable a selective enrichment in hydrophobic proteins [29,30]. Triton X-114 fractionation followed by 2-D PAGE remains the method of choice for proteomic characterization of the membrane protein subset [31], and for differential analysis of membrane protein expression among bacterial strains [32]. More specifically, the recently developed Differential In Gel Electrophoresis (DIGE) [33-35], based on labeling of protein samples with fluorescent dyes before 2-D electrophoresis, enables the accurate analysis of differences in protein abundance between samples. However, considering the above mentioned intrinsic limitations of 2-D PAGE, other gel-based proteomic approaches, such as one-dimensional PAGE and Liquid Chromatography-Tandem Mass Spectrometry (GeLC-MS/MS) [36], can be combined with the 2-D PAGE/MS in order to mine deeper into a liposoluble proteome.

In this study, the membrane proteome of *M. agalactiae *was characterized by means of Triton X-114 fractionation, 2-D PAGE-MS, GeLC-MS/MS, and Gene Ontology classification. Differential expression of membrane proteins among *M. agalactiae *strains was also evaluated by 2D DIGE.

## Results

### Extraction of bacterial proteins and isolation of liposoluble proteins

**Figure 1:**
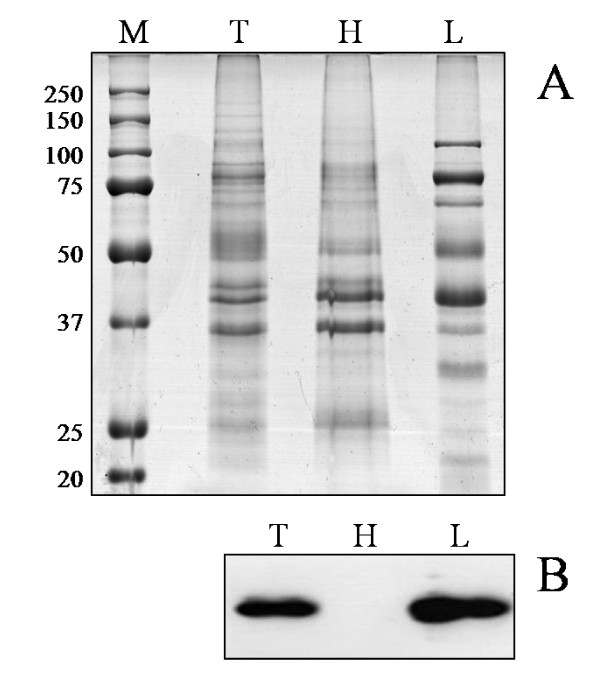
**Total protein patterns and Western immunoblotting reactivity of *M. agalactiae *PG2^T ^proteins. **Panel A. Coomassie blue staining. Panel B: Immunoblotting reactivity obtained with antibodies against the P48 lipoprotein. From left to right: M: molecular weight standards in kDa; T: total protein pattern; H: hydrosoluble protein fraction; L: liposoluble protein fraction obtained after Triton X-114 fractionation

This study was aimed to the systematic characterization of *M. agalactiae *PG2^T ^membrane proteins by means of a gel-based proteomic approach. In order to increase coverage for liposoluble proteins, a commercial fractionation method and the classical Triton X-114 fractionation protocol were applied to *M. agalactiae *PG2^T ^cell lysates. The best results were obtained by means of Triton X-114 fractionation. Figure 1A illustrates the hydrosoluble and liposoluble fractions obtained from *M. agalactiae *PG2^T^, flanked by the total protein pattern for comparison. The efficiency of the procedure in separating liposoluble proteins was evaluated by Western immunoblotting using a rabbit hyperimmune serum raised against *M. agalactiae *P48, a previously characterized surface lipoprotein [12,19]. As expected, presence of P48 was observed only in the total extract and in the Triton X-114 phase (Figure 1B), confirming that the fractionation method enabled separation and enrichment of hydrophobic proteins.

### 2-D PAGE/MS of *M. agalactiae *PG2^T ^liposoluble proteins

**Figure 2:**
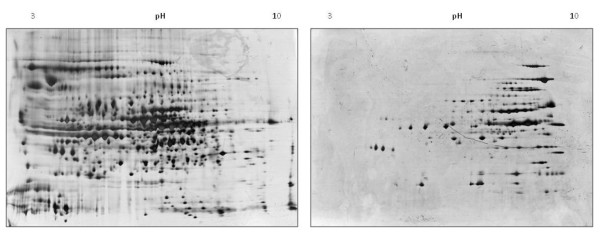
**2-D PAGE patterns of *M. agalactiae *PG2^T ^protein extracts**. Left: 2-D PAGE of a *M. agalactiae *PG2^T ^total protein extract. Right: 2-D PAGE of *M. agalactiae *PG2^T ^liposoluble proteins obtained after Triton X-114 fractionation.

Total proteins and the Triton X-114 soluble fraction of *M. agalactiae *PG2^T ^were subjected to 2-D PAGE separation in order to evaluate the extent of enrichment in basic and liposoluble proteins. As illustrated in Figure 2, left panel, a very high number of spots were present in the total protein map of *M. agalactiae *PG2^T ^but, as expected, basic proteins were poorly represented. Upon comparison, the 2-D PAGE map generated with the Triton X-114 soluble fraction showed a significant enrichment in basic proteins, with an excellent resolution also in high-abundance spots (Figure 2, right panel).

**Figure 3:**
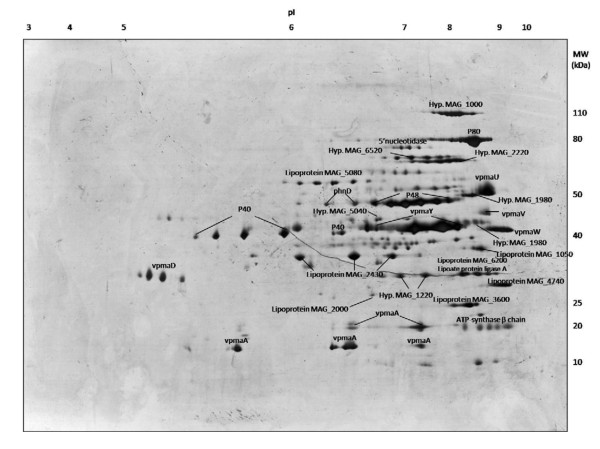
**2-D PAGE map of *M. agalactiae *PG2^T ^liposoluble proteins illustrating protein identifications obtained by mass spectrometry**. Proteins are indicated by grouping all individual identifications corresponding to the same protein in a series of spots.

In order to attain a systematic characterization of the liposoluble proteome, the Triton X-114 phase fraction of *M. agalactiae *PG2^T ^was subjected to 2-D PAGE under three different *pI *intervals: 3-10NL, 7-11, and 4-7 (Additional files 1, 2, and 3). From these 2D maps, about 300 spots were excised and identified by MALDI-TOF and nanoHPLC-nanoESI-Q-TOF MS. This approach led to the successful identification of 40 unique proteins, corresponding to 5.4% of all *M. agalactiae *PG2^T ^genes. Figure 3 reports a representative liposoluble protein map summarizing the main protein identifications accomplished on 2-D spots. A detailed description of all protein identifications is given in Additional file 4. Experimentally deduced molecular weight and *pI *of protein spots were compared with the theoretical parameters obtained from MASCOT, and most experimental data were in accordance with theoretical data. Few proteins, such as VpmA, were detected in multiple spots at different *pI*s and molecular weights, as expected for this class of lipoproteins which undergo size variation. The well-known immunogenic proteins [12,17,19-21] were all detected by 2-D PAGE at the expected *pI *and MW. All six variable surface lipoproteins encoded in the *M. agalactiae *PG2^T ^genome were also detected, some of which (such as VpmaY and VpmaD) with high expression levels, as could be expected considering their relevance in providing variability to the mycoplasmal antigenic mosaic.

### 2D DIGE of liposoluble proteins among the type strain and two field isolates of *M. agalactiae*

**Figure 4:**
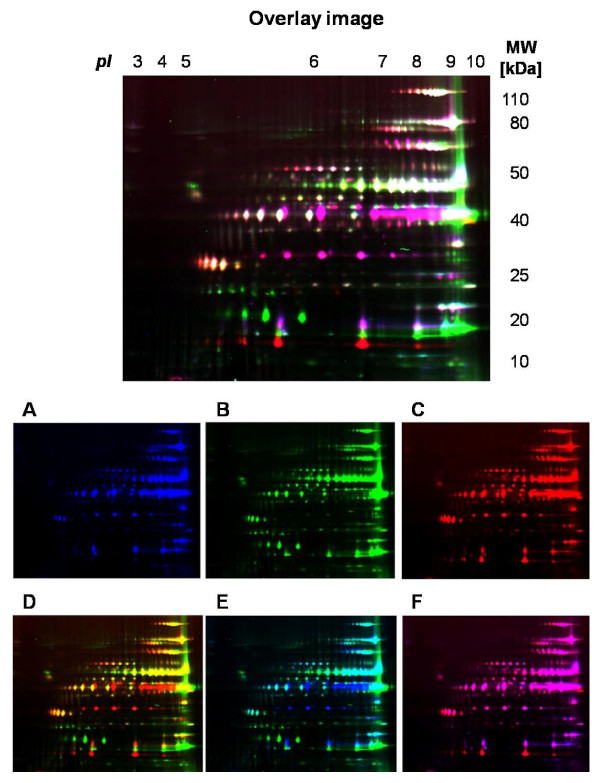
**2D DIGE of liposoluble proteins extracted from *M. agalactiae *PG2^T ^and two field strains**. Overlay image: image generated from the superimposition of the signals generated by the three samples. White indicates presence of the protein spot in all three isolates. Panels A, B, and C represent isolates PG2^T^, Nurri, and Bortigali, respectively. Panels D, E, and F represent the superimposition of Nurri/Bortigali, PG2^T^/Nurri, and PG2^T^/Bortigali, respectively.

In order to assess the suitability of 2-D PAGE for comparison of the membrane protein composition, the liposoluble protein profiles of *M. agalactiae *PG2^T ^and two field isolates were compared by 2D DIGE (Figure 4).

The images generated upon acquisition of the single color channels enable to evaluate the liposoluble protein profiles separately (Figure 4, A, B, C), while comparison of two protein profiles can be performed upon superimposition of two color signals (Figure 4, D, E, F). In the overlay image, the three proteome 2D maps can be compared. Although many spots are shared among the three profiles (in white), a number of differences in expression can be appreciated. In fact, several spots are present only in one (blue, green, red) or two profiles (purple, yellow, light blue). Many already known antigens (such as P80, P48, P40, and most Vpmas) appear in white, indicating superimposition of the three signals and therefore presence in all three bacterial proteomes. Several differences among the three profiles can be easily observed; for example, the series of spots at 40 kDa corresponding to VpmaY (in purple in the overlay image, Figure 4) is present only in two cases (PG2^T ^and Bortigali) while the series of spots at 23 kDa (in green) is present only in one case (Nurri). The application of this method to an adequate number of isolates might enable to easily detect constantly expressed proteins that might serve as candidate antigens for development of vaccines and diagnostic tools.

### GeLC-MS/MS of *M. agalactiae *PG2 liposoluble proteins

Although well suited for lipoprotein analysis, the 2-D PAGE/MS strategy presents drawbacks in analysis of transmembrane proteins, such as permeases or other highly hydrophobic proteins. Moreover, these protein classes may undergo selective loss during precipitation/resolubilization steps. In order to increase the membrane protein coverage and minimize selective protein loss, SDS-PAGE and GeLC-MS/MS analysis were performed on the non-precipitated Triton X-114 liposoluble protein fraction. A total of 36 slices were cut from the SDS-PAGE gel lane containing the separated liposoluble proteins (Additional file 5) and subjected to nanoHPLC-nanoESI-Q-TOF-MS/MS identification. Upon application of this method, 194 mycoplasma proteins were identified in total, corresponding to 26% of all *M. agalactiae *PG2^T ^genes, 38 of which were also identified by 2-D PAGE/MS (for a detailed list of protein identifications, see Additional file 6; Additional file 7 reports a summary table listing all unique protein identifications).

### Data analysis and classification

**Figure 5:**
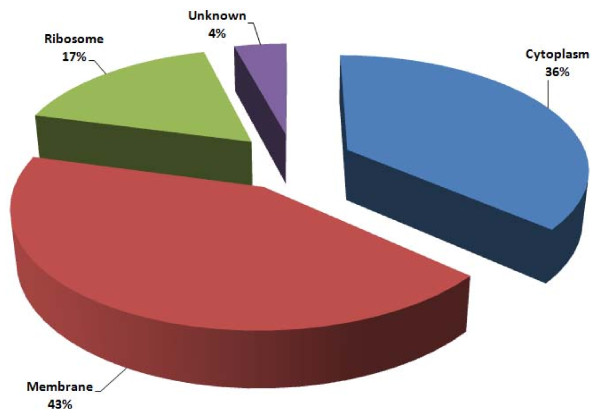
**GO graph of proteins identified by 2-D PAGE-MS and GeLC-MS/MS in the Triton X-114 fraction of *M. agalactiae *PG2^T^**. Protein identifications are classified according to cellular localization.

A gene ontology (GO) classification was carried out on proteins identified by 2-D PAGE/MS and GeLC-MS/MS. For the first method, proteins (n = 40) were mostly classified by the GO software as hypothetical lipoproteins (65%), cytoplasmic proteins (22%), ribosomal proteins (8%), and other membrane-located proteins (5%). When identifications obtained by GeLC-MS/MS were also included in the GO analysis (n = 194), 43% of all identifications were assigned to proteins located on the membrane, either lipoproteins (17%) or other membrane proteins (26%), whereas 36% were classified as cytoplasmic, 17% as ribosomal, and 4% of unknown localization (Figure 5).

**Figure 6:**
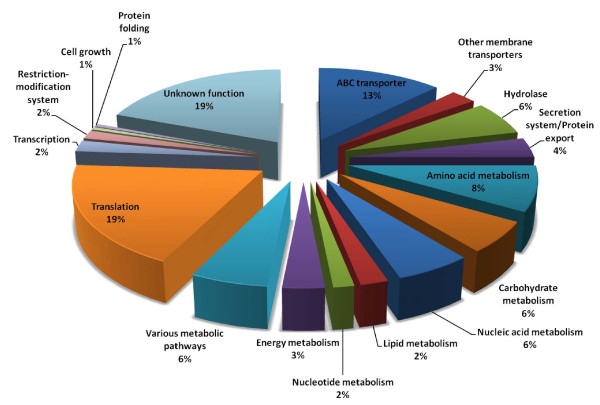
**GO graph of proteins identified by GeLC-MS/MS in the Triton X-114 fraction of *M. agalactiae *PG2^T^**. Protein identifications are classified according to function

All protein identifications were then classified according to function (Figure 6, and Additional file 7). As expected, a high proportion of the identified proteins perform membrane transport functions (about 16%), and belong mostly to ABC transporters (13%). Transmembrane proteins, such as permeases, were detected only by means of GeLC-MS/MS. Another highly represented functional process was translation (19%), due to the elevated number of ribosomal proteins identified. Hydrolytic enzymes were also significantly represented (6%), highlighting their crucial role for survival of mycoplasmas. Several other functional classes, such as enzymes involved in amino acid, carbohydrate, lipid, and nucleic acid metabolism, were significantly represented in the *M. agalactiae *PG2^T ^liposoluble protein fraction. Secretion/export systems accounted for 4% of all identified proteins; these components are in fact crucial for maturation and release of secreted proteins, but also for positioning/exposing lipoproteins on the outer side of the bacterial cell. About 19% of proteins could not be assigned a specific function by manual searches or GO classification.

Proteomic data were analyzed in order to investigate presence of liposoluble proteins resulting from expression of horizontally-transferred genes [24]. Among 194 identified proteins, 15 (7.8%) were acquired by HGT from the *Mycoplasma mycoides *cluster (Additional file 8), while 7 (3.7%) were acquired by HGT from other bacteria (Additional file 9), for a total of 22 proteins, making up to 11.5% of all expressed membrane proteins being derived from putative HGT events.

## Discussion

Gathering proteomic information on prokaryotic membranes is a challenging task, due to difficulties in cell fractionation and to the intrinsic chemical properties of membrane proteins in general. Therefore, both systematic and differential proteomic information on prokaryotic membranes is generally lacking. In this work, we approached the systematic characterization of what is believed to be one of the simplest bacterial pathogen membranes, in an attempt to move a step forward in our understanding of its composition, complexity, and function. In addition to its lower complexity, investigating membrane composition and plasticity in mycoplasmas is of particular interest since surface proteins are subjected to size and phase variation, and information on the extent and level of such variation is crucial in studies targeting identification of common immunogens, evaluation of immunological escape mechanisms, and adaptation of the bacterium to its host. All six variable surface lipoproteins encoded in the PG2^T ^genome [37] were detected by 2-D PAGE, although one of these (VpmaY) was not expressed in a field isolate examined by 2D DIGE. Triplicate experiments showed that the two-dimensional expression pattern of each field isolate is relatively stable under laboratory conditions, and that there is a reproducible differential expression of several protein spots in the field isolates compared to the type strain PG2. Interestingly, these differences are being detected in bacteria which were grown in culture media, where all protein variants should theoretically be expressed [37]. It was already demonstrated that the switching mechanism is so fast that it can be pointed out in a single colony on solid culture [14]. This might suggest that the lack of VpmaY in the isolate Nurri could result from a local genetic mutation. A large-scale study performed on a higher number of field isolates might enable the detection of constantly expressed proteins, which might be useful as targets for the development of vaccines and diagnostic tools for CA.

Mycoplasmas have evolved a parasitic lifestyle, and membrane transporters are consequently very important for uptake of nutrients and growth factors. The genome of *M. agalactiae *PG2^T ^encodes 18 ABC transporters, but proteins from only 10 of these were identified in this study. We also failed to identify all the components of a complete membrane transporter complex; however, it is possible that expression of all sequences encoded by the transporter gene operon may not necessarily take place at the same time. ABC transporters components encoded by different operons may likely interact to form functional transporters, producing the further advantage of creating many different combinations that can help evasion of host defense mechanisms. For instance, the genome of *M. agalactiae *PG2^T ^encodes for two oligopeptide (Opp) ABC transporters, one typical of the hominis group and one probably transferred by means of horizontal gene transfer mechanisms from *M. mycoides *subsp. *mycoides *and *M. capricolum *subsp. *capricolum*. We identified the substrate binding protein (OppA) from one operon, and the permease (OppC) and the ATP-binding protein (OppF) from another operon; notably, these proteins create a functional transporter. Moreover, OppA could be more than a simple substrate binding protein, since it was demonstrated to play an important role in pathogenicity in *M. hominis *by inducing ATP release and cell death of HeLa cells in vitro and by mediating adhesion to host cells [38-40]. Other authors reported a different pattern of expression of these operons: in the study by Nouvel and co-workers [37], only OppA, OppF, and OppD were detected. These apparently controversial results could be due to technical issues, or be dependent on variations in expression of Opps within the PG2^T ^strain. This will need to be elucidated in future studies.

Upon analysis of all MS data, the proteins putatively assigned by the GO software as cytoplasmic accounted to 36%. Among these, many hydrolases were present. However, lipases, peptidases, and nucleases might be associated to the membrane compartment and assist in reducing macromolecules to simple components, enabling their uptake. In fact, mycoplasmas lack many biosynthetic pathways and rely on internalization of nucleotides, amino acids, sugars and lipids from their external environment. Recently, it was reported that hydrolytic enzymes are surface-located in mycoplasmas, and that they can be associated with ABC transporters in order to digest macromolecules before uptake of simpler components, or play major roles in pathogenicity [41]. Interestingly, in the *M. agalactiae *genome, the genes coding for many of these hydrolases are also located close to ABC transporter operons.

Several other proteins have a predicted cytoplasmic localization, but could be membrane-associated in mycoplasmas, such as the elongation factor tu (EF-Tu) and the E1 beta subunit of the pyruvate dehydrogenase complex. Traditionally, these are considered to be cytoplasmic proteins involved in protein synthesis and energy production, respectively, but it was demonstrated that in *M. pneumoniae *they are surface exposed and interact with host fibronectin, mediating adhesion [42,43]. It was also demonstrated that many "cytoplasmic" proteins such as the EF-Tu are strong antigens in many mycoplasma species [22,44,45].

Ribosomal proteins represent a significant proportion of the mycoplasma liposoluble proteome. This might appear inconsistent, but in spite of their traditionally cytoplasmic localization, it was already demonstrated that ribosomes interact with the bacterial protein export complex [46]. Moreover, it is well known that in eukaryotes ribosomes are associated with endoplasmic reticulum, where they participate in the protein secretion pathway [47]. Several proteins that take part in other metabolic pathways were also identified in the liposoluble fraction of *M. agalactiae *PG2^T^. We could speculate that many proteins involved in nutrient metabolism might associate with proteins devoted to internalization of precursors in metabolizing complexes, and be co-purified with these. Nonetheless, a pre-fractionation of membranes was not performed because of inherent technical difficulties, and we cannot rule out that enzymes with high hydrophobicity might be present as cytoplasmic contaminants.

The recent work by Sirand-Pugnet and coworkers revealed the occurrence of horizontal gene transfer (HGT) events in *M. agalactiae*. The expression of proteins acquired by HGT highlights the importance of horizontal gene flow for the evolutionary plasticity of mycoplasmas; for instance, by allowing changes in host and/or tissue tropism through acquisition of traits enabling colonization and survival in new niches [24,48]. In total, an impressing 11.7% of proteins expressed on the *M. agalactiae *membrane are coming from other bacteria, reinforcing the view that an important part in the evolution of mycoplasmas might be driven by genetic exchange with bacteria sharing the same host districts, probably in order to compensate the concurrent process of gene loss [24]. Another interesting observation was the detection of MAG_2340, a hypothetical lipoprotein which is apparently the result of an horizontal gene transfer event with mycoplasmas of the mycoides cluster (Additional file 8), which was not detected by Nouvel et al. in the PG2^T ^liposoluble proteome [37].

Hypothetical proteins were of particular interest; since these did not have an assigned function, similarity searches were conducted with BLAST tools in order to infer their possible role in the biology of mycoplasmas. Among these, the hypothetical lipoprotein MAG_1670 belongs to the mycoides cluster LppA/P72 family, and it is an antigen recognized early and persistently in infection [49]. The hypothetical protein MAG_0250 has an indigoidine synthase A (IdgA)-like domain similar to *Clostridium *spp. IdgA is involved in the biosynthesis of indigoidine, a blue pigment synthesized by *Erwinia chrysanthemi *and implicated in pathogenicity and protection from oxidative stress by scavenging oxygen radicals [50]. Indigoidine production increases tolerance to oxidative stress and contributes to aggressiveness, and might therefore act as a virulence factor.

## Conclusions

2-D PAGE studies might be extremely powerful for comparison of protein expression in different mycoplasma isolates, especially when considering that lipoproteins can be selectively detected with this method, and that size and phase variations can be easily spotted through the application of powerful differential comparison approaches as the 2D DIGE. However, these need to be integrated with traditional Western immunoblotting and GeLC-MS/MS for a deeper coverage and characterization of other mycoplasmal surface immunogens to be used as tools for vaccination, diagnosis, and therapy. This combined approach allowed the identification and characterization of 194 *M. agalactiae *proteins putatively localized on the membrane or associated to it, providing useful insights on its composition. In the future, alternative approaches such as blue native electrophoresis and chemical crosslinking of surface proteins will also enable to elucidate functional and structural aspects of membrane proteins that cannot be accounted for by the traditional gel-based proteomic approaches.

## Methods

### Bacterial strains and culture conditions

At least three replicate cultures of *Mycoplasma agalactiae *PG2^T ^and two Sardinian field isolates (named Bortigali and Nurri), were grown in PPLO medium supplemented with 20% heat inactivated horse serum and 500 μg/mL ampicillin, at 37°C with constant agitation. Mycoplasmas were collected by centrifugation (10 min at 10,000 × g at 4°C), and washed three times with PBS. At least three mycoplasma pellets were obtained from each bacterial culture replicate, and used for genetic and proteomic analyses. Total DNA was extracted from a set of pellets with DNeasy Blood & Tissue Kit (Qiagen), and subjected to FS1-FS2 PCR for species confirmation [51].

### Total protein extracts and Triton X-114 fractionation

For total protein extracts, bacterial pellets were resuspended in 1% hot SDS, incubated for 3 minutes at 95°C, chilled, and diluted with lysis buffer (7 M urea, 2 M thiourea, 2.5% CHAPS, 2% ASB-14, 40 mM Tris-HCl pH 8.8, 1% IPG-buffer, protease inhibitors), and insoluble materials were discarded by centrifugation (10 min at 10,000 × g at 4°C) [52]. Hydrophilic and hydrophobic protein fractions were obtained by Triton X-114 fractionation [29,30] and ProteoPrep^® ^Membrane Extraction Kit (Sigma-Aldrich). Proteins samples were quantified as described [52].

### SDS-PAGE and 2-D PAGE

SDS-PAGE was performed on 8% polyacrylamide gels on a Protean Tetra Cell (Bio-Rad) following the manufacturer instructions, and gels were stained with PageBlue™ Protein Staining Solution (Fermentas).

Prior to 2-D PAGE, Triton X-114 fractions were precipitated with methanol-chloroform [35] and resuspended in lysis buffer (8 M urea, 2 M thiourea, 2.5% CHAPS, 2% ASB-14, 40 mM Tris-HCl pH 8.8, 1% IPG-buffer, protease inhibitors). Resuspended proteins (150 μg) were then absorbed overnight into 18 cm IPG strips (GE Healthcare, pH 3-10 NL, pH 7-11, and pH 4-7). Strips were focused on an IPGphor (GE Healthcare) for a total of 60,000 Vh. After focusing, strips were equilibrated in 50 mM Tris-HCl, pH 6.8, 2% SDS, 7 M urea, 10% glycerol, supplemented with 2% DTT for 15 min, and then with 2.5% iodoacetamide for 15 min. The second dimension (SDS-PAGE) was conducted on 10% to 18% polyacrylamide gradient gels, on an Ettan DALTsix electrophoresis system (GE Healthcare), following manufacturer's instructions. 2-D gels were silver stained with a mass-compatible method [53] and images were digitalized with an Image Scanner (GE Healthcare).

### 2D DIGE

For 2D DIGE analysis, the two field isolates Bortigali and Nurri were compared to PG2^T^. Triton X-114 Protein extracts were precipitated and resuspended in lysis buffer as described above. Then, samples were labeled with CyDye DIGE Fluors (GE Healthcare) according to the minimal labeling protocol provided by the manufacturer. Briefly, after CyDye reconstitution with dimethylformamide (DMF) and preparation of a working solution (200 pmol/μL), 1 μL of diluted CyDye was added to a volume of protein sample equivalent to 50 μg. Samples were left on ice for 30 minutes in the dark, and then 1 μL of 10 mM lysine was added to stop the reaction. Labeled samples were mixed, IPG buffer corresponding to the desired pH range was added at a 1% final concentration, and DeStreak Rehydration Solution (GE Healthcare) was added to a total volume of 340 μL. 18 cm IPG strips (GE Healthcare) were passively rehydrated for at least 6 hours. IEF was carried out on an Ettan IPGphor II (GE Healthcare) for a total of ~60,000 Vh. After focusing, strips were equilibrated in 50 mM Tris-HCl, pH 6.8, 2% SDS, 7 M urea, 10% glycerol, supplemented with 2% DTT for 10 min, and then with 2.5% iodoacetamide for 10 min. Proteins were then subjected to SDS-PAGE in 10-18% gradient polyacrylamide gels on the Ettan DALTsix system (GE Healthcare), following the manufacturer instructions. DIGE images were detected with a Typhoon Scanner (GE Healthcare) and processed with DeCyder (GE Healthcare) for image analysis.

### Western Immunoblotting

SDS-PAGE resolved proteins were transferred onto nitrocellulose membranes with a Mini-Trans-Blot Cell (Bio-Rad) at 250 mA for one hour. After blotting, membranes were blocked with PBS-0.05% Tween 20 (PBS-T) containing 3% BSA. Membranes were incubated for one hour with a rabbit hyperimmune serum raised against *M. agalactiae *recombinant P48 (*M. agalactiae *rP48) [12]. Membranes were washed five times with PBS-T and incubated with the appropriate HRP-conjugated secondary antibodies (Sigma). After five washes, membranes were developed with SuperSignal West Pico Chemiluminescent Substrate (Pierce) and images were acquired with a VersaDoc MP 4000 Imaging System (Bio-Rad).

### Spot picking and *in situ *tryptic digestion

Protein spots obtained upon 2-D PAGE separation of the Triton X-114 extract from the strain PG2^T ^were manually excised from gels, destained with 15 mM K_3_Fe(CN)_6 _in 50 mM Na_2_S_2_O_3 _and stored in acetonitrile. Spots were then subjected to an O/N tryptic digestion at 37°C in 50 mM (NH_4_)HCO_3_, pH 8.0, using 40 to 80 ng of trypsin depending on spot intensity. Peptide mixtures were collected by elution with acetonitrile followed by centrifugation. Peptides were then acidified with TFA 20%, dried in SpeedVac^®^, resuspended in 0.2% formic acid and stored at -20°C.

### GeLC-MS/MS

The Triton X-114 fraction was diluted with 4× Laemmli buffer [54], 20 μg of proteins were loaded in an 8% polyacrylamide gel, and SDS-PAGE was performed as previously described. After gel staining, bands were manually excised, destained, reduced, alkylated, and finally subjected to *in situ *tryptic digestion as previously described [55]. Peptide mixtures were identified by nanoHPLC-nanoESI-Q-TOF-analysis. One-dimensional patterns were analyzed with Quantity One software (Bio-Rad).

### MALDI-MS

Mass spectra were recorded on a MALDI micro (Waters, Manchester, UK) equipped with a reflectron analyzer and used in delayed extraction mode, as described previously [56]. Peptide samples were mixed with an equal volume of α-cyano-4-hydroxycynnamic acid as matrix (10 mg/mL in acetonitrile/0.2% TFA) (70:30, v/v), applied to the metallic sample plate, and air dried. Mass calibration was performed by using the standard mixture provided by manufacturer. Raw data, reported as monoisotopic masses, were then introduced into the in-house Mascot Peptide Mass Fingerprinting software (Version 2.2, Matrix Science, Boston, MA), and used for protein identification. Search parameters were as follows: fixed modifications carbamidomethyl (C), variable modifications pyro-Glu (N-term Q) and oxidation (M), peptide tolerance 80 ppm, enzyme trypsin, allowing up to 2 missed cleavages.

### LC-MS/MS

LC-MS/MS analyses of tryptic digests were performed on a Q-TOF hybrid mass spectrometer equipped with a nano lock Z-spray source, and coupled on-line with a capillary chromatography system CapLC (Waters, Manchester, UK), as described previously [55]. After loading, the peptide mixture was first concentrated and washed at 20 μL/min onto a reverse-phase pre-column (Symmetry 300, C18, 5 μm, NanoEase, Waters) using 0.2% formic acid as eluent. The sample was then fractionated onto a C18 reverse-phase capillary column (Nanoflow column 5 μm Biosphere C18, 75 μm × 200 mm, Nanoseparations) at a flow rate of 250 nL/min, using a linear gradient of eluent B (0.2% formic acid in 95% acetonitrile) in A (0.2% formic acid in 5% acetonitrile) from 2 to 40% in 27 min. The mass spectrometer was set up in a data-dependent MS/MS mode where a full scan spectrum (m/z acquisition range from 400 to 1600 Da/e) was followed by tandem mass spectra (m/z acquisition range from 100 to 2000 Da/e). Peptide ions were selected as the three most intense peaks of the previous scan. A suitable collision energy was applied depending on the mass and charge of the precursor ion. Argon was used as the collision gas. Mass calibration was conducted on the Glu-fibrino peptide B (Sigma) fragmentation pattern. ProteinLynx software (Version 2.2.5), provided by the manufacturers, was used to analyze raw MS and MS/MS spectra and to generate a peak list which was introduced in the in-house Mascot MS/MS ion search software (Version 2.2, Matrix Science, Boston, MA) for protein identification. NCBI was used as sequence database. Search parameters were as follows: fixed modifications carbamidomethyl (C), variable modifications pyro-Glu (N-term Q) and oxidation (M), peptide tolerance 30 ppm, MS/MS tolerance 0.3 Da, charge state +2 and +3, enzyme trypsin, allowing up to 1 missed cleavage.

### Data analysis

MS data were subjected to gene ontology analysis with Blast2GO, using default parameters [57]. Identified proteins were divided into classes for functional and localization analysis; data produced by the software were used for generation of graphs by Microsoft Excel.

## Authors' contributions

CC and MFA performed the experimental design, carried out the protein fractionation and electrophoresis, performed data analysis, and drafted the manuscript. DP carried out the mass spectrometry identifications. BC participated in the design of the study. EC and LC performed animal diagnosis, collection of animal samples, isolation, molecular identification, and cultivation of mycoplasmas. SU contributed to coordination of the study and data interpretation, and helped to draft the manuscript. AA and MP conceived the study, participated in its design and coordination, and helped to draft the manuscript. All authors read and approved the final manuscript.

## Supplementary Material

Additional file 1 **2-D PAGE map of liposoluble proteins from *M. agalactiae *PG2^T ^illustrating the protein identifications obtained by MS on the 3-10NL *pI *Interval**. Click here for file

Additional file 2 **2-D PAGE map of liposoluble proteins from *M. agalactiae *PG2^T ^illustrating the protein identifications obtained by MS on the 7-11 *pI *Interval**. Click here for file

Additional file 3 **2-D PAGE map of liposoluble proteins from *M. agalactiae *PG2^T ^illustrating the protein identifications obtained by MS on the 4-7 *pI *Interval**. Click here for file

Additional file 4 **Table listing all protein identifications obtained from 2-D PAGE maps**. The proteins listed in this table were identified from 2-D PAGE maps of the *M. agalactiae *PG2^T ^Triton X-114 fraction. Maps are represented in Additional files 1 (pH 3-10NL), 2 (pH 7-11) and 3 (pH 4-7). Click here for file

Additional file 5 **Protein profile of liposoluble proteins before and after precipitation. Right: approach used for GeLC-MS/MS characterization**. The bars indicate the regions cut from the PAGE gel and subjected to mass spectrometry characterization. Protein identifications are reported in additional file 6, from top to bottom. Click here for file

Additional file 6 **Table listing all protein identifications obtained by GeLC-MS/MS of the *M. agalactiae *PG2^T ^Triton X-114 liposoluble fraction**. The protein profile used and the number of slices are reported in Additional file 5. Click here for file

Additional file 7 **Functional analysis, number of peptide hits, and method of detection of *M. agalactiae *PG2^T ^liposoluble proteins**. The results of 2D DIGE with the two field strains Nurri and Bortigali are also reported (TPH, total peptide hits; NA, not applicable). Click here for file

Additional file 8 **Proteins identified in the *M. agalactiae *proteome potentially resulting from Horizontal Gene Transfer events with *M. mycoides *subsp. *mycoides *and *M. capricolum *subsp. *capricolum***. Click here for file

Additional file 9 **Proteins identified in the *M. agalactiae *proteome potentially resulting from Horizontal Gene Transfer events with other bacteria**. Click here for file
