# Supplementary material for: The liposoluble proteome of Mycoplasma agalactiae: an insight into the minimal protein complement of a bacterial membrane
Source: BMC Microbiol. 2010 Aug 25;10:225. doi: 10.1186/1471-2180-10-225 (PMC2941501; doi:10.1186/1471-2180-10-225)

**Additional file 2: 2-D PAGE map of liposoluble proteins from *M. agalactiae* PG2^T^ illustrating the protein identifications obtained by MS on the 7-11 *pI* Interval.**


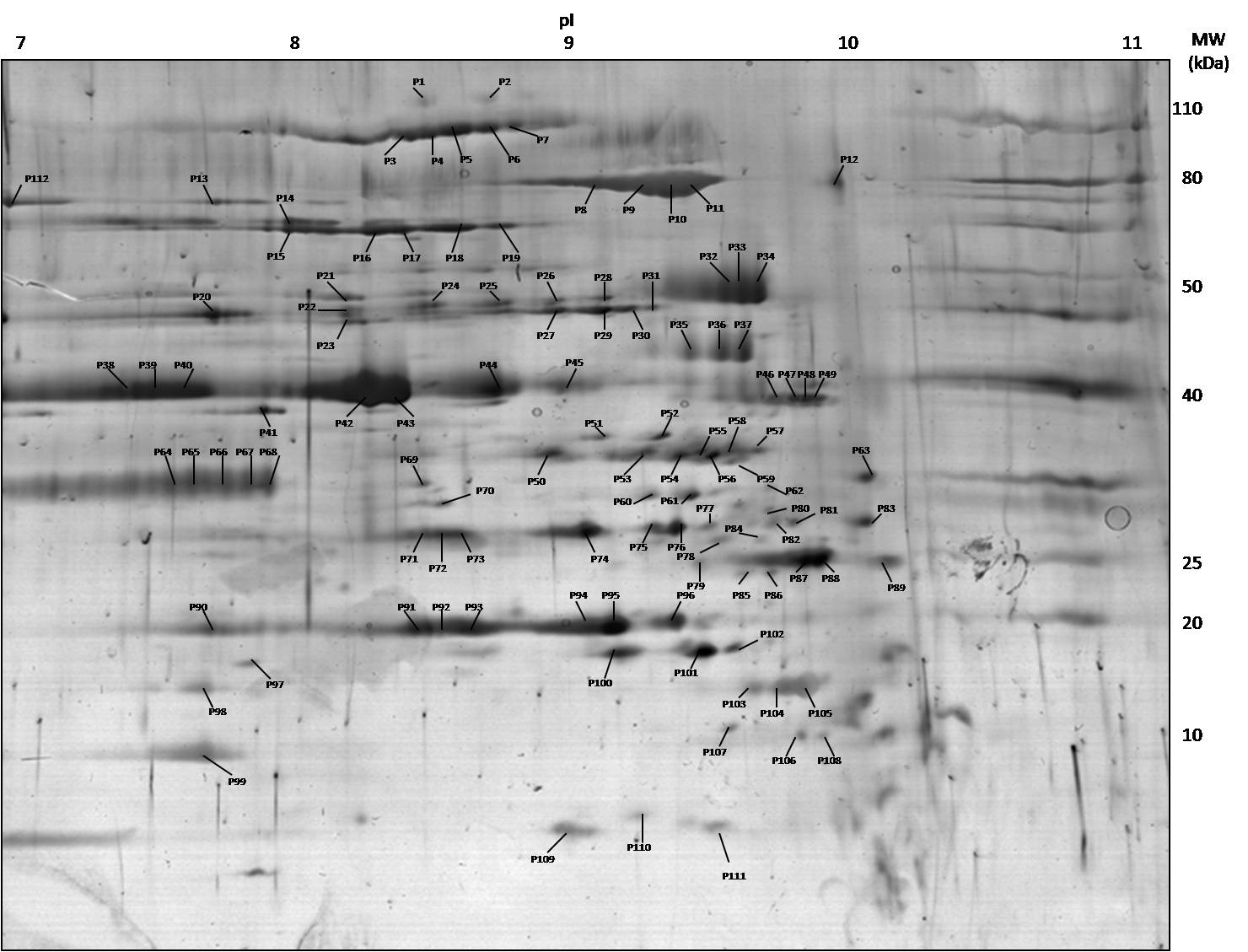

Supplement: Additional file 2 — 2-D PAGE map of liposoluble proteins from M. agalactiae PG2T illustrating the protein identifications obtained by MS on the 7-11 pI Interval. [file 1471-2180-10-225-S2.DOC]
