# Supplementary material for: The liposoluble proteome of Mycoplasma agalactiae: an insight into the minimal protein complement of a bacterial membrane
Source: BMC Microbiol. 2010 Aug 25;10:225. doi: 10.1186/1471-2180-10-225 (PMC2941501; doi:10.1186/1471-2180-10-225)
Supplement: Additional file 4 — Table listing all protein identifications obtained from 2-D PAGE maps. The proteins listed in this table were identified from 2-D PAGE maps of the M. agalactiae PG2T Triton X-114 fraction. Maps are represented in Additional files 1 (pH 3-10NL), 2 (pH 7-11) and 3 (pH 4-7). [file 1471-2180-10-225-S4.DOC]

### Additional file 4: Table listing all protein identifications obtained from 2-D PAGE maps. The proteins listed in this table were identified from 2-D PAGE maps of the *M. agalactiae* PG2^T^Triton X-114 fraction . Maps are represented in Additional files 1 (pH 3-10NL), 2 (pH 7-11), and 3 (pH 4-7).

| **Spot** | **Map** | **Protein** | **Organism** | **Mw (Da)** | **pI** | **Score** | **Instrument** | **Queries** | **Coverage** | **Acc. No.** | **Locus tag** |
| --- | --- | --- | --- | --- | --- | --- | --- | --- | --- | --- | --- |
| PG1 | 1 | NI |  |  |  |  |  |  |  |  |  |
| PG2 | 1 | NI |  |  |  |  |  |  |  |  |  |
| PG3 | 1 | Hypotetical protein MAG_1000 | *M. agalactiae* PG2 | 109727 | 8.66 | 155 | MALDI-TOF | 21 | 25% | gi|148377368 | MAG_1000 |
| PG4 | 1 | Hypotetical protein MAG_1000 | *M. agalactiae* PG2 | 109727 | 8.66 | 71 | MALDI-TOF | 10 | 18% | gi|148377368 | MAG_1000 |
| PG5 | 1 | Hypotetical protein MAG_1000 | *M. agalactiae* PG2 | 109727 | 8.66 | 175 | MALDI-TOF | 24 | 25% | gi|148377368 | MAG_1000 |
| PG6 | 1 | Hypotetical protein MAG_1000 | *M. agalactiae* PG2 | 109727 | 8.66 | 199 | MALDI-TOF | 23 | 25% | gi|148377368 | MAG_1000 |
| PG7 | 1 | Hypotetical protein MAG_1000 | *M. agalactiae* PG2 | 109727 | 8.66 | 91 | MALDI-TOF | 13 | 11% | gi|148377368 | MAG_1000 |
| PG8 | 1 | Hypotetical protein MAG_1000 | *M. agalactiae* PG2 | 109727 | 8.66 | 168 | MALDI-TOF | 20 | 23% | gi|148377368 | MAG_1000 |
| PG9 | 1 | P80. lipoprotein | *M. agalactiae* PG2 | 81102 | 9.08 | 200 | MALDI-TOF | 24 | 37% | gi|148377765 | MAG_5030 |
| PG9bis | 1 | P80. lipoprotein | *M. agalactiae* PG2 | 81102 | 9.08 | 74 | ESI Q-TOF | 5 | 6% | gi|148377765 | MAG_5030 |
| PG10 | 1 | P80. lipoprotein | *M. agalactiae* PG2 | 81102 | 9.08 | 136 | ESI Q-TOF | 10 | 11% | gi|148377765 | MAG_5030 |
| PG11 | 1 | P80. lipoprotein | *M. agalactiae* PG2 | 81102 | 9.08 | 181 | MALDI-TOF | 23 | 35% | gi|148377765 | MAG_5030 |
| PG12 | 1 | 5'nucleotidase | *M. agalactiae* PG2 | 76195 | 8.36 | 72 | MALDI-TOF | 7 | 15% | gi|148377854 | MAG_5910 |
| PG13 | 1 | 5'nucleotidase | *M. agalactiae* PG2 | 76195 | 8.36 | 96 | MALDI-TOF | 10 | 17% | gi|148377854 | MAG_5910 |
| PG13bis | 1 | 5'nucleotidase | *M. agalactiae* PG2 | 76195 | 8.36 | 113 | MALDI-TOF | 10 | 18% | gi|148377854 | MAG_5910 |
| PG14 | 1 | Hypothetical protein MAG_6520 | *M. agalactiae* PG2 | 70087 | 8.58 | 64 | MALDI-TOF | 8 | 14% | gi|148377915 | MAG_6520 |
| PG14bis | 1 | Hypothetical protein MAG_6520 | *M. agalactiae* PG2 | 70087 | 8.58 | 59 | MALDI-TOF | 6 | 15% | gi|148377915 | MAG_6520 |
| PG15 | 1 | NI |  |  |  |  |  |  |  |  |  |
| PG16 | 1 | Hypothetical protein MAG_2220 | *M. agalactiae* PG2 | 69573 | 8.78 | 73 | MALDI-TOF | 8 | 17% | gi|148377488 | MAG_2220 |
| PG17 | 1 | Hypothetical protein MAG_2220 | *M. agalactiae* PG2 | 69573 | 8.78 | 141 | MALDI-TOF | 16 | 28% | gi|148377488 | MAG_2220 |
| PG18 | 1 | Hypothetical protein MAG_2220 | *M. agalactiae* PG2 | 69573 | 8.78 | 149 | MALDI-TOF | 13 | 33% | gi|148377488 | MAG_2220 |
| PG19 | 1 | Hypothetical protein MAG_2220 | *M. agalactiae* PG2 | 69573 | 8.78 | 110 | MALDI-TOF | 12 | 28% | gi|148377488 | MAG_2220 |
| PG20 | 1 | Hypothetical protein MAG_6520 | *M. agalactiae* PG2 | 70087 | 8.58 | 64 | MALDI-TOF | 8 | 18% | gi|148377915 | MAG_2220 |
| PG21 | 1 | NI |  |  |  |  |  |  |  |  |  |
| PG22 | 1 | NI |  |  |  |  |  |  |  |  |  |
| PG23 | 1 | Hypothetical protein MYPU_0730 | Mycoplasma pulmonis UAB CTIP | 26393 | 7.77 | 28 | ESI Q-TOF | 1 | 4% | gi|15828544 |  |
| PG24 | 1 | NI |  |  |  |  |  |  |  |  |  |
| PG25 | 1 | NI |  |  |  |  |  |  |  |  |  |
| PG26 | 1 | NI |  |  |  |  |  |  |  |  |  |
| PG27 | 1 | Lipoprotein, MAG_5080 | *M. agalactiae* PG2 | 59415 | 6.77 | 94 | MALDI-TOF | 10 | 20% | gi|148377770 | MAG_5080 |
| PG28 | 1 | Lipoprotein, MAG_5080 | *M. agalactiae* PG2 | 59415 | 6.77 | 65 | MALDI-TOF | 7 | 18% | gi|148377770 | MAG_5080 |
| PG29 | 1 | Lipoprotein, MAG_5080 | *M. agalactiae* PG2 | 59415 | 6.77 | 63 | MALDI-TOF | 8 | 21% | gi|148377770 | MAG_5080 |
| PG30 | 1 | NI |  |  |  |  |  |  |  |  |  |
| PG31 | 1 | NI |  |  |  |  |  |  |  |  |  |
| PG32 | 1 | NI |  |  |  |  |  |  |  |  |  |
| PG33 | 1 | NI |  |  |  |  |  |  |  |  |  |
| PG34 | 1 | Variable surface lipoprotein V | *M. agalactiae* PG2 | 37362 | 9.25 | 56 | ESI Q-TOF | 2 | 5% | gi|148377967 | MAG_7050 |
|  |  | Variable surface lipoprotein U | *M. agalactiae* PG2 | 46993 | 9.26 | 56 | ESI Q-TOF | 2 | 4% | gi|32189693 | MAG_7090 |
| PG35 | 1 | Variable surface lipoprotein V | *M. agalactiae* PG2 | 37362 | 9.25 | 108 | ESI Q-TOF | 7 | 14% | gi|148377967 | MAG_7050 |
|  |  | Variable surface lipoprotein U | *M. agalactiae* PG2 | 46993 | 9.26 | 108 | ESI Q-TOF | 7 | 11% | gi|32189693 | MAG_7090 |
| PG36 | 1 | Variable surface lipoprotein V | *M. agalactiae* PG2 | 37362 | 9.25 | 42 | ESI Q-TOF | 1 | 2% | gi|148377967 | MAG_7050 |
|  |  | Variable surface lipoprotein U | *M. agalactiae* PG2 | 46993 | 9.26 | 42 | ESI Q-TOF | 1 | 2% | gi|32189693 | MAG_7090 |
| PG37 | 1 | Lipoprotein, MAG_1980 | *M. agalactiae* PG2 | 53788 | 8.98 | 69 | MALDI-TOF | 7 | 15% | gi|148377464 | MAG_1980 |
| PG38 | 1 | Lipoprotein, MAG_1980 | *M. agalactiae* PG2 | 53788 | 8.98 | 88 | MALDI-TOF | 9 | 19% | gi|148377464 | MAG_1980 |
| PG39 | 1 | Alkylphosphonate ABC transporter substrate-binding protein | *M. agalactiae* PG2 | 49744 | 6.99 | 95 | MALDI-TOF | 9 | 22% | gi|148377535 | MAG_2690 |
| PG40 | 1 | Alkylphosphonate ABC transporter substrate-binding protein | *M. agalactiae* PG2 | 49744 | 6.99 | 73 | MALDI-TOF | 7 | 17% | gi|148377535 | MAG_2690 |
| PG41 | 1 | P48. lipoprotein | *M. agalactiae* PG2 | 51232 | 8.39 | 62 | MALDI-TOF | 5 | 17% | gi|148377280 | MAG_0120 |
| PG42 | 1 | P48. lipoprotein | *M. agalactiae* PG2 | 51232 | 8.39 | 114 | MALDI-TOF | 10 | 25% | gi|148377280 | MAG_0120 |
| PG43 | 1 | P48. lipoprotein | *M. agalactiae* PG2 | 51232 | 8.39 | 125 | MALDI-TOF | 12 | 33% | gi|148377280 | MAG_0120 |
| PG44 | 1 | P48. lipoprotein | *M. agalactiae* PG2 | 51232 | 8.39 | 137 | MALDI-TOF | 11 | 33% | gi|148377280 | MAG_0120 |
| PG45 | 1 | P48. lipoprotein | *M. agalactiae* PG2 | 51232 | 8.39 | 98 | MALDI-TOF | 9 | 25% | gi|148377280 | MAG_0120 |
| PG46 | 1 | P48. lipoprotein | *M. agalactiae* PG2 | 51232 | 8.39 | 81 | MALDI-TOF | 7 | 19% | gi|148377280 | MAG_0120 |
| PG47 | 1 | Variable surface lipoprotein V | *M. agalactiae* PG2 | 37362 | 9.25 | 40 | ESI Q-TOF | 1 | 2% | gi|148377967 | MAG_7050 |
|  |  | Variable surface lipoprotein U | *M. agalactiae* PG2 | 46993 | 9.26 | 40 | ESI Q-TOF | 1 | 2% | gi|32189693 | MAG_7090 |
| PG48 | 1 | NI |  |  |  |  |  |  |  |  |  |
| PG49 | 1 | Hypothetical protein MAG_5040 | *M. agalactiae* PG2 | 44828 | 8.46 | 96 | MALDI-TOF | 8 | 26% | gi|148377766 | MAG_5040 |
| PG50 | 1 | Hypothetical protein MAG_5040 | *M. agalactiae* PG2 | 44828 | 8.46 | 104 | MALDI-TOF | 8 | 30% | gi|148377766 | MAG_5040 |
| PG51 | 1 | Variable surface lipoprotein Y | *M. agalactiae* PG2 | 37528 | 8.81 | 81 | MALDI-TOF | 7 | 30% | gi|148377970 | MAG_7080 |
| PG52 | 1 | Variable surface lipoprotein Y | *M. agalactiae* PG2 | 37528 | 8.81 | 88 | MALDI-TOF | 7 | 25% | gi|148377970 | MAG_7080 |
| PG53 | 1 | Variable surface lipoprotein Y | *M. agalactiae* PG2 | 37528 | 8.81 | 55 | MALDI-TOF | 5 | 24% | gi|148377970 | MAG_7080 |
| PG54 | 1 | Variable surface lipoprotein Y | *M. agalactiae* PG2 | 37528 | 8.81 | 55 | MALDI-TOF | 5 | 24% | gi|148377970 | MAG_7080 |
| PG55 | 1 | Variable surface lipoprotein Y | *M. agalactiae* PG2 | 37528 | 8.81 | 72 | MALDI-TOF | 7 | 28% | gi|148377970 | MAG_7080 |
| PG56 | 1 | Variable surface lipoprotein Y | *M. agalactiae* PG2 | 37528 | 8.81 | 69 | MALDI-TOF | 7 | 31% | gi|148377970 | MAG_7080 |
| PG57 | 1 | Variable surface lipoprotein Y | *M. agalactiae* PG2 | 37528 | 8.81 | 56 | MALDI-TOF | 5 | 24% | gi|148377970 | MAG_7080 |
| PG58 | 1 | Variable surface lipoprotein W | *M. agalactiae* PG2 | 35472 | 9.54 | 127 | MALDI-TOF | 10 | 30% | gi|148377968 | MAG_7060 |
| PG59 | 1 | Variable surface lipoprotein W | *M. agalactiae* PG2 | 35472 | 9.54 | 87 | MALDI-TOF | 9 | 27% | gi|148377968 | MAG_7060 |
| PG60 | 1 | Variable surface lipoprotein W | *M. agalactiae* PG2 | 35472 | 9.54 | 135 | MALDI-TOF | 11 | 31% | gi|148377968 | MAG_7060 |
| PG61 | 1 | P40, lipoprotein | *M. agalactiae* PG2 | 39951 | 8.19 | 40 | ESI Q-TOF | 2 | 5% | gi|148377507 | MAG_2410 |
| PG62 | 1 | P40, lipoprotein | *M. agalactiae* PG2 | 39951 | 8.19 | 80 | MALDI-TOF | 6 | 21% | gi|148377507 | MAG_2410 |
| PG63 | 1 | P40, lipoprotein | *M. agalactiae* PG2 | 39951 | 8.19 | 80 | MALDI-TOF | 6 | 21% | gi|148377507 | MAG_2410 |
| PG64 | 1 | P40, lipoprotein | *M. agalactiae* PG2 | 39951 | 8.19 | 118 | MALDI-TOF | 9 | 28% | gi|148377507 | MAG_2410 |
| PG65 | 1 | NI |  |  |  |  |  |  |  |  |  |
| PG66 | 1 | P40, lipoprotein | *M. agalactiae* PG2 | 39951 | 8.19 | 127 | ESI Q-TOF | 8 | 16% | gi|148377507 | MAG_2410 |
| PG67 | 1 | P40, lipoprotein | *M. agalactiae* PG2 | 39951 | 8.19 | 58 | MALDI-TOF | 6 | 19% | gi|148377507 | MAG_2410 |
| PG68 | 1 | Hypothetical protein MAG_1890 | *M. agalactiae* PG2 | 36683 | 8.78 | 118 | MALDI-TOF | 9 | 34% | gi|148377455 | MAG_1890 |
| PG69 | 1 | NI |  |  |  |  |  |  |  |  |  |
| PG70 | 1 | Glyceraldehyde-3-phosphate dehydrogenase A | *E. coli* | 35681 | 6.61 | 55 | ESI Q-TOF | 1 | 4% | G3P1_ECO57 |  |
| PG71 | 1 | NI |  |  |  |  |  |  |  |  |  |
| PG72 | 1 | Attachment protein | *M. genitalium* | 21972 | 5.35 | 30 | ESI Q-TOF | 1 | 5% | gi|82906922 |  |
| PG73 | 1 | NI |  |  |  |  |  |  |  |  |  |
| PG74 | 1 | Lipoprotein MAG_2430 | *M. agalactiae* PG2 | 33862 | 8.29 | 56 | MALDI-TOF | 5 | 24% | gi|148377509 | MAG_2430 |
| PG75 | 1 | ICEF-IIA ORF19 | *M. fermentans* | 81544 | 8.20 | 25 | ESI Q-TOF | 1 | 1% | gi|26984117 |  |
| PG76 | 1 | NI |  |  |  |  |  |  |  |  |  |
| PG77 | 1 | NI |  |  |  |  |  |  |  |  |  |
| PG78 | 1 | Hypothetical protein mhp361 | *M. hyopneumoniae* 232 | 38818 | 7.52 | 29 | ESI Q-TOF | 2 | 3% | gi|54020475 | mhp361 |
| PG79 | 1 | NI |  |  |  |  |  |  |  |  |  |
| PG80 | 1 | NI |  |  |  |  |  |  |  |  |  |
| PG81 | 1 | NI |  |  |  |  |  |  |  |  |  |
| PG82 | 1 | NI |  |  |  |  |  |  |  |  |  |
| PG83 | 1 | Lipoprotein MAG_1980 | *M. agalactiae* PG2 | 53788 | 8.98 | 58 | MALDI-TOF | 5 | 13% | gi|148377464 | MAG_1980 |
| PG84 | 1 | Variable surface lipoprotein V | *M. agalactiae* PG2 | 37362 | 9.25 | 48 | ESI Q-TOF | 1 | 2% | gi|23683074 | MAG_7050 |
| PG85 | 1 | Variable surface lipoprotein Y | *M. agalactiae* PG2 | 37528 | 8.81 | 63 | ESI Q-TOF | 1 | 3% | gi|148377970 | MAG_7080 |
| PG86 | 1 | Lipoprotein MAG_1050 | *M. agalactiae* PG2 | 37025 | 9.26 | 56 | MALDI-TOF | 5 | 23% | gi|148377373 | MAG_1050 |
| PG87 | 1 | Lipoprotein MAG_2430 | *M. agalactiae* PG2 | 33862 | 8.29 | 139 | MALDI-TOF | 9 | 36% | gi|148377509 | MAG_2430 |
| PG88 | 1 | Lipoprotein MAG_2430 | *M. agalactiae* PG2 | 33862 | 8.29 | 149 | MALDI-TOF | 9 | 32% | gi|148377509 | MAG_2430 |
| PG89 | 1 | NI |  |  |  |  |  |  |  |  |  |
| PG90 | 1 | Hypothetical protein MAG_1220 | *M. agalactiae* PG2 | 26491 | 8.69 | 47 | ESI Q-TOF | 2 | 8% | gi|148377389 | MAG_1220 |
| PG91 | 1 | Hypothetical protein MAG_1220 | *M. agalactiae* PG2 | 26491 | 8.69 | 97 | ESI Q-TOF | 5 | 15% | gi|148377389 | MAG_1220 |
| PG92 | 1 | Lipoprotein MAG_6200 | *M. agalactiae* PG2 | 26952 | 9.22 | 86 | ESI Q-TOF | 7 | 28% | gi|148377883 | MAG_6200 |
|  |  | Hypothetical protein MAG_1220 | *M. agalactiae* PG2 | 26491 | 8.69 | 54 | ESI Q-TOF | 3 | 12% | gi|148377389 | MAG_1220 |
|  |  | Transcription Elongation factor GreA | *M. capricolum subsp. capricolum* ATCC 27343 | 17483 | 5.84 | 33 | ESI Q-TOF | 1 | 5% | gi|83319925 |  |
|  |  | Lipoate-protein ligase A | *M. agalactiae* PG2 | 37286 | 6.42 | 29 | ESI Q-TOF | 2 | 2% | gi|148377329 | MAG_0600 |
| PG93 | 1 | Lipoprotein MAG_6200 | *M. agalactiae* PG2 | 26952 | 9.22 | 30 | ESI Q-TOF | 3 | 11% | gi|148377883 | MAG_6200 |
| PG94 | 1 | Lipoprotein MAG_6200 | *M. agalactiae* PG2 | 26952 | 9.22 | 29 | ESI Q-TOF | 1 | 3% | gi|148377883 | MAG_6200 |
| PG95 | 1 | Lipoprotein MAG_6200 | *M. agalactiae* PG2 | 26952 | 9.22 | 72 | ESI Q-TOF | 3 | 13% | gi|148377883 | MAG_6200 |
| PG96 | 1 | Variable surface lipoprotein D | *M. agalactiae* PG2 | 36608 | 9.53 | 129 | ESI Q-TOF | 8 | 9% | gi|148377972 | MAG_7100 |
| PG97 | 1 | Variable surface lipoprotein D | *M. agalactiae* PG2 | 36608 | 9.53 | 60 | MALDI-TOF | 7 | 18% | gi|148377972 | MAG_7100 |
| PG98 | 1 | Variable surface lipoprotein D | *M. agalactiae* PG2 | 36608 | 9.53 | 77 | MALDI-TOF | 8 | 21% | gi|148377972 | MAG_7100 |
| PG99 | 1 | Variable surface lipoprotein D | *M. agalactiae* PG2 | 36608 | 9.53 | 190 | ESI Q-TOF | 7 | 11% | gi|148377972 | MAG_7100 |
| PG100 | 1 | NI |  |  |  |  |  |  |  |  |  |
| PG101 | 1 | NI |  |  |  |  |  |  |  |  |  |
| PG102 | 1 | Lipoprotein MAG_2000 | *M. agalactiae* PG2 | 26602 | 8.39 | 69 | ESI Q-TOF | 1 | 6% | gi|148377466 | MAG_2000 |
| PG103 | 1 | Lipoprotein MAG_3600 | *M. agalactiae* PG2 | 21678 | 9.13 | 138 | ESI Q-TOF | 7 | 25% | gi|148377626 | MAG_3600 |
| PG104 | 1 | Lipoprotein MAG_3600 | *M. agalactiae* PG2 | 21678 | 9.13 | 533 | ESI Q-TOF | 22 | 58% | gi|148377626 | MAG_3600 |
|  |  | Endonuclease IV | *M. penetrans* HF-2 | 35632 | 6.22 | 33 | ESI Q-TOF | 1 | 4% | gi|26553573 |  |
| PG105 | 1 | Lipoprotein MAG_3600 | *M. agalactiae* PG2 | 21678 | 9.13 | 142 | ESI Q-TOF | 9 | 36% | gi|148377626 | MAG_3600 |
|  |  | endonuclease IV | *M. penetrans* HF-2 | 35632 | 6.22 | 26 | ESI Q-TOF | 1 | 4% | gi|26553573 |  |
| PG106 | 1 | Lipoprotein MAG_2400 | *M. agalactiae* PG2 | 38065 | 8.95 | 179 | ESI Q-TOF | 9 | 20% | gi|148377506 | MAG_2400 |
| PG107 | 1 | NI |  |  |  |  |  |  |  |  |  |
| PG108 | 1 | Variable surface lipoprotein Y | *M. agalactiae* PG2 | 37528 | 8.81 | 831 | ESI Q-TOF | 26 | 21% | gi|148377970 | MAG_7080 |
| PG109 | 1 | Variable surface lipoprotein Y | *M. agalactiae* PG2 | 37528 | 8.81 | 153 | ESI Q-TOF | 3 | 6% | gi|148377970 | MAG_7080 |
| PG110 | 1 | NI |  |  |  |  |  |  |  |  |  |
| PG111 | 1 | ATP synthase B chain | *M. agalactiae* PG2 | 21796 | 9.4 | 50 | ESI Q-TOF | 1 | 6% | gi|148377618 | MAG_3520 |
| PG112 | 1 | ATP synthase B chain | *M. agalactiae* PG2 | 21796 | 9.4 | 29 | ESI Q-TOF | 1 | 6% | gi|148377618 | MAG_3520 |
| PG113 | 1 | ATP synthase B chain | *M. agalactiae* PG2 | 21796 | 9.4 | 27 | ESI Q-TOF | 1 | 6% | gi|148377618 | MAG_3520 |
| PG114 | 1 | Variable surface lipoprotein A | *M. agalactiae* PG2 | 24769 | 8.33 | 85 | MALDI-TOF | 5 | 29% | gi|148377969 | MAG_7070 |
| PG115 | 1 | NI |  |  |  |  |  |  |  |  |  |
| PG116 | 1 | Variable surface lipoprotein A | *M. agalactiae* PG2 | 24769 | 8.33 | 60 | MALDI-TOF | 4 | 24% | gi|148377969 | MAG_7070 |
| PG117 | 1 | Variable surface lipoprotein A | *M. agalactiae* PG2 | 24769 | 8.33 | 62 | MALDI-TOF | 4 | 24% | gi|148377969 | MAG_7070 |
| P-1 | 2 | NI |  |  |  |  |  |  |  |  |  |
| P-2 | 2 | NI |  |  |  |  |  |  |  |  |  |
| P-3 | 2 | Hypotetical protein MAG_1000 | *M. agalactiae PG2* | 109727 | 8.66 | 155 | MALDI-TOF | 21 | 25% | gi|148377368 | MAG_1000 |
| P-4 | 2 | Hypotetical protein MAG_1000 | *M. agalactiae PG2* | 109727 | 8.66 | 71 | MALDI-TOF | 10 | 18% | gi|148377368 | MAG_1000 |
| P-5 | 2 | Hypotetical protein MAG_1000 | *M. agalactiae PG2* | 109727 | 8.66 | 175 | MALDI-TOF | 24 | 25% | gi|148377368 | MAG_1000 |
| P-6 | 2 | Hypotetical protein MAG_1000 | *M. agalactiae PG2* | 109727 | 8.66 | 199 | MALDI-TOF | 23 | 25% | gi|148377368 | MAG_1000 |
| P-7 | 2 | Hypotetical protein MAG_1000 | *M. agalactiae PG2* | 109727 | 8.66 | 91 | MALDI-TOF | 13 | 11% | gi|148377368 | MAG_1000 |
| P-8 | 2 | P80. lipoprotein | *M. agalactiae PG2* | 81102 | 9.08 | 219 | MALDI-TOF | 23 | 35% | gi|148377765 | MAG_5030 |
| P-9 | 2 | P80. lipoprotein | *M. agalactiae PG2* | 81102 | 9.08 | 181 | MALDI-TOF | 23 | 35% | gi|13992493 | MAG_5030 |
| P-10 | 2 | P80. lipoprotein | *M. agalactiae PG2* | 81102 | 9.08 | 229 | MALDI-TOF | 23 | 35% | gi|13992493 | MAG_5030 |
| P-11 | 2 | P80. lipoprotein | *M. agalactiae PG2* | 81102 | 9.08 | 245 | MALDI-TOF | 23 | 35% | gi|13992493 | MAG_5030 |
| P-12 | 2 | NI |  |  |  |  |  |  |  |  |  |
| P-13 | 2 | 5'nucleotidase | *M. agalactiae PG2* | 76195 | 8.26 | 83 | ESI Q-TOF | 10 | 14% | gi|148377854 | MAG_5910 |
| P-14 | 2 | Hypothetical protein MAG_6520 | *M. agalactiae PG2* | 70087 | 8.58 | 62 | ESI Q-TOF | 4 | 6% | gi|148377915 | MAG_6520 |
|  |  | Hypothetical protein MAG_2220 | *M. agalactiae PG2* | 69573 | 8.78 | 40 | ESI Q-TOF | 6 | 8% | gi|148377488 | MAG_2220 |
| P-15 | 2 | Hypothetical protein MAG_2220 | *M. agalactiae PG2* | 69573 | 8.78 | 76 | MALDI-TOF | 9 | 22% | gi|148377488 | MAG_2220 |
| P-16 | 2 | Hypothetical protein MAG_2220 | *M. agalactiae PG2* | 69573 | 8.78 | 59 | MALDI-TOF | 6 | 17% | gi|148377488 | MAG_2220 |
| P-17 | 2 | NI |  |  |  |  |  |  |  |  |  |
| P-18 | 2 | Hypothetical protein MAG_2220 | *M. agalactiae PG2* | 69573 | 8.78 | 96 | MALDI-TOF | 11 | 24% | gi|148377488 | MAG_2220 |
| P-19 | 2 | Hypothetical protein MAG_2220 | *M. agalactiae PG2* | 69573 | 8.78 | 55 | MALDI-TOF | 6 | 14% | gi|148377488 | MAG_2220 |
| P-20 | 2 | P48. lipoprotein | *M. agalactiae PG2* | 51232 | 8.39 | 56 | MALDI-TOF | 7 | 21% | gi|148377280 | MAG_0120 |
| P-21 | 2 | NI |  |  |  |  |  |  |  |  |  |
| P-22 | 2 | NI |  |  |  |  |  |  |  |  |  |
| P-23 | 2 | NI |  |  |  |  |  |  |  |  |  |
| P-24 | 2 | NI |  |  |  |  |  |  |  |  |  |
| P-25 | 2 | NI |  |  |  |  |  |  |  |  |  |
| P-26 | 2 | NI |  |  |  |  |  |  |  |  |  |
| P-27 | 2 | Lipoprotein MAG_1980 | *M. agalactiae PG2* | 53788 | 8.98 | 73 | MALDI-TOF | 9 | 23% | gi|148377464 | MAG_1980 |
| P-28 | 2 | Excinuclease ABC subunit B | *M. mycoides subsp. mycoides SC PG1* | 77534 | 8.03 | 68 | MALDI-TOF | 10 | 15% | gi|42561460 | MSC_0944 |
| P-29 | 2 | NI |  |  |  |  |  |  |  |  |  |
| P-30 | 2 | NI |  |  |  |  |  |  |  |  |  |
| P-31 | 2 | NI |  |  |  |  |  |  |  |  |  |
| P-32 | 2 | NI |  |  |  |  |  |  |  |  |  |
| P-33 | 2 | NI |  |  |  |  |  |  |  |  |  |
| P-34 | 2 | NI |  |  |  |  |  |  |  |  |  |
| P-35 | 2 | NI |  |  |  |  |  |  |  |  |  |
| P-36 | 2 | NI |  |  |  |  |  |  |  |  |  |
| P-37 | 2 | NI |  |  |  |  |  |  |  |  |  |
| P-38 | 2 | NI |  |  |  |  |  |  |  |  |  |
| P-39 | 2 | NI |  |  |  |  |  |  |  |  |  |
| P-40 | 2 | NI |  |  |  |  |  |  |  |  |  |
| P-41 | 2 | Lipoprotein MAG_2350 | *M. agalactiae PG2* | 40383 | 8.61 |  | ESI Q-TOF | 13 | 35% | gi|148291547 | MAG_2350 |
|  |  | Variable surface lipoprotein Y | *M. agalactiae PG2* | 37528 | 8.81 | 91 | MALDI-TOF | 10 | 27% | gi|148377970 | MAG_7080 |
| P-43 | 2 | Variable surface lipoprotein Y | *M. agalactiae PG2* | 37528 | 8.81 | 142 | MALDI-TOF | 13 | 38% | gi|148377970 | MAG_7080 |
| P-44 | 2 | Variable surface lipoprotein Y | *M. agalactiae PG2* | 37528 | 8.81 | 121 | MALDI-TOF | 10 | 35% | gi|148377970 | MAG_7080 |
| P-45 | 2 | NI |  |  |  |  |  |  |  |  |  |
| P-46 | 2 | Variable surface lipoprotein W | *M. agalactiae PG2* | 35472 | 9.54 | 82 | MALDI-TOF | 7 | 23% | gi|148377968 | MAG_7060 |
| P-47 | 2 | Variable surface lipoprotein W | *M. agalactiae PG2* | 35472 | 9.54 | 82 | MALDI-TOF | 7 | 21% | gi|148377968 | MAG_7060 |
| P-48 | 2 | Variable surface lipoprotein W | *M. agalactiae PG2* | 35472 | 9.54 | 82 | MALDI-TOF | 7 | 21% | gi|148377968 | MAG_7060 |
| P-49 | 2 | Variable surface lipoprotein W | *M. agalactiae PG2* | 35472 | 9.54 | 94 | MALDI-TOF | 8 | 23% | gi|148377968 | MAG_7060 |
| P-50 | 2 | Hypothetical protein MAG_1450 | *M. agalactiae PG2* | 35463 | 9.15 | 86 | ESI Q-TOF | 3 | 8% | gi|148377412 | MAG_1450 |
|  |  | Lipoprotein MAG_1050 | *M. agalactiae PG2* | 37025 | 9.26 | 49 | ESI Q-TOF | 1 | 3% | gi|148377373 | MAG_1050 |
| P-51 | 2 | 5'nucleotidase | *M. agalactiae PG2* | 76195 | 8.26 | 52 | ESI Q-TOF | 3 | 3% | gi|148377854 | MAG_5910 |
| P-52 | 2 | 5'nucleotidase | *M. agalactiae PG2* | 76195 | 8.26 | 65 | ESI Q-TOF | 4 | 4% | gi|148377854 | MAG_5910 |
| P-53 | 2 | Lipoprotein MAG_1050 | *M. agalactiae PG2* | 37025 | 9.26 | 76 | MALDI-TOF | 8 | 26% | gi|148377373 | MAG_1050 |
| P-54 | 2 | Lipoprotein MAG_1050 | *M. agalactiae PG2* | 37025 | 9.26 | 233 | MALDI-TOF | 20 | 53% | gi|148377373 | MAG_1050 |
| P-55 | 2 | Lipoprotein MAG_1050 | *M. agalactiae PG2* | 37025 | 9.26 | 203 | MALDI-TOF | 15 | 45% | gi|148377373 | MAG_1050 |
| P-56 | 2 | Lipoprotein MAG_1050 | *M. agalactiae PG2* | 37025 | 9.26 | 57 | MALDI-TOF | 6 | 21% | gi|148377373 | MAG_1050 |
| P-57 | 2 | Putative nicotinate-nucleotide adenylyltransferase | *M. pneumoniae M129* | 40168 | 9.79 | 55 | MALDI-TOF | 6 | 24% | gi|161723288 |  |
| P-58 | 2 | Lipoprotein | *M. agalactiae PG2* | 37025 | 9.26 | 83 | ESI Q-TOF | 5 | 12% | gi|148377373 | MAG_1050 |
| P-59 | 2 | Lipoprotein | *M. agalactiae PG2* | 37025 | 9.26 | 72 | MALDI-TOF | 6 | 26% | gi|148377373 | MAG_1050 |
| P-60 | 2 | NI |  |  |  |  |  |  |  |  |  |
| P-61 | 2 | NI |  |  |  |  |  |  |  |  |  |
| P-62 | 2 | Variable surface lipoprotein W | *M. agalactiae PG2* | 35472 | 9.54 | 28 | ESI Q-TOF | 1 | 3% | gi|148377968 | MAG_7060 |
| P-63 | 2 | 50S ribosomal protein L3 | *M. agalactiae PG2* | 28793 | 9.75 | 50 | ESI Q-TOF | 2 | 4% | gi|148377809 | MAG_5460 |
| P-64 | 2 | NI |  |  |  |  |  |  |  |  |  |
| P-65 | 2 | NI |  |  |  |  |  |  |  |  |  |
| P-66 | 2 | NI |  |  |  |  |  |  |  |  |  |
| P-67 | 2 | NI |  |  |  |  |  |  |  |  |  |
| P-68 | 2 | NI |  |  |  |  |  |  |  |  |  |
| P-69 | 2 | NI |  |  |  |  |  |  |  |  |  |
| P-70 | 2 | NI |  |  |  |  |  |  |  |  |  |
| P-71 | 2 | Lipoprotein MAG_6200 | *M. agalactiae PG2* | 26952 | 9.22 | 37 | ESI Q-TOF | 1 | 3% | gi|148377883 | MAG_6200 |
|  |  | Hypothetical protein MAG_1220 | *M. agalactiae PG2* | 26491 | 8.69 | 30 | ESI Q-TOF | 1 | 3% | gi|148377389 | MAG_1220 |
| P-72 | 2 | Lipoprotein MAG_6200 | *M. agalactiae PG2* | 26952 | 9.22 | 35 | ESI Q-TOF | 1 | 3% | gi|148377883 | MAG_6200 |
|  | 2 | Hypothetical protein MAG_1220 | *M. agalactiae PG2* | 26491 | 8.69 |  | ESI Q-TOF | 1 | 3% | gi|148377389 | MAG_1220 |
| P-73 | 2 | Hypothetical protein MAG_1220 | *M. agalactiae PG2* | 26491 | 8.69 | 35 | ESI Q-TOF | 1 | 3% | gi|148377389 | MAG_1220 |
| P-74 | 2 | Lipoprotein MAG_6200 | *M. agalactiae PG2* | 26952 | 9.22 | 81 | ESI Q-TOF | 3 | 11% | gi|148377883 | MAG_6200 |
| P-75 | 2 | NI |  |  |  |  |  |  |  |  |  |
| P-76 | 2 | Lipoprotein MAG_6200 | *M. agalactiae PG2* | 26952 | 9.22 | 88 | ESI Q-TOF | 3 | 11% | gi|148377883 | MAG_6200 |
| P-77 | 2 | NI |  |  |  |  |  |  |  |  |  |
| P-78 | 2 | NI |  |  |  |  |  |  |  |  |  |
| P-79 | 2 | NI |  |  |  |  |  |  |  |  |  |
| P-80 | 2 | NI |  |  |  |  |  |  |  |  |  |
| P-81 | 2 | NI |  |  |  |  |  |  |  |  |  |
| P-82 | 2 | NI |  |  |  |  |  |  |  |  |  |
| P-83 | 2 | 50S ribosomal protein L1 | *M. agalactiae PG2* | 24955 | 9.68 | 62 | ESI Q-TOF | 1 | 4% | gi|148377349 | MAG_0810 |
| P-84 | 2 | Hypothetical lipoprotein | *M. arthritidis 158L3-1* | 77114 | 8.42 | 28 | ESI Q-TOF | 1 | 1% | gi|193217020 |  |
| P-85 | 2 | NI |  |  |  |  |  |  |  |  |  |
| P-86 | 2 | NI |  |  |  |  |  |  |  |  |  |
| P-87 | 2 | Lipoprotein. MAG_4740 | *M. agalactiae PG2* | 25452 | 9.47 | 71 | MALDI-TOF | 8 | 37% | gi|148377737 | MAG_4740 |
| P-88 | 2 | Lipoprotein MAG_4740 | *M. agalactiae PG2* | 25452 | 9.47 | 68 | MALDI-TOF | 6 | 27% | gi|148377737 | MAG_4740 |
| P-89 | 2 | 30S ribosomal protein S8 | *M. agalactiae PG2* | 24090 | 9.91 | 151 | ESI Q-TOF | 8 | 22% | gi|148377803 | MAG_5400 |
| P-90 | 2 | Lipoprotein MAG_3600 | *M. agalactiae PG2* | 21678 | 9.13 | 70 | ESI Q-TOF | 2 | 9% | gi|148377626 | MAG_3600 |
| P-91 | 2 | Lipoprotein MAG_3600 | *M. agalactiae PG2* | 21678 | 9.13 | 53 | ESI Q-TOF | 3 | 9% | gi|148377626 | MAG_3600 |
| P-92 | 2 | Lipoprotein MAG_3600 | *M. agalactiae PG2* | 21678 | 9.13 | 150 | ESI Q-TOF | 5 | 15% | gi|148377626 | MAG_3600 |
| P-93 | 2 | Lipoprotein MAG_3600 | *M. agalactiae PG2* | 21678 | 9.13 | 55 | MALDI-TOF | 5 | 35% | gi|148377626 | MAG_3600 |
| P-94 | 2 | Lipoprotein MAG_3600 | *M. agalactiae PG2* | 21678 | 9.13 | 412 | ESI Q-TOF | 12 | 52% | gi|148377626 | MAG_3600 |
| P-95 | 2 | Lipoprotein MAG_3600 | *M. agalactiae PG2* | 21678 | 9.13 | 64 | MALDI-TOF | 6 | 35% | gi|148377626 | MAG_3600 |
| P-96 | 2 | Lipoprotein MAG_3600 | *M. agalactiae PG2* | 21678 | 9.13 | 57 | MALDI-TOF | 6 | 38% | gi|148377626 | MAG_3600 |
| P-97 | 2 | NI |  |  |  |  |  |  |  |  |  |
| P-98 | 2 | NI |  |  |  |  |  |  |  |  |  |
| P-99 | 2 | NI |  |  |  |  |  |  |  |  |  |
| P-100 | 2 | Lipoprotein MAG_2400 | *M. agalactiae PG2* | 38065 | 8.95 | 37 | ESI Q-TOF | 4 | 11% | gi|148377506 | MAG_2400 |
| P-101 | 2 | Lipoprotein MAG_2400 | *M. agalactiae PG2* | 38065 | 8.95 | 111 | ESI Q-TOF | 11 | 15% | gi|148377506 | MAG_2400 |
| P-102 | 2 | NI |  |  |  |  |  |  |  |  |  |
| P-103 | 2 | NI |  |  |  |  |  |  |  |  |  |
| P-104 | 2 | ATP synthase B chain | *M. agalactiae PG2* | 21796 | 9.4 | 33 | ESI Q-TOF | 1 | 6% | gi|148377618 | MAG_3520 |
| P-105 | 2 | NI |  |  |  |  |  |  |  |  |  |
| P-106 | 2 | NI |  |  |  |  |  |  |  |  |  |
| P-107 | 2 | NI |  |  |  |  |  |  |  |  |  |
| P-108 | 2 | NI |  |  |  |  |  |  |  |  |  |
| P-109 | 2 | NI |  |  |  |  |  |  |  |  |  |
| P-110 | 2 | NI |  |  |  |  |  |  |  |  |  |
| P-111 | 2 | NI |  |  |  |  |  |  |  |  |  |
| P-112 | 2 | 5'nucleotidase | *M. agalactiae PG2* | 76195 | 8.26 | 116 | ESI Q-TOF | 6 | 8% | gi|148377854 | MAG_5910 |
| P-113 | 3 | Hypotetical protein MAG_1000 | *M. agalactiae PG2* | 109727 | 8.66 | 168 | MALDI-TOF | 20 | 23% | gi|148377368 | MAG_1000 |
| P-114 | 3 | P80. lipoprotein | *M. agalactiae PG2* | 81102 | 9.08 | 200 | MALDI-TOF | 24 | 37% | gi|148377765 | MAG_5030 |
| P-115 | 3 | 5'nucleotidase | *M. agalactiae PG2* | 76195 | 8.36 | 91 | MALDI-TOF | 11 | 21% | gi|148377854 | MAG_5910 |
| P-116 | 3 | Hypothetical protein MAG_2220 | *M. agalactiae PG2* | 69573 | 8.78 | 85 | MALDI-TOF | 10 | 20% | gi|148377488 | MAG_2220 |
| P-117 | 3 | P80. lipoprotein | *M. agalactiae PG2* | 81102 | 9.08 | 74 | ESI Q-TOF | 5 | 6% | gi|148377765 | MAG_5030 |
| P-118 | 3 | P80. lipoprotein | *M. agalactiae PG2* | 81102 | 9.08 | 136 | ESI Q-TOF | 10 | 11% | gi|148377765 | MAG_5030 |
| P-119 | 3 | Lipoprotein | *M. agalactiae PG2* | 59415 | 6.77 | 58 | ESI Q-TOF | 7 | 10% | gi|148377770 | MAG_5080 |
| P-120 | 3 | NI |  |  |  |  |  |  |  |  |  |
| P-121 | 3 | Lipoprotein MAG_5080 | *M. agalactiae PG2* | 59415 | 6.77 | 395 | ESI Q-TOF | 17 | 30% | gi|148377770 | MAG_5080 |
| P-122 | 3 | Lipoprotein MAG_5080 | *M. agalactiae PG2* | 59415 | 6.77 | 63 | MALDI-TOF | 6 | 17% | gi|148377770 | MAG_5080 |
| P-123 | 3 | Lipoprotein MAG_5080 | *M. agalactiae PG2* | 59415 | 6.77 | 132 | ESI Q-TOF | 6 | 8% | gi|148377770 | MAG_5080 |
| P-124 | 3 | NI |  |  |  |  |  |  |  |  |  |
| P-125 | 3 | NI |  |  |  |  |  |  |  |  |  |
| P-126 | 3 | NADH oxidase (NOXASE) | *M. agalactiae PG2* | 49980 | 6.09 | 39 | ESI Q-TOF | 2 | 3% | gi|148377529 | MAG_2630 |
| P-127 | 3 | NADH oxidase (NOXASE) | *M. agalactiae PG2* | 49980 | 6.09 | 33 | ESI Q-TOF | 2 | 3% | gi|148377529 | MAG_2630 |
| P-128 | 3 | P48. lipoprotein | *M. agalactiae PG2* | 51232 | 8.39 | 46 | ESI Q-TOF | 2 | 4% | gi|148377280 | MAG_0120 |
| P-129 | 3 | NI |  |  |  |  |  |  |  |  |  |
| P-130 | 3 | P48. lipoprotein | *M. agalactiae PG2* | 51232 | 8.39 | 149 | ESI Q-TOF | 5 | 10% | gi|148377280 | MAG_0120 |
| P-131 | 3 | NI |  |  |  |  |  |  |  |  |  |
| P-132 | 3 | P48. lipoprotein | *M. agalactiae PG2* | 51232 | 8.39 | 107 | MALDI-TOF | 11 | 26% | gi|148377280 | MAG_0120 |
| P-133 | 3 | NI |  |  |  |  |  |  |  |  |  |
| P-134 | 3 | NI |  |  |  |  |  |  |  |  |  |
| P-135 | 3 | NI |  |  |  |  |  |  |  |  |  |
| P-136 | 3 | NI |  |  |  |  |  |  |  |  |  |
| P-137 | 3 | NI |  |  |  |  |  |  |  |  |  |
| P-138 | 3 | NI |  |  |  |  |  |  |  |  |  |
| P-139 | 3 | P48. lipoprotein | *M. agalactiae PG2* | 51232 | 7.19 | 149 | ESI Q-TOF | 5 | 10% | gi|148377280 | MAG_0120 |
| P-140 | 3 | NI |  |  |  |  |  |  |  |  |  |
| P-141 | 3 | Elongation factor Tu | *M. agalactiae PG2* | 43638 | 5.89 | 195 | MALDI-TOF | 14 | 41% | gi|148377586 | MAG_3200 |
| P-142 | 3 | Hypothetical protein MAG_5040 | *M. agalactiae PG2* | 44828 | 8.46 | 61 | MALDI-TOF | 9 | 21% | gi|148377766 | MAG_5040 |
| P-143 | 3 | P40, lipoprotein | *M. agalactiae PG2* | 39951 | 8.2 | 113 | MALDI-TOF | 11 | 33% | gi|148377507 | MAG_2410 |
| P-144 | 3 | P40, lipoprotein | *M. agalactiae PG2* | 39951 | 8.19 | 126 | MALDI-TOF | 11 | 33% | gi|148377507 | MAG_2410 |
| P-145 | 3 | P40, lipoprotein | *M. agalactiae PG2* | 39951 | 8.19 | 118 | MALDI-TOF | 10 | 34% | gi|148377507 | MAG_2410 |
| P-146 | 3 | P40, lipoprotein | *M. agalactiae PG2* | 39951 | 8.19 | 141 | MALDI-TOF | 11 | 37% | gi|148377507 | MAG_2410 |
| P-147 | 3 | NI |  |  |  |  |  |  |  |  |  |
| P-148 | 3 | P40, lipoprotein | *M. agalactiae PG2* | 39951 | 8.19 | 105 | MALDI-TOF | 9 | 30% | gi|148377507 | MAG_2410 |
| P-149 | 3 | Variable surface lipoprotein Y | *M. agalactiae PG2* | 37528 | 8.81 | 34 | ESI Q-TOF | 3 | 10% | gi|148377970 | MAG_7080 |
| P-150 | 3 | NI |  |  |  |  |  |  |  |  |  |
| P-151 | 3 | P40, lipoprotein | *M. agalactiae PG2* | 39951 | 8.19 | 136 | ESI Q-TOF | 6 | 16% | gi|148377507 | MAG_2410 |
|  |  | Lipoate-protein ligase A | *M. agalactiae PG2* | 37286 | 6.42 | 25 | ESI Q-TOF | 1 | 2% | gi|148377329 | MAG_0600 |
| P-152 | 3 | Pyruvate dehydrogenase E1 component. alphasubunit | *M. agalactiae PG2* | 41321 | 6.13 | 58 | ESI Q-TOF | 2 | 5% | gi|148377361 | MAG_0930 |
| P-153 | 3 | NI |  |  |  |  |  |  |  |  |  |
| P-154 | 3 | Pyruvate dehydrogenase E1 component. betasubunit | *M. agalactiae PG2* | 36129 | 5.44 | 57 | ESI Q-TOF | 2 | 3% | gi|148377362 | MAG_0940 |
| P-155 | 3 | Pyruvate dehydrogenase E1 component. betasubunit | *M. agalactiae PG2* | 36129 | 5.44 | 97 | MALDI-TOF | 10 | 41% | gi|148377362 | MAG_0940 |
| P-156 | 3 | Pyruvate dehydrogenase E1 component. betasubunit | *M. agalactiae PG2* | 36129 | 5.44 | 120 | MALDI-TOF | 13 | 46% | gi|148377362 | MAG_0940 |
| P-157 | 3 | Acyl carrier protein phosphodiesterase | *M. hyopneumoniae 232* | 25819 | 9.52 | 28 | ESI Q-TOF | 1 | 4% | gi|54020205 |  |
| P-158 | 3 | NI |  |  |  |  |  |  |  |  |  |
| P-159 | 3 | Triosephosphate isomerase | *M. hyopneumoniae 232* | 26804 | 8.33 | 29 | ESI Q-TOF | 1 | 3% | gi|54020428 |  |
| P-160 | 3 | D-lactate dehydrogenase | *M. agalactiae PG2* | 37021 | 6.11 | 34 | ESI Q-TOF | 1 | 2% | gi|148377416 | MAG_1490 |
| P-162 | 3 | Lipoprotein MAG_2430 | *M. agalactiae PG2* | 33862 | 8.29 | 36 | ESI Q-TOF | 2 | 6% | gi|148377509 | MAG_2430 |
|  |  | ICEF-IIA ORF19 | *M. fermentans* | 81544 | 8.2 | 25 | ESI Q-TOF | 1 | 1% | gi|26984117 |  |
| P-163 | 3 | Lipoprotein MAG_2430 | *M. agalactiae PG2* | 33862 | 8.29 | 73 | MALDI-TOF | 7 | 27% | gi|148377509 | MAG_2430 |
| P-164 | 3 | Lipoprotein MAG_2430 | *M. agalactiae PG2* | 33862 | 8.29 | 120 | MALDI-TOF | 10 | 36% | gi|148377509 | MAG_2430 |
| P-165 | 3 | Lipoprotein MAG_2430 | *M. agalactiae PG2* | 33862 | 8.29 | 117 | MALDI-TOF | 8 | 34% | gi|148377509 | MAG_2430 |
| P-166 | 3 | Lipoprotein MAG_2430 | *M. agalactiae PG2* | 33862 | 8.29 | 72 | MALDI-TOF | 6 | 27% | gi|148377509 | MAG_2430 |
| P-167 | 3 | Variable surface lipoprotein D | *M. agalactiae PG2* | 36608 | 9.53 | 30 | ESI Q-TOF | 1 | 2% | gi|148377972 | MAG_7100 |
| P-168 | 3 | Variable surface lipoprotein D | *M. agalactiae PG2* | 36608 | 9.53 | 129 | ESI Q-TOF | 8 | 9% | gi|148377972 | MAG_7100 |
| P-169 | 3 | Variable surface lipoprotein D | *M. agalactiae PG2* | 36608 | 9.53 | 60 | MALDI-TOF | 7 | 18% | gi|148377972 | MAG_7100 |
| P-170 | 3 | Variable surface lipoprotein D | *M. agalactiae PG2* | 36608 | 9.53 | 77 | MALDI-TOF | 8 | 21% | gi|148377972 | MAG_7100 |
| P-171 | 3 | Variable surface lipoprotein D | *M. agalactiae PG2* | 36608 | 9.53 | 190 | ESI Q-TOF | 7 | 11% | gi|148377972 | MAG_7100 |
| P-172 | 3 | NI |  |  |  |  |  |  |  |  |  |
| P-173 | 3 | NI |  |  |  |  |  |  |  |  |  |
| P-174 | 3 | Variable surface lipoprotein A | *M. agalactiae PG2* | 24770 | 8.33 | 64 | MALDI-TOF | 5 | 31% | gi|148377969 | MAG_7070 |
| P-175 | 3 | Variable surface lipoprotein A | *M. agalactiae PG2* | 24769 | 8.33 | 307 | ESI Q-TOF | 10 | 22% | gi|148377969 | MAG_7070 |
