# Supplementary material for: The liposoluble proteome of Mycoplasma agalactiae: an insight into the minimal protein complement of a bacterial membrane
Source: BMC Microbiol. 2010 Aug 25;10:225. doi: 10.1186/1471-2180-10-225 (PMC2941501; doi:10.1186/1471-2180-10-225)

**Additional file 5: Protein profile of liposolubile proteins before and after precipitation. Right: approach used for GeLC-MS/MS characterization.** The bars indicate the regions cut from the PAGE gel and subjected to mass spectrometry characterization. Protein identifications are reported in Additional file 6, from top to bottom.


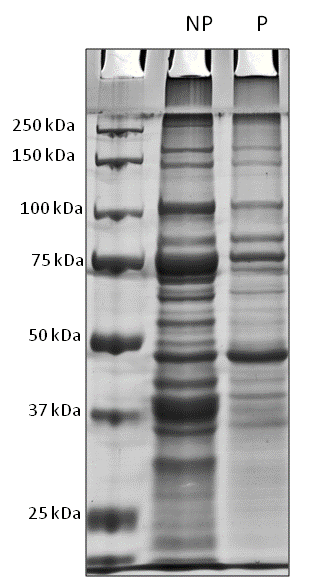

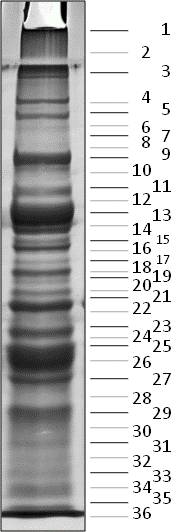

Supplement: Additional file 5 — Protein profile of liposoluble proteins before and after precipitation. Right: approach used for GeLC-MS/MS characterization. The bars indicate the regions cut from the PAGE gel and subjected to mass spectrometry characterization. Protein identifications are reported in additional file 6, from top to bottom. [file 1471-2180-10-225-S5.DOC]
