# Supplementary material for: The liposoluble proteome of Mycoplasma agalactiae: an insight into the minimal protein complement of a bacterial membrane
Source: BMC Microbiol. 2010 Aug 25;10:225. doi: 10.1186/1471-2180-10-225 (PMC2941501; doi:10.1186/1471-2180-10-225)
Supplement: Additional file 6 — Table listing all protein identifications obtained by GeLC-MS/MS of the M. agalactiae PG2T Triton X-114 liposoluble fraction. The protein profile used and the number of slices are reported in Additional file 5. [file 1471-2180-10-225-S6.DOC]

### Additional file 6: Table listing all protein identifications obtained by GeLC-MS/MS of the *M. agalactiae* PG2^T^ Triton X-114 liposoluble fraction. The protein profile used and the number of slices are represented in Additional file 5.

| **Band** | **Protein** | **Organism** | **Mw (Da)** | **pI** | **Score** | **Queries** | **Coverage** | **Acc. No**. | **Locus** |
| --- | --- | --- | --- | --- | --- | --- | --- | --- | --- |
| 1 | Malate permease | *M. agalactiae* PG2 | 45146 | 9.75 | 274 | 5 | 9% | gi|148377751 | MAG_4890 |
|  | Elongation factor Tu | *M. agalactiae* PG2 | 43638 | 5.89 | 261 | 7 | 17% | gi|148377586 | MAG_3200 |
|  | L-lactate dehydrogenase (L-LDH) | *M. agalactiae* PG2 | 34965 | 6.13 | 189 | 6 | 18% | gi|148377752 | MAG_4900 |
|  | Sugar ABC transporter permease | *M. agalactiae* PG2 | 74657 | 9.45 | 179 | 9 | 11% | gi|148377283 | MAG_0150 |
|  | ABC transporter ATP-binding protein | *M. agalactiae* PG2 | 80105 | 9.01 | 176 | 3 | 5% | gi|148377767 | MAG_5050 |
|  | Dihydrolipoamide dehydrogenase (E3 component of pyruvate complex) | *M. agalactiae* PG2 | 58917 | 6.36 | 173 | 4 | 6% | gi|148377364 | MAG_0960 |
|  | NADH oxidase (NOXASE) | *M. agalactiae* PG2 | 49980 | 6.09 | 161 | 6 | 15% | gi|148377529 | MAG_2630 |
|  | Putative inner membrane protein translocase component YidC | *M. agalactiae* PG2 | 83003 | 9.48 | 111 | 6 | 7% | gi|148377328 | MAG_0590 |
|  | Hypothetical protein MAG_0280 | *M. agalactiae* PG2 | 75681 | 9.03 | 104 | 2 | 2% | gi|148377296 | MAG_0280 |
|  | Preprotein translocase subunit SecY | *M. agalactiae* PG2 | 54823 | 9.7 | 102 | 2 | 3% | gi|148377788 | MAG_5260 |
|  | Cation-transporting P-type ATPase | *M. agalactiae* PG2 | 100888 | 6.32 | 94 | 3 | 3% | gi|148377722 | MAG_4590 |
|  | ABC transporter. ATP-binding protein | *M. agalactiae* PG2 | 68597 | 9.25 | 94 | 1 | 2% | gi|148377862 | MAG_5990 |
|  | Protein-export membrane protein | *M. agalactiae* PG2 | 89106 | 9.52 | 82 | 5 | 5% | gi|148377491 | MAG_2250 |
|  | ABC transporter permease protein | *M. agalactiae* PG2 | 39653 | 9.72 | 74 | 1 | 3% | gi|148377768 | MAG_5060 |
|  | ABC transporter. ATP-binding protein | *M. agalactiae* PG2 | 67154 | 8.52 | 69 | 1 | 1% | gi|148377863 | MAG_6000 |
|  | ABC transporter. ATP-binding protein. P59 | *M. agalactiae* PG2 | 58997 | 6.43 | 67 | 3 | 6% | gi|148377282 | MAG_0140 |
|  | Hypothetical protein MAG_2680 | *M. agalactiae* PG2 | 55645 | 4.72 | 65 | 1 | 2% | gi|148377534 | MAG_2680 |
|  | Oligopeptide ABC transporter system. permeaseprotein (OppC) | *M. agalactiae* PG2 | 47514 | 8.85 | 55 | 4 | 8% | gi|148377370 | MAG_1020 |
|  | DNA gyrase subunit A | *M. agalactiae* PG2 | 103247 | 5.33 | 54 | 2 | 2% | gi|148377826 | MAG_5630 |
|  | Pyruvate dehydrogenase E1 component. betasubunit | *M. agalactiae* PG2 | 36129 | 5.44 | 52 | 1 | 3% | gi|148377362 | MAG_0940 |
|  | Pyruvate dehydrogenase E1 component. alphasubunit | *M. agalactiae* PG2 | 41321 | 6.13 | 51 | 2 | 5% | gi|148377361 | MAG_0930 |
|  | ABC transporter permease protein | *M. agalactiae* PG2 | 37630 | 9.89 | 47 | 1 | 2% | gi|148377769 | MAG_5070 |
|  | 50S ribosomal protein L1 | *M. agalactiae* PG2 | 24955 | 9.68 | 44 | 2 | 7% | gi|148377349 | MAG_0810 |
|  | Glycerol ABC transporter. ATP-binding protein | *M. capricolum* subsp. *capricolum* ATCC 27343 | 47229 | 9.59 | 43 | 2 | 6% | gi|83319663 | MCAP_0454 |
|  | Ascorbate-specific PTS system enzyme IIC | *M. agalactiae* PG2 | 64257 | 9.33 | 42 | 1 | 3% | gi|148377901 | MAG_6380 |
|  | 30S ribosomal protein S8 | *M. agalactiae* PG2 | 24090 | 9.91 | 38 | 1 | 3% | gi|148377803 | MAG_5400 |
|  | 50S ribosomal protein L2 | *M. agalactiae* PG2 | 30773 | 10.63 | 38 | 2 | 4% | gi|148377806 | MAG_5430 |
|  | Cation-transporting P-ATPase | *M. agalactiae* PG2 | 100451 | 8.6 | 31 | 1 | 1% | gi|148378004 | MAG_7420 |
|  | Hexosephosphate transport protein | *M. agalactiae* PG2 | 54592 | 9.81 | 30 | 1 | 1% | gi|148377759 | MAG_4970 |
|  | Hypothetical protein MAG_4530 | *M. agalactiae* PG2 | 52907 | 9.51 | 29 | 1 | 2% | gi|148377716 | MAG_4530 |
|  | ABC transporter. permease protein | *M. agalactiae* PG2 | 71089 | 8.87 | 27 | 1 | 1% | gi|148377723 | MAG_4600 |
|  | Lipoate-protein ligase A | *M. agalactiae* PG2 | 37286 | 6.42 | 26 | 2 | 2% | gi|148377329 | MAG_0600 |
|  |  |  |  |  |  |  |  |  |  |
| 2 | L-lactate dehydrogenase (L-LDH) | *M. agalactiae* PG2 | 34965 | 6.13 | 121 | 5 | 14% | gi|148377752 | MAG_4900 |
|  | Hypothetical protein MAG_0280 | *M. agalactiae* PG2 | 75681 | 9.03 | 116 | 2 | 2% | gi|148377296 | MAG_0280 |
|  | Malate permease | *M. agalactiae* PG2 | 45146 | 9.75 | 111 | 2 | 6% | gi|148377751 | MAG_4890 |
|  | ABC transporter. ATP-binding protein | *M. agalactiae* PG2 | 68597 | 9.25 | 106 | 1 | 2% | gi|148377862 | MAG_5990 |
|  | ABC transporter permease protein | *M. agalactiae* PG2 | 39653 | 9.72 | 94 | 2 | 6% | gi|148377768 | MAG_5060 |
|  | Cation-transporting P-type ATPase | *M. agalactiae* PG2 | 100888 | 6.32 | 88 | 4 | 4% | gi|148377722 | MAG_4590 |
|  | Sugar ABC transporter permease | *M. agalactiae* PG2 | 74657 | 9.45 | 85 | 3 | 4% | gi|148377283 | MAG_0150 |
|  | ABC transporter ATP-binding protein | *M. agalactiae* PG2 | 80105 | 9.01 | 83 | 1 | 1% | gi|148377767 | MAG_5050 |
|  | Hypothetical protein MAG_2680 | *M. agalactiae* PG2 | 55645 | 4.72 | 79 | 1 | 2% | gi|148377534 | MAG_2680 |
|  | XAA-Pro aminopeptidase | *M. agalactiae* PG2 | 40001 | 5.88 | 78 | 2 | 6% | gi|148377385 | MAG_1180 |
|  | ABC transporter. permease protein | *M. agalactiae* PG2 | 71089 | 8.87 | 66 | 1 | 1% | gi|148377723 | MAG_4600 |
|  | Preprotein translocase subunit SecY | *M. agalactiae* PG2 | 54823 | 9.7 | 62 | 1 | 2% | gi|148377788 | MAG_5260 |
|  | Putative inner membrane protein translocase component YidC | *M. agalactiae* PG2 | 83003 | 9.48 | 51 | 3 | 3% | gi|148377328 | MAG_0590 |
|  | Pyruvate dehydrogenase E1 component. alphasubunit | *M. agalactiae* PG2 | 41321 | 6.13 | 50 | 1 | 3% | gi|148377361 | MAG_0930 |
|  | Dihydrolipoamide dehydrogenase (E3 component of pyruvate complex) | *M. agalactiae* PG2 | 58917 | 6.36 | 45 | 2 | 3% | gi|148377364 | MAG_0960 |
|  | ABC transporter permease protein | *M. agalactiae* PG2 | 37630 | 9.89 | 39 | 1 | 2% | gi|148377769 | MAG_5070 |
|  | Elongation factor Tu | *M. agalactiae* PG2 | 43638 | 5.89 | 31 | 1 | 2% | gi|148377586 | MAG_3200 |
|  | Hypothetical protein MYPU_3820 | *M. pulmonis* UAB CTIP | 140043 | 8.49 | 30 | 1 | 0% | gi|15828853 | MAG_3820 |
|  | Protein-export membrane protein | *M. agalactiae* PG2 | 89106 | 9.52 | 30 | 2 | 2% | gi|148377491 | MAG_2250 |
|  | ABC transporter. ATP-binding protein | *M. agalactiae* PG2 | 67154 | 8.52 | 29 | 1 | 1% | gi|148377863 | MAG_6000 |
|  |  |  |  |  |  |  |  |  |  |
| 3 | Elongation factor Tu | *M. agalactiae* PG2 | 43638 | 5.89 | 299 | 6 | 17% | gi|148377586 | MAG_3200 |
|  | Sugar ABC transporter permease | *M. agalactiae* PG2 | 74657 | 9.45 | 187 | 10 | 10% | gi|148377283 | MAG_0150 |
|  | ABC transporter permease protein | *M. agalactiae* PG2 | 308764 | 8.86 | 178 | 5 | 1% | gi|148378006 | MAG_7440 |
|  | Putative inner membrane protein translocase component YidC | *M. agalactiae* PG2 | 83003 | 9.48 | 158 | 6 | 6% | gi|148377328 | MAG_0590 |
|  | Hypothetical protein MAG_0280 | *M. agalactiae* PG2 | 75681 | 9.03 | 155 | 4 | 2% | gi|148377296 | MAG_0280 |
|  | Cation-transporting P-type ATPase | *M. agalactiae* PG2 | 100888 | 6.32 | 154 | 4 | 4% | gi|148377722 | MAG_4590 |
|  | ABC transporter permease protein | *M. agalactiae* PG2 | 39653 | 9.72 | 152 | 2 | 3% | gi|148377768 | MAG_5060 |
|  | Malate permease | *M. agalactiae* PG2 | 45146 | 9.75 | 145 | 5 | 9% | gi|148377751 | MAG_4890 |
|  | L-lactate dehydrogenase (L-LDH) | *M. agalactiae* PG2 | 34965 | 6.13 | 141 | 4 | 9% | gi|148377752 | MAG_4900 |
|  | Protein-export membrane protein | *M. agalactiae* PG2 | 89106 | 9.52 | 103 | 4 | 5% | gi|148377491 | MAG_2250 |
|  | ABC transporter. ATP-binding protein | *M. agalactiae* PG2 | 68597 | 9.25 | 94 | 1 | 2% | gi|148377862 | MAG_5990 |
|  | Preprotein translocase subunit SecY | *M. agalactiae* PG2 | 54823 | 9.7 | 91 | 2 | 3% | gi|148377788 | MAG_5260 |
|  | Prolipoprotein diacylglyceryl transferase | *M. agalactiae* PG2 | 37843 | 9.3 | 87 | 1 | 3% | gi|148377358 | MAG_0900 |
|  | Cation-transporting P-ATPase | *M. agalactiae* PG2 | 100451 | 8.6 | 82 | 2 | 2% | gi|148378004 | MAG_7420 |
|  | ABC transporter. ATP-binding protein | *M. agalactiae* PG2 | 67154 | 8.52 | 81 | 2 | 3% | gi|148377863 | MAG_6000 |
|  | ABC transporter ATP-binding protein | *M. agalactiae* PG2 | 80105 | 9.01 | 78 | 1 | 1% | gi|148377767 | MAG_5050 |
|  | Dihydrolipoamide dehydrogenase (E3 component of pyruvate complex) | *M. agalactiae* PG2 | 58917 | 6.36 | 72 | 7 | 7% | gi|148377364 | MAG_0960 |
|  | ABC transporter permease protein | *M. agalactiae* PG2 | 37630 | 9.89 | 67 | 1 | 2% | gi|148377769 | MAG_5070 |
|  | Hypothetical protein MAG_2680 | *M. agalactiae* PG2 | 55645 | 4.72 | 65 | 1 | 2% | gi|148377534 | MAG_2680 |
|  | Pyruvate dehydrogenase E1 component. betasubunit | *M. agalactiae* PG2 | 36129 | 5.44 | 64 | 2 | 3% | gi|148377362 | MAG_0940 |
|  | Pyruvate dehydrogenase E1 component. alphasubunit | *M. agalactiae* PG2 | 41321 | 6.13 | 62 | 2 | 5% | gi|148377361 | MAG_0930 |
|  | Putative phosphoketolase | *M. agalactiae* PG2 | 89860 | 6.07 | 56 | 3 | 3% | gi|148377390 | MAG_1230 |
|  | Oligopeptide ABC transporter system. permeaseprotein (OppC) | *M. agalactiae* PG2 | 47514 | 8.85 | 53 | 3 | 4% | gi|148377370 | MAG_1020 |
|  | DNA-directed RNA polymerase subunit beta' | *M. agalactiae* PG2 | 167709 | 6.47 | 50 | 1 | <1% | gi|148377874 | MAG_6110 |
|  | ABC transporter. permease protein | *M. agalactiae* PG2 | 71089 | 8.87 | 46 | 1 | 1% | gi|148377723 | MAG_4600 |
|  | P80. lipoprotein | *M. agalactiae* PG2 | 81102 | 9.08 | 45 | 1 | 1% | gi|148377765 | MAG_5030 |
|  | XAA-Pro aminopeptidase | *M. agalactiae* PG2 | 40001 | 5.88 | 44 | 2 | 6% | gi|148377385 | MAG_1180 |
|  | Hypothetical protein MAG_7360 | *M. agalactiae* PG2 | 53119 | 9.4 | 42 | 2 | 3% | gi|148377998 | MAG_7630 |
|  | Hypothetical protein MYPU_3820 | *M. pulmonis UAB CTIP* | 140043 | 8.49 | 31 | 3 | 1% | gi|15828853 | MYPU_3820 |
|  | Hypothetical protein | *M. hyopneumoniae* | 3320 | 11.39 | 30 | 1 | 28% | gi|187610428 |  |
|  | Hexosephosphate transport protein | *M. agalactiae* PG2 | 54592 | 9.81 | 29 | 3 | 6% | gi|148377759 | MAG_4970 |
|  |  |  |  |  |  |  |  |  |  |
| 4 | ABC transporter permease protein | *M. agalactiae* PG2 | 308764 | 8.86 | 265 | 14 | 4% | gi|148378006 | MAG_7440 |
|  | Putative phosphoketolase | *M. agalactiae* PG2 | 89860 | 6.07 | 252 | 8 | 9% | gi|148377390 | MAG_1230 |
|  | Elongation factor Tu | *M. agalactiae* PG2 | 43638 | 5.89 | 223 | 7 | 17% | gi|148377586 | MAG_3200 |
|  | ABC transporter ATP-binding protein | *M. agalactiae* PG2 | 80105 | 9.01 | 176 | 4 | 7% | gi|148377767 | MAG_5050 |
|  | L-lactate dehydrogenase (L-LDH) | *M. agalactiae* PG2 | 34965 | 6.13 | 103 | 3 | 9% | gi|148377752 | MAG_4900 |
|  | DNA-directed RNA polymerase subunit beta | *M. agalactiae* PG2 | 135072 | 5.72 | 96 | 3 | 2% | gi|148377875 | MAG_6120 |
|  | Dihydrolipoamide dehydrogenase (E3 component of pyruvate complex) | *M. agalactiae* PG2 | 58917 | 6.36 | 95 | 2 | 3% | gi|148377364 | MAG_0960 |
|  | Malate permease | *M. agalactiae* PG2 | 45146 | 9.75 | 77 | 1 | 4% | gi|148377751 | MAG_4890 |
|  | Hypothetical protein MAG_2680 | *M. agalactiae* PG2 | 55645 | 4.72 | 70 | 1 | 2% | gi|148377534 | MAG_2680 |
|  | Hypothetical protein MAG_0280 | *M. agalactiae* PG2 | 75681 | 9.03 | 66 | 1 | 1% | gi|148377296 | MAG_0280 |
|  | P80. lipoprotein | *M. agalactiae* PG2 | 81102 | 9.08 | 64 | 2 | 3% | gi|148377765 | MAG_5030 |
|  | Sugar ABC transporter permease | *M. agalactiae* PG2 | 74657 | 9.45 | 63 | 3 | 4% | gi|148377283 | MAG_0150 |
|  | Pyruvate dehydrogenase E1 component. alphasubunit | *M. agalactiae* PG2 | 41321 | 6.13 | 60 | 2 | 5% | gi|148377361 | MAG_0930 |
|  | Pyruvate dehydrogenase E1 component. betasubunit | *M. agalactiae* PG2 | 36129 | 5.44 | 51 | 2 | 3% | gi|148377362 | MAG_0940 |
|  | DNA-directed RNA polymerase subunit beta' | *M. agalactiae* PG2 | 167709 | 6.47 | 50 | 2 | 1% | gi|148377874 | MAG_6110 |
|  | Preprotein translocase subunit SecY | *M. agalactiae* PG2 | 54823 | 9.7 | 44 | 1 | 2% | gi|148377788 | MAG_5260 |
|  | Protein-export membrane protein | *M. agalactiae* PG2 | 89106 | 9.52 | 42 | 1 | 1% | gi|148377491 | MAG_2250 |
|  | Hypothetical protein MYPU_3820 | *M. pulmonis* UAB CTIP | 139998 | 8.49 | 41 | 1 | <1% | gi|15828853 | MYPU_3820 |
|  | Elongation factor G | *M. agalactiae* PG2 | 77499 | 5.47 | 37 | 1 | 1% | gi|148377855 | MAG_5920 |
|  | XAA-Pro aminopeptidase | *M. agalactiae* PG2 | 40001 | 5.88 | 35 | 1 | 3% | gi|148377385 | MAG_1180 |
|  | ABC transporter permease protein | *M. agalactiae* PG2 | 39653 | 9.72 | 34 | 1 | 3% | gi|148377768 | MAG_5060 |
|  | Glycerol ABC transporter. ATP-binding protein | *M. capricolum* subsp. *capricolum* ATCC 27343 | 47127 | 9.59 | 34 | 1 | 2% | gi|83319663 | MCAP_0454 |
|  | Putative inner membrane protein translocase component YidC | *M. agalactiae* PG2 | 83003 | 9.48 | 33 | 2 | 3% | gi|148377328 | MAG_0590 |
|  | Alanyl-tRNA synthetase | *M. agalactiae* PG2 | 100239 | 5.37 | 29 | 1 | 1% | gi|148377682 | MAG_4160 |
|  | Hypothetical protein | *M. hyopneumoniae* | ??? |  | 27 | 1 |  | gi|187610428 |  |
|  |  |  |  |  |  |  |  |  |  |
| 5 | DNA-directed RNA polymerase subunit beta' | *M. agalactiae* PG2 | 167709 | 6.47 | 1305 | 54 | 21% | gi|148377874 | MAG_6110 |
|  | P80. lipoprotein | *M. agalactiae* PG2 | 81102 | 9.08 | 106 | 4 | 5% | gi|148377765 | MAG_5030 |
|  | Elongation factor Tu | *M. agalactiae* PG2 | 43638 | 5.89 | 104 | 4 | 11% | gi|148377586 | MAG_3200 |
|  | Pyruvate dehydrogenase E1 component. betasubunit | *M. agalactiae* PG2 | 36129 | 5.44 | 97 | 2 | 3% | gi|148377362 | MAG_0940 |
|  | Hypothetical protein MAG_2680 | *M. agalactiae* PG2 | 55645 | 4.72 | 76 | 1 | 2% | gi|148377534 | MAG_2680 |
|  | ABC transporter ATP-binding protein | *M. agalactiae* PG2 | 80105 | 9.01 | 73 | 2 | 3% | gi|148377767 | MAG_5050 |
|  | Pyruvate dehydrogenase E1 component. alphasubunit | *M. agalactiae* PG2 | 41321 | 6.13 | 67 | 4 | 7% | gi|148377361 | MAG_0930 |
|  | Sugar ABC transporter permease | *M. agalactiae* PG2 | 74657 | 9.45 | 56 | 1 | 1% | gi|148377283 | MAG_0150 |
|  | Putative phosphoketolase | *M. agalactiae* PG2 | 89860 | 6.07 | 53 | 2 | 2% | gi|148377390 | MAG_1230 |
|  | Preprotein translocase subunit SecY | *M. agalactiae* PG2 | 54823 | 9.7 | 42 | 2 | 3% | gi|148377788 | MAG_5260 |
|  | Malate permease | *M. agalactiae* PG2 | 45146 | 9.75 | 41 | 2 | 6% | gi|148377751 | MAG_4890 |
|  | XAA-Pro aminopeptidase | *M. agalactiae* PG2 | 40001 | 5.88 | 36 | 2 | 5% | gi|148377385 | MAG_1180 |
|  | Dihydrolipoamide dehydrogenase (E3 component of pyruvate complex) | *M. agalactiae* PG2 | 58917 | 6.36 | 27 | 2 | 3% | gi|148377364 | MAG_0960 |
|  | L-lactate dehydrogenase (L-LDH) | *M. agalactiae* PG2 | 34965 | 6.13 | 25 | 3 | 9% | gi|148377752 | MAG_4900 |
|  |  |  |  |  |  |  |  |  |  |
| 6 | DNA-directed RNA polymerase subunit beta | *M. agalactiae* PG2 | 135072 | 5.13 | 1663 | 70 | 28% | gi|148377875 | MAG_6120 |
|  | Elongation factor Tu | *M. agalactiae* PG2 | 43638 | 5.89 | 119 | 2 | 6% | gi|148377586 | MAG_3200 |
|  | ABC transporter ATP-binding protein | *M. agalactiae* PG2 | 80105 | 9.01 | 117 | 2 | 3% | gi|148377767 | MAG_5050 |
|  | DNA-directed RNA polymerase subunit beta' | *M. agalactiae* PG2 | 167709 | 6.47 | 102 | 4 | 2% | gi|148377874 | MAG_6110 |
|  | Dihydrolipoamide dehydrogenase (E3 component of pyruvate complex) | *M. agalactiae* PG2 | 58917 | 6.36 | 88 | 2 | 3% | gi|148377364 | MAG_0960 |
|  | Pyruvate dehydrogenase E1 component. betasubunit | *M. agalactiae* PG2 | 36129 | 5.44 | 83 | 3 | 3% | gi|148377362 | MAG_0940 |
|  | L-lactate dehydrogenase (L-LDH) | *M. agalactiae* PG2 | 34965 | 6.13 | 68 | 1 | 2% | gi|148377752 | MAG_4900 |
|  | P80. lipoprotein | *M. agalactiae* PG2 | 81102 | 9.08 | 64 | 1 | 1% | gi|148377765 | MAG_5030 |
|  | Pyruvate dehydrogenase E1 component. alphasubunit | *M. agalactiae* PG2 | 41321 | 6.13 | 57 | 2 | 5% | gi|148377361 | MAG_0930 |
|  | Preprotein translocase subunit SecY | *M. agalactiae* PG2 | 54823 | 9.7 | 38 | 1 | 2% | gi|148377788 | MAG_5260 |
|  | XAA-Pro aminopeptidase | *M. agalactiae* PG2 | 40001 | 5.88 | 34 | 1 | 2% | gi|148377385 | MAG_1180 |
|  |  |  |  |  |  |  |  |  |  |
| 9 | Hypothetical protein MAG_1000 | *M. agalactiae* PG2 | 109727 | 8.66 | 1984 | 76 | 38% | gi|148377368 | MAG_1000 |
|  | Hypothetical protein MAG_2680 | *M. agalactiae* PG2 | 55645 | 4.72 | 444 | 14 | 32% | gi|148377534 | MAG_2680 |
|  | Alanyl-tRNA synthetase | *M. agalactiae* PG2 | 100239 | 5.37 | 165 | 3 | 4% | gi|148377682 | MAG_4160 |
|  | Elongation factor Tu | *M. agalactiae* PG2 | 43638 | 5.89 | 161 | 4 | 8% | gi|148377586 | MAG_3200 |
|  | Isoleucyl-tRNA synthetase | *M. agalactiae* PG2 | 103127 | 6.19 | 134 | 7 | 6% | gi|148377486 | MAG_2200 |
|  | Preprotein translocase subunit SecA | *M. agalactiae* PG2 | 96419 | 5.16 | 72 | 2 | 2% | gi|148377539 | MAG_2730 |
|  | Pyruvate dehydrogenase E1 component. betasubunit | *M. agalactiae* PG2 | 36129 | 5.44 | 62 | 1 | 3% | gi|148377362 | MAG_0940 |
|  | Pyruvate dehydrogenase E1 component. alphasubunit | *M. agalactiae* PG2 | 41321 | 6.13 | 61 | 2 | 5% | gi|148377361 | MAG_0930 |
|  | Oligopeptide ABC transporter. substrate-bindingprotein (OppA). lipoprotein | *M. agalactiae* PG2 | 113019 | 6.24 | 55 | 1 | 1% | gi|148377306 | MAG_0380 |
|  | DNA gyrase subunit A | *M. agalactiae* PG2 | 103247 | 5.33 | 43 | 1 | 1% | gi|148377826 | MAG_5630 |
|  | XAA-Pro aminopeptidase | *M. agalactiae* PG2 | 40001 | 5.88 | 43 | 1 | 3% | gi|148377385 | MAG_1180 |
|  | Hydrolase (HAD family) | *M. capricolum* subsp*. capricolum* ATCC 27343 | 5768 | 5.25 | 37 | 1 | 21% | gi|963067 |  |
|  |  |  |  |  |  |  |  |  |  |
| 10 | Hypothetical protein MAG_1000 | *M. agalactiae* PG2 | 109727 | 8.66 | 221 | 10 | 9% | gi|148377368 | MAG_1000 |
|  | Preprotein translocase subunit SecA | *M. agalactiae* PG2 | 96419 | 5.16 | 158 | 6 | 7% | gi|148377539 | MAG_2730 |
|  | Alanyl-tRNA synthetase | *M. agalactiae* PG2 | 100239 | 5.37 | 147 | 3 | 3% | gi|148377682 | MAG_4160 |
|  | Modification (methylase) protein of type irestriction-modification system HsdM | *M. agalactiae* PG2 | 103753 | 5.07 | 111 | 4 | 5% | gi|148377836 | MAG_5730 |
|  | Modification (methylase) protein of type irestriction-modification system | *M. agalactiae* PG2 | 103659 | 5.02 | 111 | 4 | 5% | gi|148377828 | MAG_5650 |
|  | Putative phosphoketolase | *M. agalactiae* PG2 | 89860 | 6.07 | 92 | 1 | 2% | gi|148377390 | MAG_1230 |
|  | Valyl-tRNA synthetase | *M. agalactiae* PG2 | 97424 | 8.78 | 87 | 2 | 2% | gi|148377404 | MAG_1370 |
|  | Elongation factor Tu | *M. agalactiae* PG2 | 43638 | 5.89 | 84 | 1 | 3% | gi|148377586 | MAG_3200 |
|  | ABC transporter ATP-binding protein | *M. agalactiae* PG2 | 80105 | 9.01 | 58 | 1 | 1% | gi|148377767 | MAG_5050 |
|  | Pyruvate dehydrogenase E1 component. alphasubunit | *M. agalactiae* PG2 | 41321 | 6.13 | 54 | 1 | 3% | gi|148377361 | MAG_0930 |
|  | Glycerol ABC transporter. ATP-binding protein | *M. capricolum* subsp. *capricolum* ATCC 27343 | 47127 | 9.59 | 37 | 1 | 2% | gi|83319663 | MCAP_0454 |
|  | Dihydrolipoamide dehydrogenase (E3 component of pyruvate complex) | *M. agalactiae* PG2 | 58917 | 6.36 | 31 | 1 | 1% | gi|148377364 | MAG_0960 |
|  |  |  |  |  |  |  |  |  |  |
| 11 | Putative phosphoketolase | *M. agalactiae* PG2 | 89860 | 6.07 | 1733 | 48 | 31% | gi|148377390 | MAG_1230 |
|  | P80. lipoprotein | *M. agalactiae* PG2 | 81102 | 9.08 | 292 | 8 | 9% | gi|148377765 | MAG_5030 |
|  | Oligopeptide ABC transporter. ATP-bindingprotein (OppF) | *M. agalactiae* PG2 | 93878 | 8.99 | 226 | 11 | 15% | gi|148377372 | MAG_1040 |
|  | Elongation factor Tu | *M. agalactiae* PG2 | 43638 | 5.89 | 211 | 4 | 9% | gi|148377586 | MAG_3200 |
|  | ABC transporter ATP-binding protein | *M. agalactiae* PG2 | 80105 | 9.01 | 112 | 2 | 3% | gi|148377767 | MAG_5050 |
|  | Leucyl-tRNA synthetase (leucine-tRNA ligase) | *M. agalactiae* PG2 | 91191 | 7.51 | 94 | 5 | 5% | gi|148377630 | MAG_3640 |
|  | Elongation factor G | *M. agalactiae* PG2 | 77499 | 5.47 | 85 | 3 | 3% | gi|148377855 | MAG_5920 |
|  | Pyruvate dehydrogenase E1 component. alphasubunit | *M. agalactiae* PG2 | 41321 | 6.13 | 81 | 3 | 7% | gi|148377361 | MAG_0930 |
|  | Endopeptidase O | *M. agalactiae* PG2 | 75007 | 8.5 | 73 | 1 | 1% | gi|148377634 | MAG_3680 |
|  | Ribonuclease R | *M. agalactiae* PG2 | 84293 | 6.36 | 58 | 1 | 1% | gi|148377474 | MAG_2080 |
|  | Hypothetical protein MAG_1000 | *M. agalactiae* PG2 | 109727 | 8.66 | 57 | 1 | 1% | gi|148377368 | MAG_1000 |
|  | Hypothetical protein MAG_6230 | *M. agalactiae* PG2 | 82041 | 6.18 | 45 | 2 | 2% | gi|148377886 | MAG_6230 |
|  | Glycerol ABC transporter. ATP-binding protein | *M. capricolum* subsp. *capricolum* ATCC 27343 | 47127 | 9.59 | 38 | 1 | 2% | gi|83319663 | MCAP_0454 |
|  |  |  |  |  |  |  |  |  |  |
| 12 | P80. lipoprotein | *M. agalactiae* PG2 | 81102 | 9.08 | 1144 | 49 | 30% | gi|148377765 | MAG_5030 |
|  | Putative phosphoketolase | *M. agalactiae* PG2 | 89860 | 6.07 | 610 | 17 | 12% | gi|148377390 | MAG_1230 |
|  | Hypothetical protein MAG_6230 | *M. agalactiae* PG2 | 82041 | 6.18 | 339 | 8 | 14% | gi|148377886 | MAG_6230 |
|  | ABC transporter ATP-binding protein | *M. agalactiae* PG2 | 80105 | 9.01 | 135 | 3 | 6% | gi|148377767 | MAG_5050 |
|  | Hypothetical protein MAG_1970 | *M. agalactiae* PG2 | 82243 | 9.03 | 133 | 3 | 5% | gi|148377463 | MAG_1970 |
|  | ClpB | *M. agalactiae* PG2 | 82234 | 5.98 | 126 | 2 | 3% | gi|148377887 | MAG_6240 |
|  | Elongation factor G | *M. agalactiae* PG2 | 77499 | 5.47 | 125 | 4 | 5% | gi|148377855 | MAG_5920 |
|  | Elongation factor Tu | *M. agalactiae* PG2 | 43638 | 5.89 | 122 | 3 | 8% | gi|148377586 | MAG_3200 |
|  | Endopeptidase O | *M. agalactiae* PG2 | 75007 | 8.5 | 67 | 1 | 1% | gi|148377634 | MAG_3680 |
|  | Pyruvate dehydrogenase E1 component. alphasubunit | *M. agalactiae* PG2 | 41321 | 6.13 | 53 | 1 | 3% | gi|148377361 | MAG_0930 |
|  | Glycerol ABC transporter. ATP-binding protein | *M. capricolum* subsp. *capricolum* ATCC 27343 | 47127 | 9.59 | 42 | 1 | 2% | gi|83319663 | MCAP_0454 |
|  | Pyruvate dehydrogenase E1 component. betasubunit | *M. agalactiae* PG2 | 36129 | 5.44 | 42 | 1 | 3% | gi|148377362 | MAG_0940 |
|  | leucyl-tRNA synthetase (leucine-tRNA ligase) | *M. agalactiae* PG2 | 91191 | 7.51 | 29 | 2 | 2% | gi|148377630 | MAG_3640 |
|  |  |  |  |  |  |  |  |  |  |
| 13 | P80. lipoprotein | *M. agalactiae* PG2 | 81102 | 9.08 | 1913 | 51 | 48% | gi|148377765 | MAG_5030 |
|  | ABC transporter ATP-binding protein | *M. agalactiae* PG2 | 80105 | 9.01 | 600 | 18 | 23% | gi|148377767 | MAG_5050 |
|  | Hypothetical protein MAG_1970 | *M. agalactiae* PG2 | 82243 | 9.03 | 424 | 15 | 18% | gi|148377463 | MAG_1970 |
|  | ClpB | *M. agalactiae* PG2 | 82234 | 5.98 | 101 | 4 | 6% | gi|148377887 | MAG_6240 |
|  | Cell division protein ftsH-like protein | *M. agalactiae* PG2 | 74857 | 5.94 | 101 | 3 | 6% | gi|148378007 | MAG_7450 |
|  | DNA gyrase subunit B | *M. agalactiae* PG2 | 73507 | 5.85 | 92 | 2 | 2% | gi|148377999 | MAG_7370 |
|  | Hypothetical protein MAG_6230 | *M. agalactiae* PG2 | 82041 | 6.18 | 84 | 1 | 2% | gi|148377886 | MAG_6230 |
|  | Elongation factor G | *M. agalactiae* PG2 | 77499 | 5.47 | 59 | 1 | 1% | gi|148377855 | MAG_5920 |
|  | Glycerol ABC transporter. ATP-binding protein | *M. capricolum* subsp. *capricolum* ATCC 27343 | 47127 | 9.59 | 47 | 1 | 2% | gi|83319663 | MCAP_0454 |
|  | Endopeptidase O | *M. agalactiae* PG2 | 75007 | 8.5 | 47 | 4 | 5% | gi|148377634 | MAG_3680 |
|  | Pyruvate dehydrogenase E1 component. alphasubunit | *M. agalactiae* PG2 | 41321 | 6.13 | 44 | 1 | 3% | gi|148377361 | MAG_0930 |
|  | Putative phosphoketolase | *M. agalactiae* PG2 | 89860 | 6.07 | 43 | 1 | 1% | gi|148377390 | MAG_1230 |
|  | Dihydrolipoamide dehydrogenase (E3 component of pyruvate complex) | *M. agalactiae* PG2 | 58917 | 6.36 | 43 | 1 | 1% | gi|148377364 | MAG_0960 |
|  |  |  |  |  |  |  |  |  |  |
| 14 | 5'nucleotidase | *M. agalactiae* PG2 | 76195 | 8.36 | 1167 | 35 | 34% | gi|148377854 | MAG_5910 |
|  | P80. lipoprotein | *M. agalactiae* PG2 | 81102 | 9.08 | 934 | 29 | 33% | gi|148377765 | MAG_5030 |
|  | Putative inner membrane protein translocase component YidC | *M. agalactiae* PG2 | 83003 | 9.48 | 617 | 14 | 21% | gi|148377328 | MAG_0590 |
|  | Molecular chaperone DnaK | *M. agalactiae* PG2 | 65263 | 4.95 | 459 | 11 | 16% | gi|148377413 | MAG_1460 |
|  | Endopeptidase O | *M. agalactiae* PG2 | 75007 | 8.5 | 394 | 12 | 15% | gi|148377634 | MAG_3680 |
|  | ABC transporter ATP-binding protein | *M. agalactiae* PG2 | 80105 | 9.01 | 310 | 9 | 14% | gi|148377767 | MAG_5050 |
|  | Cell division protein ftsH-like protein | *M. agalactiae* PG2 | 74857 | 5.94 | 204 | 8 | 13% | gi|148378007 | MAG_7450 |
|  | Excinuclease ABC subunit B | *M. agalactiae* PG2 | 77404 | 5.78 | 94 | 3 | 4% | gi|148377644 | MAG_3780 |
|  | Pyruvate dehydrogenase E1 component. alphasubunit | *M. agalactiae* PG2 | 41321 | 6.13 | 58 | 2 | 5% | gi|148377361 | MAG_0930 |
|  | Hypothetical protein MAG_1970 | *M. agalactiae* PG2 | 82243 | 9.03 | 54 | 1 | 1% | gi|148377463 | MAG_1970 |
|  | DNA gyrase subunit B | *M. agalactiae* PG2 | 73507 | 5.85 | 52 | 1 | 1% | gi|148377999 | MAG_7370 |
|  | DNA polymerase III subunit gamma and tau | *M. agalactiae* PG2 | 70644 | 5.25 | 51 | 2 | 3% | gi|148377949 | MAG_6870 |
|  | Hypothetical protein MAG_4720 | *M. agalactiae* PG2 | 86074 | 8.82 | 47 | 2 | 2% | gi|148377735 | MAG_4720 |
|  | Glycerol ABC transporter. ATP-binding protein | *M. capricolum* subsp. *capricolum* ATCC 27343 | 47127 | 9.59 | 44 | 2 | 6% | gi|83319663 | MCAP_0454 |
|  | Putative transmembrane protein | *M. agalactiae* PG2 | 83256 | 9.06 | 43 | 1 | 1% | gi|148377558 | MAG_2920 |
|  |  |  |  |  |  |  |  |  |  |
| 15 | Putative inner membrane protein translocase component YidC | *M. agalactiae* PG2 | 83003 | 9.48 | 768 | 23 | 23% | gi|148377328 | MAG_0590 |
|  | 5'nucleotidase | *M. agalactiae* PG2 | 76195 | 8.36 | 508 | 17 | 17% | gi|148377854 | MAG_5910 |
|  | P80. lipoprotein | *M. agalactiae* PG2 | 81102 | 9.08 | 421 | 13 | 19% | gi|148377765 | MAG_5030 |
|  | Hypothetical protein MAG_6520 | *M. agalactiae* PG2 | 70087 | 8.58 | 376 | 12 | 17% | gi|148377915 | MAG_6520 |
|  | Hypothetical protein MAG_1210 | *M. agalactiae* PG2 | 70044 | 5.71 | 234 | 8 | 10% | gi|148377388 | MAG_1210 |
|  | ABC transporter ATP-binding protein | *M. agalactiae* PG2 | 61006 | 5.24 | 200 | 9 | 11% | gi|148377859 | MAG_5960 |
|  | DNA ligase | *M. agalactiae* PG2 | 74509 | 6.12 | 106 | 3 | 5% | gi|148377548 | MAG_2820 |
|  | Endopeptidase O | *M. agalactiae* PG2 | 75007 | 8.5 | 79 | 3 | 2% | gi|148377634 | MAG_3680 |
|  | Dihydrolipoamide dehydrogenase (E3 component of pyruvate complex) | *M. agalactiae* PG2 | 58917 | 6.36 | 43 | 4 | 5% | gi|148377364 | MAG_0960 |
|  | Pyruvate dehydrogenase E1 component. alphasubunit | *M. agalactiae* PG2 | 41321 | 6.13 | 42 | 1 | 3% | gi|148377361 | MAG_0930 |
|  | Hypothetical protein MAG_2360 | *M. agalactiae* PG2 | 75426 | 6.19 | 33 | 1 | 1% | gi|148377502 | MAG_2360 |
|  | Topoisomerase IV subunit B | *M. agalactiae* PG2 | 72252 | 8.07 | 31 | 2 | 2% | gi|148377448 | MAG_1820 |
|  |  |  |  |  |  |  |  |  |  |
| 16 | Hypothetical protein MAG_2220 | *M. agalactiae* PG2 | 69573 | 8.78 | 1512 | 63 | 32% | gi|148377488 | MAG_2220 |
|  | ABC transporter ATP-binding protein | *M. agalactiae* PG2 | 61006 | 5.24 | 394 | 18 | 19% | gi|148377859 | MAG_5960 |
|  | Hypothetical protein MAG_6520 | *M. agalactiae* PG2 | 70087 | 8.58 | 365 | 13 | 13% | gi|148377915 | MAG_6520 |
|  | Threonyl-tRNA synthetase | *M. agalactiae* PG2 | 67728 | 8.77 | 146 | 3 | 3% | gi|148377610 | MAG_3440 |
|  | Putative inner membrane protein translocase component YidC | *M. agalactiae* PG2 | 83003 | 9.48 | 101 | 3 | 5% | gi|148377328 | MAG_0590 |
|  | P80. lipoprotein | *M. agalactiae* PG2 | 81102 | 9.08 | 97 | 4 | 5% | gi|148377765 | MAG_5030 |
|  | Type III restriction-modification system: methylase | *M. agalactiae* PG2 | 66381 | 6.17 | 96 | 7 | 8% | gi|148377420 | MAG_1530 |
|  | Trigger factor | *M. agalactiae* PG2 | 56779 | 5.15 | 86 | 6 | 9% | gi|148377421 | MAG_1540 |
|  | Pyruvate dehydrogenase E1 component. alphasubunit | *M. agalactiae* PG2 | 41321 | 6.13 | 58 | 2 | 5% | gi|148377361 | MAG_0930 |
|  | Dihydrolipoamide dehydrogenase (E3 component of pyruvate complex) | *M. agalactiae* PG2 | 58917 | 6.36 | 43 | 2 | 3% | gi|148377364 | MAG_0960 |
|  | Hypothetical protein MAG_3240 | *M. agalactiae* PG2 | 72359 | 8.02 | 36 | 2 | 3% | gi|148377590 | MAG_3240 |
|  | Hypothetical protein MAG_1210 | *M. agalactiae* PG2 | 70044 | 5.71 | 36 | 2 | 2% | gi|148377388 | MAG_1210 |
|  | Protein-export membrane protein | *M. agalactiae* PG2 | 89106 | 9.52 | 34 | 1 | 1% | gi|148377491 | MAG_2250 |
|  | Hypothetical protein MAG_2340 | *M. agalactiae* PG2 | 66023 | 7.56 | 28 | 5 | 1% | gi|148377500 | MAG_2340 |
|  | Hypothetical protein MAG_4410 | *M. agalactiae* PG2 | 60649 | 5.12 | 26 | 1 | 3% | gi|148377704 | MAG_4410 |
|  |  |  |  |  |  |  |  |  |  |
| 17 | Hypothetical protein MAG_2220 | *M. agalactiae* PG2 | 69573 | 8.78 | 300 | 12 | 13% | gi|148377488 | MAG_2220 |
|  | Threonyl-tRNA synthetase | *M. agalactiae* PG2 | 67728 | 8.77 | 135 | 8 | 8% | gi|148377610 | MAG_3440 |
|  | Hypothetical protein MAG_0280 | *M. agalactiae* PG2 | 75681 | 9.03 | 55 | 1 | 1% | gi|148377296 | MAG_0280 |
|  | P80. lipoprotein | *M. agalactiae* PG2 | 81102 | 9.08 | 49 | 2 | 3% | gi|148377765 | MAG_5030 |
|  | Type III restriction-modification system: methylase | *M. agalactiae* PG2 | 66381 | 6.17 | 40 | 1 | 1% | gi|148377420 | MAG_1530 |
|  | Elongation factor Tu | *M. agalactiae* PG2 | 43638 | 5.89 | 26 | 1 | 2% | gi|148377586 | MAG_3200 |
|  |  |  |  |  |  |  |  |  |  |
| 18 | ABC transporter. ATP-binding protein. P59 | *M. agalactiae* PG2 | 58997 | 6.43 | 852 | 29 | 18% | gi|148377282 | MAG_0140 |
|  | F0F1 ATP synthase subunit alpha | *M. agalactiae* PG2 | 58096 | 5.95 | 126 | 3 | 7% | gi|148377620 | MAG_3540 |
|  | Phosphoglyceromutase | *M. agalactiae* PG2 | 55761 | 5.87 | 126 | 2 | 2% | gi|148377951 | MAG_6890 |
|  | CTP synthetase | *M. agalactiae* PG2 | 60669 | 7.65 | 122 | 4 | 7% | gi|148377485 | MAG_2190 |
|  | Hypothetical protein MAG_2220 | *M. agalactiae* PG2 | 69573 | 8.78 | 84 | 4 | 7% | gi|148377488 | MAG_2220 |
|  | Pyruvate kinase | *M. agalactiae* PG2 | 53469 | 5.81 | 72 | 2 | 3% | gi|148377411 | MAG_1440 |
|  | Lysyl-tRNA synthetase | *M. agalactiae* PG2 | 56530 | 5.56 | 53 | 1 | 2% | gi|148377884 | MAG_6210 |
|  | Spermidine/putrescine ABC transporter ATP-binding protein | *M. agalactiae* PG2 | 54920 | 6.15 | 53 | 1 | 1% | gi|148377392 | MAG_1250 |
|  | Preprotein translocase subunit SecY | *M. agalactiae* PG2 | 54823 | 9.7 | 48 | 1 | 2% | gi|148377788 | MAG_5260 |
|  | P80. lipoprotein | *M. agalactiae* PG2 | 81102 | 9.08 | 38 | 2 | 3% | gi|148377765 | MAG_5030 |
|  | ABC transporter. ATP-binding protein | *M. agalactiae* PG2 | 67154 | 8.52 | 37 | 2 | 3% | gi|148377863 | MAG_6000 |
|  | Elongation factor Tu | *M. agalactiae* PG2 | 43638 | 5.89 | 31 | 1 | 2% | gi|148377586 | MAG_3200 |
|  | Type III restriction-modification system: methylase | *M. agalactiae* PG2 | 66381 | 6.17 | 29 | 1 | 1% | gi|148377420 | MAG_1530 |
|  | XAA-Pro aminopeptidase | *M. agalactiae* PG2 | 40001 | 5.88 | 25 | 1 | 2% | gi|148377385 | MAG_1180 |
|  |  |  |  |  |  |  |  |  |  |
| 19 | Pyruvate kinase | *M. agalactiae* PG2 | 53469 | 5.81 | 751 | 24 | 35% | gi|148377411 | MAG_1440 |
|  | Lipoprotein MAG_5080 | *M. agalactiae* PG2 | 59415 | 6.77 | 608 | 30 | 27% | gi|148377770 | MAG_5080 |
|  | Lysyl-tRNA synthetase | *M. agalactiae* PG2 | 56530 | 5.56 | 363 | 13 | 25% | gi|148377884 | MAG_6210 |
|  | Hypothetical protein MAG_1430 | *M. agalactiae* PG2 | 56379 | 8.52 | 330 | 8 | 15% | gi|148377410 | MAG_1430 |
|  | ABC transporter. ATP-binding protein | *M. agalactiae* PG2 | 67154 | 8.52 | 241 | 4 | 8% | gi|148377863 | MAG_6000 |
|  | Glutamyl-tRNA synthetase | *M. agalactiae* PG2 | 53870 | 6.08 | 239 | 9 | 17% | gi|148377841 | MAG_5780 |
|  | Methionyl-tRNA synthetase | *M. agalactiae* PG2 | 60263 | 5.89 | 207 | 3 | 7% | gi|148377472 | MAG_2060 |
|  | Prolyl-tRNA synthetase | *M. agalactiae* PG2 | 55488 | 9.24 | 198 | 5 | 11% | gi|148377386 | MAG_1190 |
|  | ABC transporter. ATP-binding protein. P59 | *M. agalactiae* PG2 | 58997 | 6.43 | 185 | 8 | 16% | gi|148377282 | MAG_0140 |
|  | Elongation factor Tu | *M. agalactiae* PG2 | 43638 | 5.89 | 97 | 3 | 6% | gi|148377586 | MAG_3200 |
|  | Hypothetical protein MAG_2220 | *M. agalactiae* PG2 | 69573 | 8.78 | 89 | 3 | 5% | gi|148377488 | MAG_2220 |
|  | Phosphoglyceromutase | *M. agalactiae* PG2 | 55761 | 5.87 | 89 | 1 | 2% | gi|148377951 | MAG_6890 |
|  | Pyruvate dehydrogenase E1 component. alphasubunit | *M. agalactiae* PG2 | 41321 | 6.13 | 56 | 1 | 3% | gi|148377361 | MAG_0930 |
|  | Spermidine/putrescine ABC transporter ATP-binding protein | *M. agalactiae* PG2 | 54920 | 6.15 | 53 | 1 | 1% | gi|148377392 | MAG_1250 |
|  |  |  |  |  |  |  |  |  |  |
| 20 | Pyruvate kinase | *M. agalactiae* PG2 | 53469 | 5.81 | 243 | 11 | 20% | gi|148377411 | MAG_1440 |
|  | Glycerol kinase | *M. agalactiae* PG2 | 56853 | 5.95 | 223 | 7 | 9% | gi|148377710 | MAG_4470 |
|  | Hypothetical protein MAG_1430 | *M. agalactiae* PG2 | 56379 | 8.52 | 153 | 2 | 5% | gi|148377410 | MAG_1430 |
|  | Aminopeptidase (leucine aminopeptidase) | *M. agalactiae* PG2 | 50393 | 5.63 | 91 | 2 | 2% | gi|148377964 | MAG_7020 |
|  | Elongation factor Tu | *M. agalactiae* PG2 | 43638 | 5.89 | 80 | 2 | 5% | gi|148377586 | MAG_3200 |
|  | Lysyl-tRNA synthetase | *M. agalactiae* PG2 | 56530 | 5.56 | 80 | 4 | 5% | gi|148377884 | MAG_6210 |
|  | Replicative DNA helicase | *M. agalactiae* PG2 | 56206 | 6.66 | 71 | 1 | 1% | gi|148377504 | MAG_2380 |
|  | Glutamyl-tRNA synthetase | *M. agalactiae* PG2 | 53870 | 6.08 | 48 | 1 | 1% | gi|148377841 | MAG_5780 |
|  | P80. lipoprotein | *M. agalactiae* PG2 | 81102 | 9.08 | 46 | 2 | 3% | gi|148377765 | MAG_5030 |
|  | Lipoprotein. MAG_5080 | *M. agalactiae* PG2 | 59415 | 6.77 | 45 | 4 | 6% | gi|148377770 | MAG_5080 |
|  | XAA-Pro aminopeptidase | *M. agalactiae* PG2 | 40001 | 5.88 | 33 | 1 | 2% | gi|148377385 | MAG_1180 |
|  |  |  |  |  |  |  |  |  |  |
| 21 | Hypothetical protein MAG_4460 | *M. agalactiae* PG2 | 52483 | 8.7 | 658 | 30 | 26% | gi|148377709 | MAG_4460 |
|  | P48. lipoprotein | *M. agalactiae* PG2 | 51232 | 8.39 | 481 | 17 | 30% | gi|148377280 | MAG_0120 |
|  | F0F1 ATP synthase subunit beta | *M. agalactiae* PG2 | 53330 | 5.28 | 371 | 9 | 17% | gi|148377622 | MAG_3560 |
|  | Aminopeptidase (leucine aminopeptidase) | *M. agalactiae* PG2 | 50393 | 5.63 | 367 | 13 | 21% | gi|148377964 | MAG_7020 |
|  | Glycerol kinase | *M. agalactiae* PG2 | 56853 | 5.95 | 238 | 6 | 11% | gi|148377710 | MAG_4470 |
|  | NADH oxidase (NOXASE) | *M. agalactiae* PG2 | 49980 | 6.09 | 235 | 9 | 17% | gi|148377529 | MAG_2630 |
|  | Variable surface lipoprotein V | *M. agalactiae* PG2 | 37362 | 9.25 | 133 | 6 | 12% | gi|148377967 | MAG_7050 |
|  | GTPase ObgE | *M. agalactiae* PG2 | 46867 | 5.66 | 116 | 4 | 7% | gi|148377771 | MAG_5090 |
|  | Signal recognition particle protein | *M. agalactiae* PG2 | 50358 | 9.17 | 95 | 2 | 4% | gi|148377845 | MAG_5820 |
|  | DNA recombination protein | *M. agalactiae* PG2 | 55552 | 6.27 | 80 | 3 | 7% | gi|148377514 | MAG_2480 |
|  | P80. lipoprotein | *M. agalactiae* PG2 | 81102 | 9.08 | 54 | 2 | 2% | gi|148377765 | MAG_5030 |
|  | Hypothetical protein MAG_1430 | *M. agalactiae* PG2 | 56379 | 8.52 | 54 | 1 | 2% | gi|148377410 | MAG_1430 |
|  | Pyruvate dehydrogenase E1 component. alphasubunit | *M. agalactiae* PG2 | 41321 | 6.13 | 50 | 1 | 3% | gi|148377361 | MAG_0930 |
|  | Glycyl-tRNA synthetase | *M. agalactiae* PG2 | 53051 | 6.02 | 44 | 1 | 2% | gi|148377533 | MAG_2670 |
|  | Hypothetical protein MAG_1670 | *M. agalactiae* PG2 | 53061 | 8.58 | 36 | 2 | 4% | gi|148377434 | MAG_1670 |
|  | Hypothetical protein MAG_1220 | *M. agalactiae* PG2 | 26491 | 8.69 | 30 | 1 | 3% | gi|148377389 | MAG_1220 |
|  | Glutamyl-tRNA synthetase | *M. agalactiae* PG2 | 53870 | 6.08 | 26 | 1 | 1% | gi|148377841 | MAG_5780 |
|  |  |  |  |  |  |  |  |  |  |
| 22 | P48. lipoprotein | *M. agalactiae* PG2 | 51232 | 8.39 | 1501 | 47 | 57% | gi|148377280 | MAG_0120 |
|  | Lipoprotein. MAG_1980 | *M. agalactiae* PG2 | 53788 | 8.98 | 176 | 8 | 20% | gi|148377464 | MAG_1980 |
|  | Mg^2+^ transport protein (MGTE) | *M. agalactiae* PG2 | 54236 | 4.74 | 149 | 3 | 7% | gi|148377573 | MAG_3070 |
|  | Hypothetical protein MAG_4460 | *M. agalactiae* PG2 | 52483 | 8.7 | 138 | 6 | 13% | gi|148377709 | MAG_4460 |
|  | NADH oxidase (NOXASE) | *M. agalactiae* PG2 | 49980 | 6.09 | 112 | 3 | 9% | gi|148377529 | MAG_2630 |
|  | Aminopeptidase (leucine aminopeptidase) | *M. agalactiae* PG2 | 50393 | 5.63 | 83 | 1 | 2% | gi|148377964 | MAG_7020 |
|  | XAA-Pro aminopeptidase | *M. agalactiae* PG2 | 40001 | 5.88 | 72 | 2 | 5% | gi|148377385 | MAG_1180 |
|  | Pyruvate dehydrogenase E1 component. alphasubunit | *M. agalactiae* PG2 | 41321 | 6.13 | 52 | 1 | 3% | gi|148377361 | MAG_0930 |
|  | P80. lipoprotein | *M. agalactiae* PG2 | 81102 | 9.08 | 47 | 3 | 3% | gi|148377765 | MAG_5030 |
|  | Signal recognition particle protein | *M. agalactiae* PG2 | 50358 | 9.17 | 43 | 1 | 2% | gi|148377845 | MAG_5820 |
|  | PhosphoPyruvate hydratase | *M. agalactiae* PG2 | 49710 | 5.43 | 32 | 2 | 3% | gi|148377585 | MAG_3190 |
|  | Seryl-tRNA synthetase | *M. agalactiae* PG2 | 48281 | 5.62 | 32 | 2 | 4% | gi|148377324 | MAG_0560 |
|  |  |  |  |  |  |  |  |  |  |
| 23 | Elongation factor Tu | *M. agalactiae* PG2 | 43638 | 5.89 | 1003 | 23 | 33% | gi|148377586 | MAG_3200 |
|  | P48. lipoprotein | *M. agalactiae* PG2 | 51232 | 8.39 | 997 | 28 | 49% | gi|148377280 | MAG_0120 |
|  | XAA-Pro aminopeptidase | *M. agalactiae* PG2 | 40001 | 5.88 | 407 | 15 | 28% | gi|148377385 | MAG_1180 |
|  | Phosphopentomutase | *M. agalactiae* PG2 | 43893 | 5.48 | 336 | 7 | 18% | gi|148377546 | MAG_2800 |
|  | Alkylphosphonate ABC transporter substrate-binding protein | *M. agalactiae* PG2 | 49744 | 6.99 | 272 | 6 | 12% | gi|148377535 | MAG_2690 |
|  | Phosphoglycerate kinase | *M. agalactiae* PG2 | 42781 | 5.79 | 139 | 4 | 10% | gi|148377849 | MAG_5860 |
|  | Hypothetical protein MAG_1810 | *M. agalactiae* PG2 | 52991 | 9.16 | 134 | 2 | 3% | gi|148377447 | MAG_1810 |
|  | Amidase | *M. agalactiae* PG2 | 48487 | 6.29 | 121 | 1 | 3% | gi|148377979 | MAG_7170 |
|  | Lipoprotein. MAG_1980 | *M. agalactiae* PG2 | 53788 | 8.98 | 67 | 2 | 4% | gi|148377464 | MAG_1980 |
|  | Hypothetical protein MAG_4460 | *M. agalactiae* PG2 | 52483 | 8.7 | 63 | 1 | 2% | gi|148377709 | MAG_4460 |
|  | P80. lipoprotein | *M. agalactiae* PG2 | 81102 | 9.08 | 49 | 3 | 4% | gi|148377765 | MAG_5030 |
|  | Pyruvate dehydrogenase E1 component. alphasubunit | *M. agalactiae* PG2 | 41321 | 6.13 | 42 | 1 | 3% | gi|148377361 | MAG_0930 |
|  | Asparaginyl-tRNA synthetase | *M. agalactiae* PG2 | 51629 | 7.25 | 33 | 2 | 4% | gi|148377568 | MAG_3020 |
|  |  |  |  |  |  |  |  |  |  |
| 24 | Elongation factor Tu | *M. agalactiae* PG2 | 43638 | 5.89 | 2042 | 45 | 46% | gi|148377586 | MAG_3200 |
|  | Phosphoglycerate kinase | *M. agalactiae* PG2 | 42781 | 5.79 | 441 | 19 | 25% | gi|148377849 | MAG_5860 |
|  | Hypothetical protein MAG_5040 | *M. agalactiae* PG2 | 44828 | 8.46 | 401 | 14 | 26% | gi|148377766 | MAG_5040 |
|  | XAA-Pro aminopeptidase | *M. agalactiae* PG2 | 40001 | 5.88 | 168 | 9 | 16% | gi|148377385 | MAG_1180 |
|  | Phosphopentomutase | *M. agalactiae* PG2 | 43893 | 5.48 | 111 | 4 | 7% | gi|148377546 | MAG_2800 |
|  | Alkylphosphonate ABC transporter substrate-binding protein | *M. agalactiae* PG2 | 49744 | 6.99 | 106 | 2 | 6% | gi|148377535 | MAG_2690 |
|  | P48. lipoprotein | *M. agalactiae* PG2 | 51232 | 8.39 | 95 | 2 | 4% | gi|148377280 | MAG_0120 |
|  | Tyrosyl-tRNA synthetase 1 | *M. agalactiae* PG2 | 46669 | 7.59 | 54 | 2 | 5% | gi|148377992 | MAG_7300 |
|  | Pyruvate dehydrogenase E1 component. alphasubunit | *M. agalactiae* PG2 | 41321 | 6.13 | 43 | 1 | 3% | gi|148377361 | MAG_0930 |
|  | Hypothetical protein MAG_2810 | *M. agalactiae* PG2 | 48061 | 9.19 | 40 | 2 | 3% | gi|148377547 | MAG_2810 |
|  | DNA-directed RNA polymerase subunit alpha | *M. agalactiae* PG2 | 37453 | 5.98 | 36 | 2 | 5% | gi|148377782 | MAG_5200 |
|  |  |  |  |  |  |  |  |  |  |
| 25 | Elongation factor Tu | *M. agalactiae* PG2 | 43638 | 5.89 | 1296 | 27 | 39% | gi|148377586 | MAG_3200 |
|  | Pyruvate dehydrogenase E1 component. alphasubunit | *M. agalactiae* PG2 | 41321 | 6.13 | 581 | 21 | 31% | gi|148377361 | MAG_0930 |
|  | Variable surface lipoprotein Y | *M. agalactiae* PG2 | 37528 | 8.81 | 502 | 16 | 33% | gi|148377970 | MAG_7080 |
|  | Hypothetical protein MAG_5040 | *M. agalactiae* PG2 | 44828 | 8.46 | 475 | 16 | 33% | gi|148377766 | MAG_5040 |
|  | DNA-directed RNA polymerase subunit alpha | *M. agalactiae* PG2 | 37453 | 5.98 | 156 | 5 | 16% | gi|148377782 | MAG_5200 |
|  | XAA-Pro aminopeptidase | *M. agalactiae* PG2 | 40001 | 5.88 | 111 | 4 | 11% | gi|148377385 | MAG_1180 |
|  | Alcohol dehydrogenase | *M. agalactiae* PG2 | 37640 | 6.31 | 111 | 3 | 10% | gi|148377540 | MAG_2740 |
|  | Hypothetical protein MAG_6740 | *M. agalactiae* PG2 | 40257 | 5.55 | 96 | 2 | 3% | gi|148377936 | MAG_6740 |
|  | Alcohol dehydrogenase | *M. agalactiae* PG2 | 38157 | 6.28 | 92 | 3 | 8% | gi|148377697 | MAG_4340 |
|  | Phosphoglycerate kinase | *M. agalactiae* PG2 | 42781 | 5.79 | 86 | 5 | 12% | gi|148377849 | MAG_5860 |
|  | Alkylphosphonate ABC transporter substrate-binding protein | *M. agalactiae* PG2 | 49744 | 6.99 | 71 | 1 | 3% | gi|148377535 | MAG_2690 |
|  | Phosphopentomutase | *M. agalactiae* PG2 | 43893 | 5.48 | 59 | 2 | 5% | gi|148377546 | MAG_2800 |
|  | Acetate kinase | *M. agalactiae* PG2 | 44283 | 6.24 | 55 | 1 | 3% | gi|148377407 | MAG_1400 |
|  | ABC transporter ATP-binding protein | *M. agalactiae* PG2 | 40400 | 9.22 | 43 | 1 | 3% | gi|148378005 | MAG_7430 |
|  | Hypothetical protein MAG_2810 | *M. agalactiae* PG2 | 48061 | 9.19 | 39 | 1 | 1% | gi|148377547 | MAG_2810 |
|  | Variable surface lipoprotein U | *M. agalactiae* PG2 | 25529 | 8.62 | 33 | 1 | 3% | gi|148377971 | MAG_7090 |
|  |  |  |  |  |  |  |  |  |  |
| 26 | Variable surface lipoprotein Y | *M. agalactiae* PG2 | 37528 | 8.81 | 1004 | 28 | 32% | gi|148377970 | MAG_7080 |
|  | Pyruvate dehydrogenase E1 component. alphasubunit | *M. agalactiae* PG2 | 41321 | 6.13 | 500 | 14 | 37% | gi|148377361 | MAG_0930 |
|  | D-lactate dehydrogenase | *M. agalactiae* PG2 | 37021 | 6.11 | 424 | 11 | 31% | gi|148377416 | MAG_1490 |
|  | P40. lipoprotein | *M. agalactiae* PG2 | 39951 | 8.19 | 380 | 9 | 25% | gi|148377507 | MAG_2410 |
|  | Elongation factor Tu | *M. agalactiae* PG2 | 43638 | 5.89 | 366 | 10 | 28% | gi|148377586 | MAG_3200 |
|  | 30S ribosomal protein S7 | *M. agalactiae* PG2 | 35450 | 9.51 | 303 | 9 | 39% | gi|148377525 | MAG_2590 |
|  | Alcohol dehydrogenase | *M. agalactiae* PG2 | 37640 | 6.31 | 115 | 3 | 15% | gi|148377540 | MAG_2740 |
|  | Hypothetical protein MAG_5040 | *M. agalactiae* PG2 | 44828 | 8.46 | 91 | 3 | 9% | gi|148377766 | MAG_5040 |
|  | Glyceraldehyde 3-phosphate dehydrogenase (GAPDH) | *M. agalactiae* PG2 | 36871 | 6.72 | 67 | 1 | 3% | gi|148377323 | MAG_0550 |
|  | Putative glycerol-3-phosphate acyltransferase PlsX | *M. agalactiae* PG2 | 37025 | 8.14 | 29 | 2 | 5% | gi|148377703 | MAG_4400 |
|  |  |  |  |  |  |  |  |  |  |
| 27 | D-lactate dehydrogenase | *M. agalactiae* PG2 | 37021 | 6.11 | 649 | 15 | 47% | gi|148377416 | MAG_1490 |
|  | Pyruvate dehydrogenase E1 component. betasubunit | *M. agalactiae* PG2 | 36129 | 5.44 | 634 | 16 | 40% | gi|148377362 | MAG_0940 |
|  | Variable surface lipoprotein Y | *M. agalactiae* PG2 | 37528 | 8.81 | 517 | 10 | 24% | gi|148377970 | MAG_7080 |
|  | Glyceraldehyde 3-phosphate dehydrogenase (GAPDH) | *M. agalactiae* PG2 | 36871 | 6.72 | 428 | 8 | 27% | gi|148377323 | MAG_0550 |
|  | Hypothetical protein MAG_4450 | *M. agalactiae* PG2 | 37490 | 5.32 | 258 | 4 | 11% | gi|148377708 | MAG_4450 |
|  | P40. lipoprotein | *M. agalactiae* PG2 | 39951 | 8.19 | 243 | 6 | 17% | gi|148377507 | MAG_2410 |
|  | Variable surface lipoprotein W | *M. agalactiae* PG2 | 35472 | 9.54 | 226 | 4 | 7% | gi|148377968 | MAG_7060 |
|  | Phosphotransacetylase | *M. agalactiae* PG2 | 34447 | 6.11 | 208 | 4 | 12% | gi|148377406 | MAG_1390 |
|  | 30S ribosomal protein S7 | *M. agalactiae* PG2 | 35450 | 9.51 | 155 | 6 | 18% | gi|148377525 | MAG_2590 |
|  | Lipoprotein. MAG_2350 | *M. agalactiae* PG2 | 40383 | 8.61 | 131 | 4 | 13% | gi|148377501 | MAG_2350 |
|  | Hypothetical protein MAG_1780 | *M. agalactiae* PG2 | 37986 | 6.06 | 113 | 4 | 15% | gi|148377444 | MAG_1780 |
|  | Pyruvate dehydrogenase E1 component. alphasubunit | *M. agalactiae* PG2 | 41321 | 6.13 | 98 | 2 | 7% | gi|148377361 | MAG_0930 |
|  | Elongation factor Tu | *M. agalactiae* PG2 | 43638 | 5.89 | 77 | 1 | 3% | gi|148377586 | MAG_3200 |
|  | L-lactate dehydrogenase (L-LDH) | *M. agalactiae* PG2 | 34965 | 6.13 | 69 | 1 | 3% | gi|148377752 | MAG_4900 |
|  | Lipoate-protein ligase A | *M. agalactiae* PG2 | 37286 | 6.42 | 60 | 2 | 5% | gi|148377329 | MAG_0600 |
|  | Hypothetical protein MAG_4440 | *M. agalactiae* PG2 | 36488 | 5.87 | 33 | 1 | 2% | gi|148377707 | MAG_4440 |
|  |  |  |  |  |  |  |  |  |  |
| 28 | Pyruvate dehydrogenase E1 component. betasubunit | *M. agalactiae* PG2 | 36129 | 5.44 | 950 | 29 | 25% | gi|148377362 | MAG_0940 |
|  | L-lactate dehydrogenase (L-LDH) | *M. agalactiae* PG2 | 34965 | 6.13 | 567 | 21 | 35% | gi|148377752 | MAG_4900 |
|  | Elongation factor Ts (EF-Ts) | *M. agalactiae* PG2 | 32763 | 5.18 | 539 | 8 | 24% | gi|148377526 | MAG_2600 |
|  | Elongation factor Tu | *M. agalactiae* PG2 | 43638 | 5.89 | 232 | 5 | 14% | gi|148377586 | MAG_3200 |
|  | Hypothetical protein MAG_7400 | *M. agalactiae* PG2 | 37245 | 9.18 | 170 | 5 | 14% | gi|148378002 | MAG_7400 |
|  | Phosphotransacetylase | *M. agalactiae* PG2 | 34447 | 6.11 | 152 | 4 | 10% | gi|148377406 | MAG_1390 |
|  | Lipoprotein. MAG_1050 | *M. agalactiae* PG2 | 37025 | 9.26 | 129 | 5 | 15% | gi|148377373 | MAG_1050 |
|  | Glyceraldehyde 3-phosphate dehydrogenase (GAPDH) | *M. agalactiae* PG2 | 36871 | 6.72 | 128 | 3 | 11% | gi|148377323 | MAG_0550 |
|  | 50S ribosomal protein L4 | *M. agalactiae* PG2 | 32406 | 10.26 | 126 | 6 | 18% | gi|148377808 | MAG_5450 |
|  | D-lactate dehydrogenase | *M. agalactiae* PG2 | 37021 | 6.11 | 92 | 2 | 6% | gi|148377416 | MAG_1490 |
|  | Glycerol-3-phosphate dehydrogenase | *M. agalactiae* PG2 | 36931 | 7.01 | 85 | 5 | 11% | gi|148377318 | MAG_0500 |
|  | Hypothetical protein MAG_4440 | *M. agalactiae* PG2 | 36488 | 5.87 | 85 | 4 | 7% | gi|148377707 | MAG_4440 |
|  | Hypothetical protein MAG_4450 | *M. agalactiae* PG2 | 37490 | 5.32 | 85 | 2 | 6% | gi|148377708 | MAG_4450 |
|  | 50S ribosomal protein L2 | *M. agalactiae* PG2 | 30773 | 10.63 | 72 | 4 | 9% | gi|148377806 | MAG_5430 |
|  | Hypothetical protein MAG_1450 | *M. agalactiae* PG2 | 35463 | 9.15 | 71 | 4 | 11% | gi|148377412 | MAG_1450 |
|  | 5'nucleotidase | *M. agalactiae* PG2 | 76195 | 8.36 | 60 | 1 | 1% | gi|148377854 | MAG_5910 |
|  | Pyruvate dehydrogenase E1 component. alphasubunit | *M. agalactiae* PG2 | 41321 | 6.13 | 59 | 2 | 5% | gi|148377361 | MAG_0930 |
|  | Variable surface lipoprotein A | *M. agalactiae* PG2 | 24769 | 8.33 | 56 | 3 | 14% | gi|148377969 | MAG_7070 |
|  | Lipoprotein. MAG_2430 | *M. agalactiae* PG2 | 33862 | 8.29 | 52 | 2 | 7% | gi|148377509 | MAG_2430 |
|  | 30S ribosomal protein S7 | *M. agalactiae* PG2 | 35450 | 9.51 | 46 | 2 | 2% | gi|148377525 | MAG_2590 |
|  | Malate permease | *M. agalactiae* PG2 | 45146 | 9.75 | 42 | 1 | 2% | gi|148377751 | MAG_4890 |
|  | P30. lipoprotein | *M. agalactiae* PG2 | 29213 | 9.29 | 42 | 1 | 4% | gi|148377613 | MAG_3470 |
|  | Lipoate-protein ligase A | *M. agalactiae* PG2 | 37286 | 6.42 | 27 | 2 | 5% | gi|148377329 | MAG_0600 |
|  |  |  |  |  |  |  |  |  |  |
| 29 | Lipoprotein. MAG_2430 | *M. agalactiae* PG2 | 33862 | 8.29 | 1289 | 31 | 54% | gi|148377509 | MAG_2430 |
|  | Elongation factor Ts (EF-Ts) | *M. agalactiae* PG2 | 32763 | 5.18 | 353 | 8 | 32% | gi|148377526 | MAG_2600 |
|  | Variable surface lipoprotein D (Variable surface lipopr. Z) | *M. agalactiae* PG2 | 36608 | 8.52 | 240 | 8 | 15% | gi|148377972 | MAG_7100 |
|  | Lipoprotein. MAG_1050 | *M. agalactiae* PG2 | 37025 | 9.26 | 224 | 4 | 16% | gi|148377373 | MAG_1050 |
|  | Pyruvate dehydrogenase E1 component. betasubunit | *M. agalactiae* PG2 | 36129 | 5.44 | 220 | 7 | 20% | gi|148377362 | MAG_0940 |
|  | L-lactate dehydrogenase (L-LDH) | *M. agalactiae* PG2 | 34965 | 6.13 | 219 | 7 | 23% | gi|148377752 | MAG_4900 |
|  | Malate permease | *M. agalactiae* PG2 | 45146 | 9.75 | 216 | 3 | 6% | gi|148377751 | MAG_4890 |
|  | 50S ribosomal protein L3 | *M. agalactiae* PG2 | 28793 | 9.75 | 194 | 10 | 34% | gi|148377809 | MAG_5460 |
|  | 50S ribosomal protein L2 | *M. agalactiae* PG2 | 30773 | 10.63 | 172 | 8 | 23% | gi|148377806 | MAG_5430 |
|  | phosphate acetyltransferase (Phosphotransacetylase) | *M. agalactiae* PG2 | 35626 | 6.75 | 110 | 2 | 7% | gi|148377754 | MAG_4920 |
|  | Elongation factor Tu | *M. agalactiae* PG2 | 43638 | 5.89 | 97 | 2 | 6% | gi|148377586 | MAG_3200 |
|  | Hypothetical protein MAG_0250 | *M. agalactiae* PG2 | 32658 | 6.55 | 67 | 1 | 3% | gi|148377293 | MAG_0250 |
|  | Hypothetical protein MAG_1450 | *M. agalactiae* PG2 | 35463 | 9.15 | 58 | 1 | 3% | gi|148377412 | MAG_1450 |
|  | Pyruvate dehydrogenase E1 component. alphasubunit | *M. agalactiae* PG2 | 41321 | 6.13 | 37 | 1 | 3% | gi|148377361 | MAG_0930 |
|  | 50S ribosomal protein L1 | *M. agalactiae* PG2 | 24955 | 9.68 | 34 | 2 | 9% | gi|148377349 | MAG_0810 |
|  | 50S ribosomal protein L4 | *M. agalactiae* PG2 | 32406 | 10.26 | 30 | 2 | 8% | gi|148377808 | MAG_5450 |
|  |  |  |  |  |  |  |  |  |  |
| 30 | Lipoprotein. MAG_2430 | *M. agalactiae* PG2 | 33862 | 8.29 | 1053 | 35 | 42% | gi|148377509 | MAG_2430 |
|  | 50S ribosomal protein L1 | *M. agalactiae* PG2 | 24955 | 9.68 | 354 | 15 | 30% | gi|148377349 | MAG_0810 |
|  | 50S ribosomal protein L3 | *M. agalactiae* PG2 | 28793 | 9.75 | 256 | 10 | 20% | gi|148377809 | MAG_5460 |
|  | Variable surface lipoprotein D | *M. agalactiae* PG2 | 36608 | 8.52 | 227 | 16 | 19% | gi|148377972 | MAG_7100 |
|  | 50S ribosomal protein L2 | *M. agalactiae* PG2 | 30773 | 10.63 | 174 | 12 | 12% | gi|148377806 | MAG_5430 |
|  | L-lactate dehydrogenase (L-LDH) | *M. agalactiae* PG2 | 34965 | 6.13 | 149 | 3 | 6% | gi|148377752 | MAG_4900 |
|  | Elongation factor Ts (EF-Ts) | *M. agalactiae* PG2 | 32763 | 5.18 | 62 | 1 | 5% | gi|148377526 | MAG_2600 |
|  | Cobalt transporter ATP-binding subunit | *M. agalactiae* PG2 | 29354 | 6.03 | 51 | 1 | 3% | gi|148377780 | MAG_5180 |
|  | Pyruvate dehydrogenase E1 component. betasubunit | *M. agalactiae* PG2 | 36129 | 5.44 | 51 | 1 | 3% | gi|148377362 | MAG_0940 |
|  | Lipoprotein. MAG_6200 | *M. agalactiae* PG2 | 26952 | 9.22 | 42 | 2 | 9% | gi|148377883 | MAG_6200 |
|  | Methionyl-tRNA formyltransferase | *M. agalactiae* PG2 | 31227 | 9.21 | 34 | 1 | 2% | gi|148377891 | MAG_6280 |
|  | F0F1 ATP synthase subunit gamma | *M. agalactiae* PG2 | 32874 | 9.01 | 31 | 1 | 2% | gi|148377621 | MAG_3550 |
|  |  |  |  |  |  |  |  |  |  |
| 31 | Hypothetical protein MAG_1220 | *M. agalactiae* PG2 | 26491 | 8.69 | 323 | 13 | 34% | gi|148377389 | MAG_1220 |
|  | Lipoprotein. MAG_6200 | *M. agalactiae* PG2 | 26952 | 9.22 | 299 | 4 | 9% | gi|148377883 | MAG_6200 |
|  | 50S ribosomal protein L1 | *M. agalactiae* PG2 | 24955 | 9.68 | 201 | 9 | 26% | gi|148377349 | MAG_0810 |
|  | Uridylate kinase | *M. agalactiae* PG2 | 26309 | 6.64 | 154 | 3 | 16% | gi|148377314 | MAG_0460 |
|  | 30S ribosomal protein S5 | *M. agalactiae* PG2 | 25266 | 10.25 | 151 | 10 | 28% | gi|148377792 | MAG_5300 |
|  | Lipoprotein. MAG_2430 | *M. agalactiae* PG2 | 33862 | 8.29 | 120 | 5 | 13% | gi|148377509 | MAG_2430 |
|  | L-lactate dehydrogenase (L-LDH) | *M. agalactiae* PG2 | 34965 | 6.13 | 101 | 4 | 9% | gi|148377752 | MAG_4900 |
|  | 30S ribosomal protein S68 | *M. agalactiae* PG2 | 12097 | 10.2 | 86 | 1 | 15% | gi|148377544 | MAG_2780 |
|  | ABC transporter permease protein | *M. agalactiae* PG2 | 39653 | 9.72 | 73 | 1 | 3% | gi|148377768 | MAG_5060 |
|  | Triosephosphate isomerase | *M. agalactiae* PG2 | 29382 | 6.18 | 62 | 1 | 5% | gi|148377776 | MAG_5140 |
|  | HAD superfamily hydrolase | *M. agalactiae* PG2 | 31183 | 9.45 | 57 | 1 | 3% | gi|148377295 | MAG_0270 |
|  | Variable surface lipoprotein D | *M. agalactiae* PG2 | 36608 | 8.52 | 56 | 3 | 9% | gi|148377972 | MAG_7100 |
|  | Cobalt transporter ATP-binding subunit | *M. agalactiae* PG2 | 29354 | 6.03 | 52 | 1 | 3% | gi|148377780 | MAG_5180 |
|  | 50S ribosomal protein L2 | *M. agalactiae* PG2 | 30773 | 10.63 | 48 | 3 | 9% | gi|148377806 | MAG_5430 |
|  | Pyruvate dehydrogenase E1 component. betasubunit | *M. agalactiae* PG2 | 36129 | 5.44 | 48 | 1 | 3% | gi|148377362 | MAG_0940 |
|  | Translation initiation factor IF-3 | *M. agalactiae* PG2 | 23332 | 9.19 | 39 | 2 | 7% | gi|148377738 | MAG_4750 |
|  | Methionyl-tRNA formyltransferase | *M. agalactiae* PG2 | 31227 | 9.21 | 35 | 1 | 2% | gi|148377891 | MAG_6280 |
|  | Alanyl-tRNA synthetase | *M. agalactiae* PG2 | 100239 | 5.37 | 32 | 1 | <1% | gi|148377682 | MAG_4160 |
|  | 50S ribosomal protein L3 | *M. agalactiae* PG2 | 28793 | 9.75 | 26 | 2 | 7% | gi|148377809 | MAG_5460 |
|  |  |  |  |  |  |  |  |  |  |
| 32 | 30S ribosomal protein S5 | *M. agalactiae* PG2 | 25266 | 10.25 | 384 | 17 | 38% | gi|148377792 | MAG_5300 |
|  | Hypothetical protein MAG_1220 | *M. agalactiae* PG2 | 26491 | 8.69 | 320 | 13 | 30% | gi|148377389 | MAG_1220 |
|  | Lipoprotein. MAG_6200 | *M. agalactiae* PG2 | 26952 | 9.22 | 178 | 3 | 9% | gi|148377883 | MAG_6200 |
|  | Uridylate kinase | *M. agalactiae* PG2 | 26309 | 6.64 | 136 | 4 | 16% | gi|148377314 | MAG_0460 |
|  | Prolipoprotein diacylglyceryl transferase | *M. agalactiae* PG2 | 37843 | 9.3 | 90 | 1 | 3% | gi|148377358 | MAG_0900 |
|  | 50S ribosomal protein L1 | *M. agalactiae* PG2 | 24955 | 9.68 | 90 | 3 | 18% | gi|148377349 | MAG_0810 |
|  | 30S ribosomal protein S68 | *M. agalactiae* PG2 | 12097 | 10.2 | 85 | 1 | 15% | gi|148377544 | MAG_2780 |
|  | 30S ribosomal protein S8 | *M. agalactiae* PG2 | 24090 | 9.91 | 67 | 2 | 8% | gi|148377803 | MAG_5400 |
|  | Lipoprotein. MAG_2000 | *M. agalactiae* PG2 | 26602 | 8.39 | 51 | 1 | 6% | gi|148377466 | MAG_2000 |
|  | Triosephosphate isomerase | *M. agalactiae* PG2 | 29382 | 6.18 | 50 | 1 | 15% | gi|148377776 | MAG_5140 |
|  | ABC transporter permease protein | *M. agalactiae* PG2 | 39653 | 9.72 | 46 | 1 | 3% | gi|148377768 | MAG_5060 |
|  | Dihydrolipoamide acetyltransferase component of pyruvate deshydrogenase complex | *M. agalactiae* PG2 | 26767 | 7.68 | 33 | 1 | 5% | gi|148377363 | MAG_0950 |
|  | ABC transporter. ATP binding protein | *M. agalactiae* PG2 | 27496 | 8.58 | 31 | 1 | 3% | gi|148377430 | MAG_1630 |
|  | Pyruvate dehydrogenase E1 component. alphasubunit | *M. agalactiae* PG2 | 41321 | 6.13 | 28 | 1 | 2% | gi|148377361 | MAG_0930 |
|  | Pyruvate dehydrogenase E1 component. betasubunit | *M. agalactiae* PG2 | 36129 | 5.44 | 25 | 1 | 3% | gi|148377362 | MAG_0940 |
|  |  |  |  |  |  |  |  |  |  |
| 33 | 30S ribosomal protein S8 | *M. agalactiae* PG2 | 24090 | 9.91 | 452 | 13 | 29% | gi|148377803 | MAG_5400 |
|  | 30S ribosomal protein S5 | *M. agalactiae* PG2 | 25266 | 10.25 | 147 | 6 | 25% | gi|148377792 | MAG_5300 |
|  | P40. lipoprotein | *M. agalactiae* PG2 | 39951 | 8.19 | 132 | 3 | 9% | gi|148377507 | MAG_2410 |
|  | Hypothetical protein MAG_1220 | *M. agalactiae* PG2 | 26491 | 8.69 | 119 | 4 | 18% | gi|148377389 | MAG_1220 |
|  | 3-keto-L-gulonate-6-phosphate decarboxylase | *M. agalactiae* PG2 | 24126 | 6.64 | 88 | 3 | 5% | gi|148377898 | MAG_6350 |
|  | Pyruvate dehydrogenase E1 component. betasubunit | *M. agalactiae* PG2 | 36129 | 5.44 | 64 | 1 | 3% | gi|148377362 | MAG_0940 |
|  | Lipoprotein. MAG_2000 | *M. agalactiae* PG2 | 26602 | 8.29 | 35 | 2 | 6% | gi|148377466 | MAG_2000 |
|  |  |  |  |  |  |  |  |  |  |
| 34 | Lipoprotein. MAG_3600 | *M. agalactiae* PG2 | 21678 | 9.13 | 579 | 14 | 35% | gi|148377626 | MAG_3600 |
|  | 30S ribosomal protein S8 | *M. agalactiae* PG2 | 24090 | 9.91 | 228 | 9 | 23% | gi|148377803 | MAG_5400 |
|  | 1-acyl-SN-Glycerol-3-phosphate acyltransferase | *M. agalactiae* PG2 | 28539 | 9.95 | 184 | 8 | 33% | gi|148377984 | MAG_7220 |
|  | P40. lipoprotein | *M. agalactiae* PG2 | 39951 | 8.19 | 162 | 2 | 3% | gi|148377507 | MAG_2410 |
|  | 30S ribosomal protein S4 | *M. agalactiae* PG2 | 22616 | 10.21 | 121 | 14 | 27% | gi|148377824 | MAG_5610 |
|  | Transcription antitermination protein NusG | *M. agalactiae* PG2 | 22639 | 6.22 | 61 | 1 | 7% | gi|148377312 | MAG_0440 |
|  | Variable surface lipoprotein Y | *M. agalactiae* PG2 | 37528 | 8.81 | 52 | 1 | 7% | gi|148377970 | MAG_7080 |
|  | Hypothetical protein MAG_1220 | *M. agalactiae* PG2 | 26491 | 8.69 | 46 | 1 | 3% | gi|148377389 | MAG_1220 |
|  | Pyruvate dehydrogenase E1 component. betasubunit | *M. agalactiae* PG2 | 36129 | 5.44 | 39 | 1 | 3% | gi|148377362 | MAG_0940 |
|  | Thymidine kinase | *M. agalactiae* PG2 | 21731 | 8.45 | 29 | 1 | 4% | gi|148377963 | MAG_7010 |
|  | Acyl carrier protein phosphodiesterase | *M. agalactiae* PG2 | 22409 | 9.32 | 26 | 3 | 5% | gi|148377731 | MAG_3680 |
|  |  |  |  |  |  |  |  |  |  |
| 35 | Lipoprotein. MAG_3600 | *M. agalactiae* PG2 | 21678 | 9.13 | 221 | 9 | 42% | gi|148377626 | MAG_3600 |
|  | Variable surface lipoprotein Y | *M. agalactiae* PG2 | 37528 | 8.81 | 177 | 5 | 14% | gi|148377970 | MAG_7080 |
|  | L-lactate dehydrogenase (L-LDH) | *M. agalactiae* PG2 | 34965 | 6.13 | 101 | 3 | 9% | gi|148377752 | MAG_4900 |
|  | 50S ribosomal protein L6 | *M. agalactiae* PG2 | 19288 | 9.88 | 96 | 2 | 5% | gi|148377794 | MAG_5320 |
|  | Acyl carrier protein phosphodiesterase | *M. agalactiae* PG2 | 22409 | 9.32 | 62 | 2 | 5% | gi|148377731 | MAG_3680 |
|  | 30S ribosomal protein S4 | *M. agalactiae* PG2 | 22616 | 10.21 | 43 | 3 | 13% | gi|148377824 | MAG_5610 |
|  | Variable surface lipoprotein A | *M. agalactiae* PG2 | 24769 | 8.33 | 37 | 2 | 15% | gi|148377969 | MAG_7070 |
|  | Lipoprotein. MAG_2400 | *M. agalactiae* PG2 | 38065 | 8.95 | 36 | 2 | 5% | gi|148377506 | MAG_2400 |
|  |  |  |  |  |  |  |  |  |  |
| 36 | Variable surface lipoprotein Y | *M. agalactiae* PG2 | 37528 | 8.81 | 608 | 14 | 23% | gi|148377970 | MAG_7080 |
|  | Variable surface lipoprotein A | *M. agalactiae* PG2 | 24769 | 8.33 | 353 | 6 | 25% | gi|148377969 | MAG_7070 |
|  | 30S ribosomal protein S7 | *M. agalactiae* PG2 | 17810 | 10.24 | 327 | 12 | 39% | gi|148377856 | MAG_5930 |
|  | 30S ribosomal protein S68 | *M. agalactiae* PG2 | 12097 | 10.2 | 168 | 3 | 24% | gi|148377544 | MAG_2780 |
|  | 50S ribosomal protein L13 | *M. agalactiae* PG2 | 16160 | 10.07 | 161 | 4 | 18% | gi|148377718 | MAG_4550 |
|  | 50S ribosomal protein L5 | *M. agalactiae* PG2 | 20778 | 9.85 | 125 | 8 | 23% | gi|148377797 | MAG_5350 |
|  | Hypothetical protein MAG_6920 | *M. agalactiae* PG2 | 20745 | 5.99 | 116 | 2 | 13% | gi|148377954 | MAG_6920 |
|  | 50S ribosomal protein L18 | *M. agalactiae* PG2 | 12978 | 10.25 | 94 | 3 | 14% | gi|148377793 | MAG_5310 |
|  | 50S ribosomal protein L10 | *M. agalactiae* PG2 | 18901 | 9.26 | 91 | 3 | 17% | gi|148377882 | MAG_6190 |
|  | 30S ribosomal protein S9 | *M. agalactiae* PG2 | 14863 | 11.42 | 91 | 4 | 20% | gi|148377719 | MAG_4560 |
|  | 30S ribosomal protein S60 | *M. agalactiae* PG2 | 12101 | 10.24 | 86 | 1 | 13% | gi|148377810 | MAG_5470 |
|  | 30S ribosomal protein S69 | *M. agalactiae* PG2 | 10395 | 9.94 | 82 | 3 | 8% | gi|148377805 | MAG_5420 |
|  | 50S ribosomal protein L17 | *M. agalactiae* PG2 | 13876 | 10.53 | 81 | 5 | 25% | gi|148377781 | MAG_5190 |
|  | 50S ribosomal protein L32 | *M. agalactiae* PG2 | 7922 | 10.31 | 79 | 1 | 21% | gi|148377396 | MAG_1290 |
|  | 50S ribosomal protein L11 | *M. agalactiae* PG2 | 16890 | 9.37 | 70 | 1 | 8% | gi|148377348 | MAG_0800 |
|  | 50S ribosomal protein L23 | *M. agalactiae* PG2 | 16678 | 9.63 | 67 | 2 | 12% | gi|148377807 | MAG_5440 |
|  | 30S ribosomal protein S63 | *M. agalactiae* PG2 | 14057 | 10.49 | 66 | 4 | 28% | gi|148377784 | MAG_5220 |
|  | Lipoprotein. MAG_2400 | *M. agalactiae* PG2 | 38065 | 8.95 | 65 | 2 | 5% | gi|148377506 | MAG_2400 |
|  | 30S ribosomal protein S62 | *M. agalactiae* PG2 | 15141 | 11.09 | 61 | 3 | 8% | gi|148377857 | MAG_5940 |
|  | 50S ribosomal protein L14 | *M. agalactiae* PG2 | 13262 | 9.88 | 52 | 1 | 8% | gi|148377799 | MAG_5370 |
|  | 50S ribosomal protein L27 | *M. agalactiae* PG2 | 10149 | 10.91 | 44 | 1 | 9% | gi|148377817 | MAG_5540 |
|  | 50S ribosomal protein L22 | *M. agalactiae* PG2 | 12716 | 10.88 | 40 | 1 | 6% | gi|148377804 | MAG_5410 |
|  | 30S ribosomal protein S6 | *M. agalactiae* PG2 | 16627 | 9.87 | 39 | 1 | 5% | gi|148377542 | MAG_2760 |
|  | 50S ribosomal protein L6 | *M. agalactiae* PG2 | 19288 | 9.88 | 39 | 1 | 5% | gi|148377794 | MAG_5320 |
|  | 50S ribosomal protein L15 | *M. agalactiae* PG2 | 15751 | 10.81 | 39 | 2 | 11% | gi|148377791 | MAG_5290 |
|  | 30S ribosomal protein S61 | *M. agalactiae* PG2 | 14244 | 10.35 | 34 | 1 | 7% | gi|148377783 | MAG_5210 |
|  | 50S ribosomal protein L24 | *M. agalactiae* PG2 | 12043 | 10.3 | 33 | 1 | 7% | gi|148377798 | MAG_5360 |
|  | 50S ribosomal protein L19 | *M. agalactiae* PG2 | 13446 | 11.13 | 33 | 1 | 6% | gi|148377890 | MAG_6270 |
|  | Hypothetical protein MAG_3830 | *M. agalactiae* PG2 | 17288 | 9.3 | 32 | 1 | 7% | gi|148377649 | MAG_3830 |
