# Supplementary material for: The liposoluble proteome of Mycoplasma agalactiae: an insight into the minimal protein complement of a bacterial membrane
Source: BMC Microbiol. 2010 Aug 25;10:225. doi: 10.1186/1471-2180-10-225 (PMC2941501; doi:10.1186/1471-2180-10-225)
Supplement: Additional file 7 — Functional analysis, number of peptide hits, and method of detection of M. agalactiae PG2T liposoluble proteins. The results of 2D DIGE with the two field strains Nurri and Bortigali are also reported (TPH, total peptide hits; NA, not applicable). [file 1471-2180-10-225-S7.DOC]

### Additional file 7. Functional analysis, number of peptide hits, and method of detection of *M. agalactiae* PG2^T^ liposoluble proteins. The results of 2D DIGE with the two field strains Nurri and Bortigali are also reported (TPH, total peptide hits; NA, not applicable).

| **Name** | **Locus** | **Function** | **TPH** | **2D-PAGE-MS** | **GeLC-MS/MS** | **PG2^T^** | **Nurri** | **Bortigali** |
| --- | --- | --- | --- | --- | --- | --- | --- | --- |
| P48, lipoprotein, MAG_0120 | MAG_0120 | ABC transporter | 94 | X | X | X | X | X |
| ABC transporter, ATP-binding protein P59, MAG_0140 | MAG_0140 | ABC transporter | 40 |  | X | NA | NA | NA |
| Sugar ABC transporter permease, MAG_0150 | MAG_0150 | ABC transporter | 26 |  | X | NA | NA | NA |
| Hypothetical protein MAG_0250 | MAG_0250 | Indigoidine sinthase A superfamily, putative virulence factor | 1 |  | X | NA | NA | NA |
| HAD superfamily hydrolase, MAG_0270 | MAG_0270 | Hydrolase | 1 |  | X | NA | NA | NA |
| Hypothetical protein MAG_0280 | MAG_0280 | ABC transporter | 10 |  | X | NA | NA | NA |
| Oligopeptide ABC transporter, substrate-bindingprotein (OppA), lipoprotein, MAG_0380 | MAG_0380 | ABC transporter | 1 |  | X | NA | NA | NA |
| Transcription antitermination protein NusG, MAG_0440 | MAG_0440 | Transcription | 1 |  | X | NA | NA | NA |
| Uridylate kinase, MAG_0460 | MAG_0460 | Nucleotide metabolism | 7 |  | X | NA | NA | NA |
| Glycerol-3-phosphate dehydrogenase, MAG_0500 | MAG_0500 | Lipid metabolism | 5 |  | X | NA | NA | NA |
| Glyceraldehyde 3-phosphate dehydrogenase (GAPDH), MAG_0550 | MAG_0550 | Carbohydrate metabolism | 12 | X | X | X | X | X |
| Seryl-tRNA synthetase, MAG_0560 | MAG_0560 | Amino acid metabolism | 2 |  | X | NA | NA | NA |
| Putative inner membrane protein translocase component YidC MAG_0590 | MAG_0590 | Secretion system/Protein export | 57 |  | X | NA | NA | NA |
| Lipoate-protein ligase A, MAG_0600 | MAG_0600 | Metabolism of cofactors/Protein modification | 6 | X | X | NA | NA | NA |
| 50S ribosomal protein L11, MAG_0800 | MAG_0800 | Translation | 1 |  | X | NA | NA | NA |
| 50S ribosomal protein L1, MAG_0810 | MAG_0810 | Translation | 31 | X | X | X | X | X |
| Prolipoprotein diacylglyceryl transferase MAG_0900 | MAG_0900 | Secretion system/Protein export | 2 |  | X | NA | NA | NA |
| Pyruvate dehydrogenase E1 component, alphasubunit, MAG_0930 | MAG_0930 | Carbohydrate metabolism | 72 | X | X | X | X | X |
| Pyruvate dehydrogenase E1 component, betasubunit, MAG_0940 | MAG_0940 | Carbohydrate metabolism | 69 | X | X | X | X | X |
| Dihydrolipoamide acetyltransferase component of pyruvate dehydrogenase complex, MAG_0950 | MAG_0950 | Carbohydrate metabolism | 1 |  | X | NA | NA | NA |
| Dihydrolipoamide dehydrogenase (E3 component of pyruvate complex), MAG_0960 | MAG_0960 | Carbohydrate metabolism/Amino acid metabolism | 27 |  | X | NA | NA | NA |
| Hypotetical protein MAG_1000 | MAG_1000 | ABC transporter | 87 | X | X | X | X | X |
| Oligopeptide ABC transporter system, permeaseprotein (OppC), MAG_1020 | MAG_1020 | ABC transporter | 7 |  | X | NA | NA | NA |
| Oligopeptide ABC transporter, ATP-bindingprotein (OppF), MAG_1040 | MAG_1040 | ABC transporter | 11 |  | X | NA | NA | NA |
| Lipoprotein, MAG_1050 | MAG_1050 |  | 9 | X | X | NA | NA | NA |
| XAA-Pro aminopeptidase, MAG_1180 | MAG_1180 | Hydrolase | 41 |  | X | NA | NA | NA |
| Prolyl-tRNA synthetase, MAG_1190 | MAG_1190 | Amino acid metabolism | 5 |  | X | NA | NA | NA |
| Hypothetical membrane protein MAG_1210 | MAG_1210 |  | 10 |  | X | NA | NA | NA |
| Hypothetical transmembrane protein MAG_1220 | MAG_1220 | LemA family | 32 | X | X | X |  | X |
| Putative phosphoketolase, MAG_1230 | MAG_1230 | Carbohydrate metabolism/Energy metabolism | 80 |  | X | NA | NA | NA |
| Spermidine/putrescine ABC transporter ATP-binding protein, MAG_1250 | MAG_1250 | ABC transporter | 2 |  | X | NA | NA | NA |
| 50S ribosomal protein L32, MAG_1290 | MAG_1290 | Translation | 1 |  | X | NA | NA | NA |
| Valyl-tRNA synthetase, MAG_1370 | MAG_1370 | Amino acid metabolism | 2 |  | X | NA | NA | NA |
| Phosphotransacetylase, MAG_1390 | MAG_1390 | Carbohydrate metabolism/Amino acid metabolism | 8 |  | X | NA | NA | NA |
| Acetate kinase, MAG_1400 | MAG_1400 | Carbohydrate metabolism/Amino acid metabolism | 1 |  | X | NA | NA | NA |
| Hypothetical protein MAG_1430 | MAG_1430 |  | 11 |  | X | NA | NA | NA |
| Pyruvate kinase, MAG_1440 | MAG_1440 | Carbohydrate metabolism | 37 |  | X | NA | NA | NA |
| Hypothetical lipoprotein MAG_1450 | MAG_1450 |  | 5 | X | X | NA | NA | NA |
| Molecular chaperone DnaK, MAG_1460 | MAG_1460 | Nucleic acid metabolism/Membrane ion channel | 11 |  | X | NA | NA | NA |
| D-lactate dehydrogenase MAG_1490 | MAG_1490 | Carbohydrate metabolism | 28 | X | X | X | X | X |
| Type III restriction-modification system: methylase, MAG_1530 | MAG_1530 | Restriction-modification system | 9 |  | X | NA | NA | NA |
| Trigger factor, MAG_1540 | MAG_1540 | Protein folding | 6 |  | X | NA | NA | NA |
| ABC transporter, ATP binding protein, MAG_1630 | MAG_1630 | ABC transporter | 1 |  | X | NA | NA | NA |
| Hypothetical lipoprotein MAG_1670 | MAG_1670 | Mycoides cluster P72 family, putative virulence factor | 2 |  | X | NA | NA | NA |
| Hypothetical protein MAG_1780 | MAG_1780 |  | 4 |  | X | NA | NA | NA |
| Hypothetical transmembrane protein MAG_1810 | MAG_1810 |  | 2 |  | X | NA | NA | NA |
| Topoisomerase IV subunit B, MAG_1820 | MAG_1820 | Nucleic acid metabolism | 2 |  | X | NA | NA | NA |
| Hypothetical transmembrane protein MAG_1970 | MAG_1970 | M. synoviae P80 antigen-like | 19 |  | X | NA | NA | NA |
| Lipoprotein, MAG_1980 | MAG_1980 | M. synoviae P60 antigen-like | 10 | X | X | X | X | X |
| Lipoprotein, MAG_2000 | MAG_2000 |  | 3 | X | X | X | X | X |
| Methionyl-tRNA synthetase, MAG_2060 | MAG_2060 | Amino acid metabolism | 3 |  | X | NA | NA | NA |
| Ribonuclease R, MAG_2080 | MAG_2080 | Nucleic acid metabolism | 1 |  | X | NA | NA | NA |
| CTP synthetase, MAG_2190 | MAG_2190 | Nucleotide metabolism | 4 |  | X |  |  |  |
| Isoleucyl-tRNA synthetase , MAG_2200 | MAG_2200 | Amino acid metabolism | 7 |  | X |  |  |  |
| Hypothetical lipoprotein MAG_2220 | MAG_2220 |  | 82 | X | X | X | X | X |
| Protein-export membrane protein, MAG_2250 | MAG_2250 | Secretion system/Protein export | 13 |  | X | NA | NA | NA |
| Hypothetical lipoprotein, MAG_2340 | MAG_2340 |  | 5 |  | X | NA | NA | NA |
| Lipoprotein, MAG_2350 | MAG_2350 |  | 4 | X | X | X | X | X |
| Hypothetical transmembrane protein MAG_2360 | MAG_2360 | Hydrolase, DHH superfamily | 1 |  | X | NA | NA | NA |
| Replicative DNA helicase, MAG_2380 | MAG_2380 | Nucleic acid metabolism | 1 |  | X | NA | NA | NA |
| Lipoprotein, MAG_2400 | MAG_2400 |  | 4 | X | X | X | X | X |
| P40, lipoprotein, MAG_2410 | MAG_2410 |  | 20 | X | X | X | X | X |
| Lipoprotein, MAG_2430 | MAG_2430 |  | 73 | X | X | X |  | X |
| DNA recombination protein, MAG_2480 | MAG_2480 | Nucleic acid metabolism | 3 |  | X | NA | NA | NA |
| 30S ribosomal protein S7, MAG_2590 | MAG_2590 | Translation | 17 |  | X | NA | NA | NA |
| Elongation factor Ts (EF-Ts), MAG_2600 | MAG_2600 | Translation | 17 |  | X | NA | NA | NA |
| NADH oxidase (NOXASE), MAG_2630 | MAG_2630 | Energy metabolism | 18 | X | X | NA | NA | NA |
| Glycyl-tRNA synthetase, MAG_2670 | MAG_2670 | Amino acid metabolism | 1 |  | X | NA | NA | NA |
| Hypothetical transmembrane protein MAG_2680 | MAG_2680 |  | 14 |  | X | NA | NA | NA |
| Alkylphosphonate ABC transporter substrate-binding protein, MAG_2690 | MAG_2690 | ABC transporter | 9 | X | X | X | X | X |
| Preprotein translocase subunit SecA, MAG_2730 | MAG_2730 | Secretion system/Protein export | 8 |  | X | NA | NA | NA |
| Alcohol dehydrogenase, MAG_2740 | MAG_2740 | Carbohydrate metabolism/Amino acid metabolism/Lipid metabolism/Xenobiotics Biodegradation and Metabolism | 6 |  | X | NA | NA | NA |
| 30S ribosomal protein S6, MAG_2760 | MAG_2760 | Translation | 1 |  | X | NA | NA | NA |
| 30S ribosomal protein S68, MAG_2780 | MAG_2780 | Translation | 5 |  | X | NA | NA | NA |
| Phosphopentomutase, MAG_2800 | MAG_2800 | Carbohydrate metabolism/Nucleotide metabolism | 13 |  | X | NA | NA | NA |
| Hypothetical protein MAG_2810 | MAG_2810 |  | 3 |  | X | NA | NA | NA |
| DNA ligase, MAG_2820 | MAG_2820 | Nucleic acid metabolism | 3 |  | X | NA | NA | NA |
| Putative transmembrane protein, MAG_2920 | MAG_2920 |  | 1 |  | X | NA | NA | NA |
| Asparaginyl-tRNA synthetase, MAG_3020 | MAG_3020 | Amino acid metabolism | 2 |  | X | NA | NA | NA |
| Mg2+ transport protein (MGTE), MAG_3070 | MAG_3070 | Membrane transport | 3 |  | X | NA | NA | NA |
| Phosphopyruvate hydratase, MAG_3190 | MAG_3190 | Carbohydrate metabolism/RNA degradation | 2 |  | X | NA | NA | NA |
| Elongation factor Tu, MAG_3200 | MAG_3200 | Translation | 150 | X | X | NA | NA | NA |
| Hypothetical lipoprotein MAG_3240 | MAG_3240 |  | 2 |  | X | NA | NA | NA |
| Threonyl-tRNA synthetase, MAG_3440 | MAG_3440 | Amino acid metabolism | 11 |  | X | NA | NA | NA |
| P30, lipoprotein, MAG_3470 | MAG_3470 |  | 1 |  | X | NA | NA | NA |
| ATP synthase B chain, MAG_3520 | MAG_3520 | Energy metabolism | 4 | X |  | X | X | X |
| F0F1 ATP synthase subunit alpha, MAG_3540 | MAG_3540 | Energy metabolism | 3 |  | X | NA | NA | NA |
| F0F1 ATP synthase subunit gamma, MAG_3550 | MAG_3550 | Energy metabolism | 1 |  | X | NA | NA | NA |
| F0F1 ATP synthase subunit beta, MAG_3560 | MAG_3560 | Energy metabolism | 9 |  | X | NA | NA | NA |
| Lipoprotein, MAG_3600 | MAG_3600 |  | 23 | X | X | X | X | X |
| Leucyl-tRNA synthetase (leucine-tRNA ligase), MAG_3640 | MAG_3640 | Amino acid metabolism | 7 |  | X | NA | NA | NA |
| Endopeptidase O, MAG_3680 | MAG_3680 | Hydrolase | 26 |  | X | NA | NA | NA |
| Excinuclease ABC subunit B, MAG_3780 | MAG_3780 | Nucleic acid metabolism | 3 | X | X | NA | NA | NA |
| Hypothetical protein MAG_3830 | MAG_3830 | Hydrolase | 1 |  | X | NA | NA | NA |
| Alanyl-tRNA synthetase, MAG_4160 | MAG_4160 | Amino acid metabolism | 8 |  | X | NA | NA | NA |
| Alcohol dehydrogenase, MAG_4340 | MAG_4340 | Carbohydrate metabolism/Lipid metabolism/Amino acid metabolism/Xenobiotic biodegradation and metabolism | 3 |  | X | NA | NA | NA |
| Putative glycerol-3-phosphate acyltransferase PlsX, MAG_4400 | MAG_4400 | Lipid metabolism | 2 |  | X | NA | NA | NA |
| Hypothetical protein MAG_4410 | MAG_4410 |  | 1 |  | X | NA | NA | NA |
| Hypothetical protein MAG_4440 | MAG_4440 | Hydrolase, DHH family | 5 |  | X | NA | NA | NA |
| Hypothetical protein MAG_4450 | MAG_4450 | Hydrolase, DHH family | 6 |  | X | NA | NA | NA |
| Hypothetical protein MAG_4460 | MAG_4460 | Hydrolase, HAD superfamily | 37 |  | X | NA | NA | NA |
| Glycerol kinase, MAG_4470 | MAG_4470 | Lipid metabolism | 13 |  | X | NA | NA | NA |
| Hypothetical transmembrane protein MAG_4530 | MAG_4530 |  | 1 |  | X | NA | NA | NA |
| 50S ribosomal protein L13, MAG_4550 | MAG_4550 | Translation | 4 |  | X | NA | NA | NA |
| 30S ribosomal protein S9, MAG_4560 | MAG_4560 | Translation | 4 |  | X | NA | NA | NA |
| Cation-transporting P-type ATPase, MAG_4590 | MAG_4590 | ABC transporter | 11 |  | X | NA | NA | NA |
| ABC transporter, permease protein, MAG_4600 | MAG_4600 | ABC transporter | 3 |  | X | NA | NA | NA |
| Hypothetical lipoprotein MAG_4720 | MAG_4720 | ABC transporter | 2 |  | X | NA | NA | NA |
| Lipoprotein MAG_4740 | MAG_4740 |  | 14 | X |  | NA | NA | NA |
| Translation initiation factor IF-3, MAG_4750 | MAG_4750 | Translation | 2 |  | X | NA | NA | NA |
| Malate permease, MAG_4890 | MAG_4890 | Membrane transport | 19 |  | X | NA | NA | NA |
| L-lactate dehydrogenase (L-LDH), MAG_4900 | MAG_4900 | Carbohydrate metabolism/Amino acid metabolism | 61 | X | X | NA | NA | NA |
| Phosphate acetyltransferase (phosphotransacetylase), MAG_4920 | MAG_4920 | Carbohydrate metabolism/Amino acid metabolism | 2 |  | X | NA | NA | NA |
| Hexosephosphate transport protein, MAG_4970 | MAG_4970 | Membrane transport | 4 |  | X | NA | NA | NA |
| P80, lipoprotein, MAG_5030 | MAG_5030 | ABC transporter | 176 | X | X | X | X | X |
| Hypothetical protein MAG_5040 | MAG_5040 | Hydrolase, SNc superfamily | 33 | X | X | X | X | X |
| ABC transporter ATP-binding protein, MAG_5050 | MAG_5050 | ABC transporter | 46 |  | X | NA | NA | NA |
| ABC transporter permease protein, MAG_5060 | MAG_5060 | ABC transporter | 8 |  | X | NA | NA | NA |
| ABC transporter permease protein, MAG_5070 | MAG_5070 | ABC transporter | 3 |  | X | NA | NA | NA |
| Lipoprotein MAG_5080 | MAG_5080 |  | 34 | X | X | X | X | X |
| GTPase ObgE, MAG_5090 | MAG_5090 | Hydrolase | 4 |  | X | NA | NA | NA |
| Triosephosphate isomerase, MAG_5140 | MAG_5140 | Carbohydrate metabolism | 2 | X | X | NA | NA | NA |
| Cobalt transporter ATP-binding subunit, MAG_5180 | MAG_5180 | ABC transporter | 2 |  | X | NA | NA | NA |
| 50S ribosomal protein L17, MAG_5190 | MAG_5190 | Translation | 5 |  | X | NA | NA | NA |
| DNA-directed RNA polymerase subunit alpha, MAG_5200 | MAG_5200 | Transcription | 5 |  | X | NA | NA | NA |
| 30S ribosomal protein S61, MAG_5210 | MAG_5210 | Translation | 1 |  | X | NA | NA | NA |
| 30S ribosomal protein S63, MAG_5220 | MAG_5220 | Translation | 4 |  | X | NA | NA | NA |
| Preprotein translocase subunit SecY, MAG_5260 | MAG_5260 | Secretion system/Protein export | 10 |  | X | NA | NA | NA |
| 50S ribosomal protein L15, MAG_5290 | MAG_5290 | Translation | 2 |  | X | NA | NA | NA |
| 30S ribosomal protein S5, MAG_5300 | MAG_5300 | Translation | 33 |  | X | NA | NA | NA |
| 50S ribosomal protein L18, MAG_5310 | MAG_5310 | Translation | 3 |  | X | NA | NA | NA |
| 50S ribosomal protein L6, MAG_5320 | MAG_5320 | Translation | 3 |  | X | NA | NA | NA |
| 50S ribosomal protein L5, MAG_5350 | MAG_5350 | Translation | 3 |  | X | NA | NA | NA |
| 50S ribosomal protein L24, MAG_5360 | MAG_5360 | Translation | 1 |  | X | NA | NA | NA |
| 50S ribosomal protein L14, MAG_5370 | MAG_5370 | Translation | 1 |  | X | NA | NA | NA |
| 30S ribosomal protein S8, MAG_5400 | MAG_5400 | Translation | 25 | X | X | X | X | X |
| 50S ribosomal protein L22, MAG_5410 | MAG_5410 | Translation | 1 |  | X | NA | NA | NA |
| 30S ribosomal protein S69, MAG_5420 | MAG_5420 | Translation | 3 |  | X | NA | NA | NA |
| 50S ribosomal protein L2, MAG_5430 | MAG_5430 | Translation | 29 |  | X | NA | NA | NA |
| 50S ribosomal protein L23, MAG_5440 | MAG_5440 | Translation | 2 |  | X | NA | NA | NA |
| 50S ribosomal protein L4, MAG_5450 | MAG_5450 | Translation | 8 |  | X | NA | NA | NA |
| 50S ribosomal protein L3, MAG_5460 | MAG_5460 | Translation | 22 | X | X | X | X | X |
| 30S ribosomal protein S60, MAG_5470 | MAG_5470 | Translation | 1 |  | X | NA | NA | NA |
| 50S ribosomal protein L27, MAG_5540 | MAG_5540 | Translation | 1 |  | X | NA | NA | NA |
| 30S ribosomal protein S4, MAG_5610 | MAG_5610 | Translation | 13 |  | X | NA | NA | NA |
| DNA gyrase subunit A, MAG_5630 | MAG_5630 | Nucleic acid metabolism | 3 |  | X | NA | NA | NA |
| Modification (methylase) protein of type I restriction-modification system, MAG_5650 | MAG_5650 | Restriction-modification system | 4 |  | X | NA | NA | NA |
| Modification (methylase) protein of type I restriction-modification system HsdM, MAG_5730 | MAG_5730 | Restriction-modification system | 4 |  | X | NA | NA | NA |
| Glutamyl-tRNA synthetase, MAG_5780 | MAG_5780 | Amino acid metabolism | 11 |  | X | NA | NA | NA |
| Signal recognition particle protein, MAG_5820 | MAG_5820 | Secretion system/Protein export | 3 |  | X | NA | NA | NA |
| Phosphoglycerate kinase, MAG_5860 | MAG_5860 | Carbohydrate metabolism | 28 |  | X | NA | NA | NA |
| 5'nucleotidase, MAG_5910 | MAG_5910 | Hydrolase | 53 | X | X | X | X | X |
| Elongation factor G, MAG_5920 | MAG_5920 | Translation | 9 |  | X | NA | NA | NA |
| 30S ribosomal protein S7, MAG_5930 | MAG_5930 | Translation | 12 |  | X | NA | NA | NA |
| 30S ribosomal protein S62, MAG_5940 | MAG_5940 | Translation | 3 |  | X | NA | NA | NA |
| ABC transporter ATP-binding protein, MAG_5960 | MAG_5960 | ABC transporter | 27 |  | X | NA | NA | NA |
| ABC transporter, ATP-binding protein, MAG_5990 | MAG_5990 | ABC transporter | 3 |  | X | NA | NA | NA |
| ABC transporter, ATP-binding protein, MAG_6000 | MAG_6000 | ABC transporter | 10 |  | X | NA | NA | NA |
| DNA-directed RNA polymerase subunit beta', MAG_6110 | MAG_6110 | Nucleic acid metabolism | 61 |  | X | NA | NA | NA |
| DNA-directed RNA polymerase subunit beta, MAG_6120 | MAG_6120 | Nucleic acid metabolism | 73 |  | X | NA | NA | NA |
| 50S ribosomal protein L10, MAG_6190 | MAG_6190 | Translation | 3 |  | X | NA | NA | NA |
| Lipoprotein, MAG_6200 | MAG_6200 | Transferase, choline-bindine protein | 9 | X | X | X | X | X |
| Lysyl-tRNA synthetase, MAG_6210 | MAG_6210 | Amino acid metabolism | 18 |  | X | NA | NA | NA |
| Hypothetical protein MAG_6230 | MAG_6230 | Transcription | 11 |  | X | NA | NA | NA |
| ClpB, MAG_6240 | MAG_6240 | Hydrolase | 6 |  | X | NA | NA | NA |
| 50S ribosomal protein L19, MAG_6270 | MAG_6270 | Translation | 1 |  | X | NA | NA | NA |
| Methionyl-tRNA formyltransferase, MAG_6280 | MAG_6280 | Amino acid metabolism | 2 |  | X | NA | NA | NA |
| 3-keto-L-gulonate-6-phosphate decarboxylase, MAG_6350 | MAG_6350 | Carbohydrate metabolism | 3 |  | X | NA | NA | NA |
| Ascorbate-specific PTS system enzyme IIC, MAG_6380 | MAG_6380 | Membrane transport/Carbohydrate metabolism | 1 |  | X | NA | NA | NA |
| Hypothetical lipoprotein MAG_6520 | MAG_6520 |  | 25 | X | X | X |  | X |
| Hypothetical transmembrane protein MAG_6740 | MAG_6740 | Secretion system/Protein export | 2 |  | X | NA | NA | NA |
| DNA polymerase III subunit gamma and tau, MAG_6870 | MAG_6870 | Nucleic acid metabolism | 2 |  | X | NA | NA | NA |
| Phosphoglyceromutase, MAG_6890 | MAG_6890 | Carbohydrate metabolism | 3 |  | X | NA | NA | NA |
| Hypothetical transmembrane protein MAG_6920 | MAG_6920 |  | 2 |  | X | NA | NA | NA |
| Thymidine kinase, MAG_7010 | MAG_7010 | Nucleotide metabolism | 1 |  | X | NA | NA | NA |
| Aminopeptidase (leucine aminopeptidase), MAG_7020 | MAG_7020 | Amino acid metabolism, hydrolase | 16 |  | X | NA | NA | NA |
| Variable surface lipoprotein V, MAG_7050 | MAG_7050 |  | 6 | X | X | X | X | X |
| Variable surface lipoprotein W, MAG_7060 | MAG_7060 |  | 4 | X | X | X | X | X |
| Variable surface lipoprotein A, MAG_7070 | MAG_7070 |  | 11 | X | X | X | X | X |
| Variable surface lipoprotein Y, MAG_7080 | MAG_7080 |  | 74 | X | X | X |  | X |
| Variable surface lipoprotein U, MAG_7090 | MAG_7090 |  | 1 | X | X | X | X | X |
| Variable surface lipoprotein D, MAG_7100 | MAG_7100 |  | 27 | X | X | X | X | X |
| Amidase, MAG_7170 | MAG_7170 | Amino acid metabolism | 1 |  | X | NA | NA | NA |
| 1-acyl-SN-glycerol-3-phosphate acyltransferase, MAG_7220 | MAG_7220 | Lipid metabolism | 8 |  | X | NA | NA | NA |
| Tyrosyl-tRNA synthetase 1, MAG_7300 | MAG_7300 | Amino acid metabolism | 2 |  | X | NA | NA | NA |
| Hypothetical transmembrane protein MAG_7360 | MAG_7360 |  | 2 |  | X | NA | NA | NA |
| DNA gyrase subunit B, MAG_7370 | MAG_7370 | Nucleic acid metabolism | 3 |  | X | NA | NA | NA |
| Hypothetical protein MAG_7400 | MAG_7400 | Transcription | 5 |  | X | NA | NA | NA |
| Cation-transporting P-ATPase, MAG_7420 | MAG_7420 | Energy metabolism | 3 |  | X | NA | NA | NA |
| ABC transporter ATP-binding protein, MAG_7430 | MAG_7430 | ABC transporter | 1 |  | X | NA | NA | NA |
| ABC transporter permease protein, MAG_7440 | MAG_7440 | ABC transporter | 19 |  | X | NA | NA | NA |
| Cell division protein ftsH-like protein, MAG_7450 | MAG_7450 | Cell growth | 11 |  | X | NA | NA | NA |
| Glycerol ABC transporter, ATP-binding protein, MCAP_0454 | MCAP_0454 | ABC transporter | 9 |  | X | NA | NA | NA |
| Hypothetical protein MYPU_3820 | MYPU_3820 |  | 5 |  | X | NA | NA | NA |
