# Supplementary material for: The liposoluble proteome of Mycoplasma agalactiae: an insight into the minimal protein complement of a bacterial membrane
Source: BMC Microbiol. 2010 Aug 25;10:225. doi: 10.1186/1471-2180-10-225 (PMC2941501; doi:10.1186/1471-2180-10-225)
Supplement: Additional file 8 — Proteins identified in the M. agalactiae proteome potentially resulting from Horizontal Gene Transfer events with M. mycoides subsp. mycoides and M. capricolum subsp. capricolum. [file 1471-2180-10-225-S8.DOC]

### Additional file 8. Proteins identified in the *M. agalactiae* PG2^T^ proteome potentially resulting from Horizontal Gene Transfer events with *M. mycoides* subsp*. mycoides* and *M. capricolum* subsp. *capricolum*.

| **Name** | **Locus** | **Homolog in *M. mycoides* subsp. *mycoides*** | **Homolog in *M. capricolum* subsp. *capricolum*** | **Other homologies** | **Comments** |
| --- | --- | --- | --- | --- | --- |
| Oligopeptide ABC transporter, substrate-bindingprotein (OppA), lipoprotein, MAG_0380 | MAG_0380 | MSC_0964 | MCAP0116 | Several homologs from the Hominis group | MAG having homolog in the Hominis group |
| D-lactate dehydrogenase MAG_1490 | MAG_1490 | MSC_0034 | MCAP0460 | Homolog in *M. penetrans* also highly similar | MAG having homolog in the mycoides cluster and other bacteria but not in the Hominis group |
| Hypothetical protein MAG_1670 | MAG_1670 | MSC_0240 | MCAP0033 | No homolog outside of the mycoides cluster | MAG CDS having no homolog outside of the mycoides cluster |
| Hypothetical protein MAG_2220 | MAG_2220 | MSC_0519 | MCAP0451 | Several homologs | MAG having homolog in the Hominis group |
| Hypothetical protein MAG_2340 | MAG_2340 | MSC_0519 | MCAP0451 | Several homologs | MAG having homolog in the Hominis group |
| Lipoprotein, MAG_2430 | MAG_2430 | MSC_1005 | MCAP0268 | No homolog outside of the mycoides cluster | MAG CDS having no homolog outside of the mycoides cluster |
| Alkylphosphonate ABC transporter substrate-binding protein, MAG_2690 | MAG_2690 | MSC_0790 | MCAP0731 | Homolog from the Hominis group probably lost | MAG having homolog in the Hominis group |
| Putative transmembrane protein, MAG_2920 | MAG_2920 | MSC_0620 | MCAP0357 | Homolog from the Hominis group probably lost | MAG having homolog in the Hominis group |
| Endopeptidase O, MAG_3680 | MAG_3680 | MSC_0696 | MCAP0466 | Homolog in non mollicute bacteria | MAG having homolog in the mycoides cluster and other bacteria but not in the Hominis group |
| Glycerol kinase, MAG_4470 | MAG_4470 | MSC_0258 | MCAP0218 | Homolog from the Hominis group probably lost | MAG having homolog in the Hominis group |
| ABC transporter, permease protein, MAG_4600 | MAG_4600 | MSC_0324 | MCAP0798 | No paralog | MAG having homolog in the Hominis group |
| Malate permease, MAG_4890 | MAG_4890 | MSC_0035 | MCAP0780 | Homolog in *M. penetrans* | MAG having homolog in the mycoides cluster and other bacteria but not in the Hominis group |
| Hexosephosphate transport protein, MAG_4970 | MAG_4970 | MSC_0118 | MCAP0814 | Homolog from the Hominis group probably lost | MAG having homolog in the Hominis group |
| Hypothetical protein MAG_6520 | MAG_6520 | MSC_0519 | MCAP0451 | Several homologs | MAG having homolog in the Hominis group |
| Glycerol ABC transporter, ATP-binding protein, MCAP_0454 | MAG_2310 | MSC_0516 |  | Several homologs | MAG having homolog in the Hominis group |
