# Supplementary material for: The liposoluble proteome of Mycoplasma agalactiae: an insight into the minimal protein complement of a bacterial membrane
Source: BMC Microbiol. 2010 Aug 25;10:225. doi: 10.1186/1471-2180-10-225 (PMC2941501; doi:10.1186/1471-2180-10-225)
Supplement: Additional file 9 — Proteins identified in the M. agalactiae proteome potentially resulting from Horizontal Gene Transfer events with other bacteria. [file 1471-2180-10-225-S9.DOC]

### Additional file 9. Proteins identified in the *M. agalactiae* PG2^T^proteome potentially resulting from Horizontal Gene Transfer events with other bacteria.

| **Name** | **Locus** | **Putative HGT with organisms of** | **Comments** |
| --- | --- | --- | --- |
| Hypothetical protein MAG_0250 | MAG_0250 | Firmicutes/Proteobacteria | No paralog, no homolog in Mollicutes |
| Putative phosphoketolase, MAG_1230 | MAG_1230 | Firmicutes (Lactobacillales) | No paralog, no homolog in *Mollicutes* |
| Alcohol dehydrogenase, MAG_2740 | MAG_2740 | Firmicutes/Proteobacteria | Paralog MAG_4280, MAG_4340 (also HGT acquired), no homolog in the Hominis group |
| Alcohol dehydrogenase, MAG_4340 | MAG_4340 | Firmicutes/Proteobacteria | Paralog MAG_4280, MAG_2740 (also HGT acquired), no homolog in the Hominis group |
| Hypothetical protein MAG_4460 | MAG_4460 | Pneumoniae group | No homolog in the Hominis group, homolog only in U. urealyticum |
| Modification (methylase) protein of type irestriction-modification system, MAG_5650 | MAG_5650 | Firmicutes | Paralog MAG_5730 (also HGT acquired), paralog from the Hominis group probably lost |
| Modification (methylase) protein of type irestriction-modification system HsdM, MAG_5730 | MAG_5730 | Firmicutes | Paralog MAG_5730 (also HGT acquired), paralog from the Hominis group probably lost |
